# *Lytopylus* Förster (Hymenoptera, Braconidae, Agathidinae) species from Costa Rica, with an emphasis on specimens reared from caterpillars in Area de Conservación Guanacaste

**DOI:** 10.3897/zookeys.130.1569

**Published:** 2011-09-24

**Authors:** Michael J. Sharkey, Stephanie Clutts, Erika M. Tucker, Daniel Janzen, Winnie Hallwachs, Tanya Dapkey, M. Alex Smith

**Affiliations:** 1 Department of Entomology, University of Kentucky, S-225 Ag. Sci. N., Lexington, KY 40546; 2 Department of Biology, University of Pennsylvania, 3740 Hamilton Walk, Philadelphia, PA 19104; 3 ADepartment of Integrative Biology and the Biodiversity Institute of Ontario, University of Guelph, 50 Stone Road East, Guelph, Ontario, Canada, N1G 2W1

**Keywords:** parasitoids, biology, endoparasitoids, koinobiont, Lepidoptera

## Abstract

Twelve species of Costa Rican *Lytopylus* are treated; these include all species reared from Lepidoptera caterpillars in Area de Conservación Guanacaste, Costa Rica, over 32 years of caterpillar inventory, as well as two species recorded in the literature as occurring in Costa Rica. Ten new species are described, i.e., *Lytopylus bradzlotnicki*, *Lytopylus colleenhitchcockae*, *Lytopylus gregburtoni*, *Lytopylus jessicadimauroae*, *Lytopylus jessiehillae*, *Lytopylus mingfangi*, *Lytopylus rebeccashapleyae*, *Lytopylus robpringlei*, *Lytopylus sandraberriosae*, *Lytopylus vaughntani*. The following species are transferred to *Lytopylus*: *Metriosoma flavicalcar* Enderlein 1920 to *Lytopylus flavicalcar* **comb. n.**; *Bassus macadamiae* Briceño and Sharkey 2000 to *Lytopylus macadamiae* **comb. n.**; *Metriosoma bicarinatum* Enderlein 1920 to *Lytopylus bicarinatum* **comb. n.**; *Metriosoma brasiliense* Enderlein 1920 to *Lytopylus brasiliense* **comb. n.**; *Bassus tayrona* Campos 2007 to *Lytopylus tayrona* **comb. n.**; *Microdus femoratus* Cameron 1887 to *Lytopylus femoratus* **comb. n.**; *Microdus melanocephalus* Cameron 1887 to *Lytopylus melanocephalus* **comb. n.**; *Bassus pastranai* Blanchard 1952 to *Lytopylus pastranai* **comb. n.**; *Agathis nigrobalteata* Cameron 1911 to *Lytopylus nigrobalteatus* **comb. n.** Two keys to species of *Lytopylus* are presented, one interactive and the other static.

## Introduction

This is the first of a series of articles to revise the species of Agathidinae reared from lepidopteran caterpillars as part of the inventory of the caterpillars and their parasitoids of Area de Conservación Guanacaste (ACG), northwestern Costa Rica (http://janzen.sas.upenn.edu; http://www.acguanacaste.ac.cr; Janzen et al. 2009). It includes keys, in traditional and interactive formats, to the reared species of *Lytopylus* Förster and to the two species of *Lytopylus* previously recorded from Costa Rica. This is not meant to be a comprehensive treatment of the Costa Rican fauna of *Lytopylus*. A conservative estimate, based on extensive collections at INBio, the Universidad de Costa Rica, and the University of Kentucky, is that there are a minimum of 50 species occurring in the country. Here we treat only 12.

*Lytopylus*, though proposed in 1862, has spent most of its existence as a junior synonym of *Bassus* Fabricius, *Microdus* Nees, and *Agathis* Latreille. Sharkey et al. (2009) removed it from synonymy in their revision of the Oriental genera of Agathidinae. New World members of the genus may be distinguished from other agathidines by the sculpture of the third metasomal median tergite and by the structure of the propodeal foramen. See the first couplet of the key included in this publication for specifics. The sistergroup to *Lytopylus* is *Braunsia* Kriechbaumer which is restricted to the Old World and is mostly tropical or subtropical (Sharkey et al. 2006).

The source files for the keys, descriptions, illustrations, DNA sequence and distributional data are all freely available to future researchers who may wish to build on this beginning. The detailed specimen records are available by search of the individual specimen DHJPARxxxxxxx voucher codes at http://janzen.bio.upenn.edu/caterpillars/database.lasso. DNA trace files and primer information are available through the Barcode of Life Datasystem (BOLD) [Ratnasingham and Hebert 2007] at http://www.boldsystems.org.

## Methods

Phenetic and phylogenetic trees, using 658 base pairs of cytochrome *c* oxidase (COI) data, were constructed using neighbor-joining (NJ), maximum parsimony (MP) and Bayesian methods. MP was performed using TNT (Goloboff et al. 2008). A traditional search with 100 random addition sequences followed by branch-swapping, saving 100 trees per replication, was performed. 1000 bootstrap replications were used to estimate branch reliability. The Bayesian analysis was performed using MrBayes v3.1.2 (Ronquist and Huelsenbeck 2003). Best-fitting DNA substitution models were determined using MrModeltest2.2 (Nylander 2004). The general time reversible model of evolution with a parameter for invariant sites and rate heterogeneity modeled under a gamma distribution (GTR+I+G) was determined as the best-fitting model.

The Bayesian analysis consisted of two independent Bayesian MCMC runs initiated from different random starting trees. The analysis ran for 2,000,000 generations, reaching a topological similarity criterion of 0.01; trees were sampled every 500 generations. 25% of the trees from each run were removed as burn-in upon topological convergence.

The NJ tree was produced from PAUP* (Swofford 2002) using default settings.

Sequence data, trace files, and field data are available on BOLD (www.barcodinglife.org). Additional collection information is deposited at http://janzen.sas.upenn.edu, and all sequences have been deposited in the GenBank database.

The dichotomous key, descriptions, and the interactive key (Appendices 1–3) were generated using DELTA Editor Dallwitz et al. (1999), DELTA Dallwitz et al. (1993), and Intkey Dallwitz et al. (1995).

This set of 10 species of ACG *Lytopylus*, represented by 76 specimens, is the yield from rearing 498,000+ wild-caught caterpillars of about 6,000 species from ACG dry forest, cloud forest, and rain forest, and a variety of intergrades, over 32 years, beginning 1978 (Janzen et al. 2009). At least 90% of these caterpillars were, in some instar, large enough to host a larva of *Lytopylus*. The names of the caterpillars, from which the degree of host-specificity mentioned below is determined, is the collective contribution of more than 100 taxonomists and parataxonomists working with both caterpillar morphology and food plant biology, and correlated adult morphology; additionally, nearly all of the caterpillar species have now had their adults DNA-barcoded as yet another check on identification (Janzen et al. 2009, 2011).

**Abbreviations and codes.**There are numerous codes in the text. Codes beginning with an “H” and followed by numbers are unique identifiers used for specimens in the Sharkey lab at the University of Kentucky, and in the specimen database (http://sharkeylab.org/taxabank/home.php), e.g., H6408. Codes beginning with DHJPAR, e.g., DHJPAR0036705, are used by Janzen as unique identifiers for the parasitoids reared by the ACG caterpillar inventory. Some of the Lepidoptera hosts are incompletely identified; however, they also have unique names such as *Desmia* Solis19 (which is an interim name for *Desmia* species 19 as determined by M. Alma Solis of the USDA Systematic Entomology Laboratory, Washington, D.C.). These names will be updated in the Janzen database (http://janzen.sas.upenn.edu/caterpillars/database.lasso) when the species is baptized with a formal scientific name, but the interim name, in this case *Desmia* Solis19, will remain searchable in that database. Host caterpillars are uniquely identified by their own voucher code system, which is recognizable by YY-SRNP-XXXXX where “YY” is the two digit year and “XXXXX” is a unique number within that year. DNA barcodes associated with each specimen via the DHJPAR code also each have a searchable accession on BOLD (e.g. ASBC979-07 for DHJPAR0021167).

Abbreviations used for specimen depositories are as follows:

AEIAmerican Entomological Institute, Gainesville, Florida, USA.

HICHymenoptera Institute Collection, University of Kentucky, Department of Entomology, Lexington, Kentucky, USA.

INBioNational Biodiversity Institute, Santo Domingo de Heredia, Costa Rica.

## Results

**Phylogeny and Biology:** *Lytopylus* is an unusual genus of Agathidinae in that it has a cosmopolitan distribution and a wide range of host higher taxa. Most genera of Agathidinae found in the neotropics are restricted to the New World. When there are exceptions to this rule, they are in the form of Holarctic genera that appear to have dispersed into cool, high altitude areas in the northern neotropics, e.g., *Agathis*, which is found at high altitudes in Costa Rica (Sharkey, unpublished). Most genera of Agathidinae also seem to be restricted to one or a few closely-related families of Lepidoptera; however, the collective set of *Lytopylus* species attack a wide range of host families. Even within the small sample of ten reared species of *Lytopylus* represented here, five host families are attacked, i.e., Crambidae, Elachistidae, Pyralidae, Thyrididae, and Tortricidae. However, all of these are leaf-rolling and leaf-tying small caterpillars. *Lytopylus* are conspicuous in not using species of caterpillars that feed exposed on leaf surfaces (e.g., butterflies and macro-moths), and in not using large caterpillars. However, their absence from leaf-nest-occupying Hesperiidae (over 500 species reared from ACG, Janzen et al. 2011) remains a puzzle, especially since *Bassus brooksi*, a reasonably close relative to *Lytopylus*, attacks a very specific, large subset of ACG Hesperiidae (Janzen et al. 1998).

**Figure 1.**
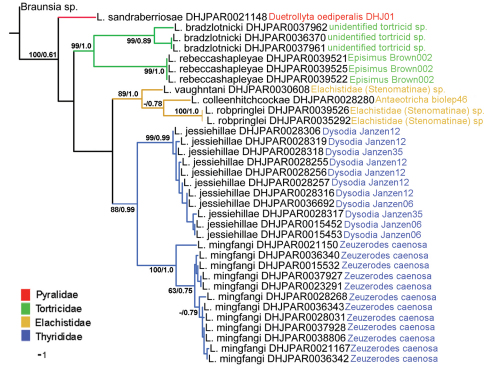
NJ tree of the COI barcode region for eight of the 10 *Lytopylus* treated here. Branch support = parsimony bootstrap/Bayesian posterior probabilities (bootstrap values less than 60 are not illustrated). Branches and host names are colored to reflect host family.

Figure 1 presents the NJ tree, which was much more resolved than were the phylogenetic trees produced by MP and Bayesian analyses. However all three methods were congruent with each other, both in that individuals of what are believed to be a single species grouped together, and the groups bear the same relative position to each other. We mapped the support values of the Bayesian and MP analyses on the NJ tree. Branches without values are those that collapsed in the phylogenetic analyses.

Individual species of *Lytopylus* tend to be rather host-specific. Of the species treated here all but one, *Lytopylus jessiehillae*, are found on a single host species. And even the hosts of *Lytopylus jessiehillae* are very similar to each other. Moreover, the results of our phylogenetic analyses (Fig. 1) show that host family is directly correlated to phylogenetic history, with sister species of *Lytopylus* sharing the same host family in every case (three). For members of *Lytopylus*, as with most other closely examined genera of Agathidinae in ACG, the regular use of multiple species of hosts by a single species is the exception and host range is phylogenetically constrained, albeit at a lower taxonomic level than appears to be the case with other Agathidinae.

**Taxonomy:**Sharkey et al. (2009) distinguished *Lytopylus* from *Bassus* and removed it from synonymy, however only three Neotropical species were formally transferred, i.e., *Lytopylus boliviensis* (Szepligeti), *Lytopylus bicristatus* (Enderlein), and *Lytopylus facetus* (Enderlein). The following is a list of all of the non-Costa Rican Neotropical species of *Lytopylus* known to MJS that are not treated above or elsewhere in this paper; all are new combinations: *Metriosoma bicarinatum* Enderlein 1920 = *Lytopylus bicarinatum*; *Metriosoma brasiliense* Enderlein 1920 = *Lytopylus brasiliense; Bassus tayrona* Campos 2007 = *Lytopylus tayrona*; *Microdus femoratus* Cameron 1887 = *Lytopylus femoratus*; *Microdus melanocephalus* Cameron 1887 = *Lytopylus melanocephalus*; *Bassus pastranai* Blanchard 1952 = *Lytopylus pastranai*; *Agathis nigrobalteata* Cameron 1911 = *Lytopylus nigrobalteatus*.

**Figure 2.**
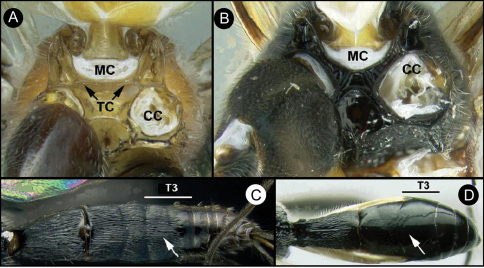
**a** *Lytopylus* sp. Metasomal cavity (MC) situated entirely dorsal to coxal cavities (CC); a wide, high, straight, transverse carinae (TC) between MC and CC **b** *Therophilus* sp. MC situated partly between CC **c** *Lytopylus* sp. median tergite 3 (T3) showing extensive striae in anterior half **d** *Therophilus* sp. T3 smooth.

Members of *Lytopylus* can be distinguished from other agathidines in the New World with two characters. Most species (90%) of *Lytopylus* have some sculpture (rugosity, striae, etc.) on the third median tergite (Fig. 2c, T3), whereas other New World agathidines have T3 completely smooth or rarely with some very weak coriaceous sculpture (Fig. 2d). The 10% of *Lytopylus* that lack sculpture on T3 can be distinguished by the position of the cavity on the mesosoma (MC) into which the metasoma inserts. In species of *Lytopylus* (Fig. 2a) it is positioned completely above the coxal cavities of the hind leg and separated from them by a straight transverse carina (TC). In other agathidines (except *Braunsia*, an Old World genus) the ventral margin of MC is positioned below the dorsal margin of CC and a straight transverse carinae is lacking (Fig. 2b).

### Key to Costa Rican species of Lytopylus (♀ and ♂)

Note: This key is restricted to those species reared in ACG and those that are recorded in the literature as occurring in Costa Rica. Since there are a minimum of 50 species of (mostly undescribed) *Lytopylus* occurring in Cost Rica, users of this key should compare specimens with the descriptions and images after identification using the key below or the interactive key found here.

**Table:** 

1a	Metasoma mostly or entirely melanic	2
1aa	Metasoma mostly or entirely pale, yellow, orange, or reddish brown	4
	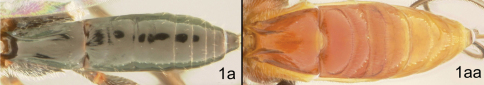	
2	Forewing evenly and deeply infuscate	*Lytopylus vaughntani* sp. n.
2aa	Forewing mostly hyaline, sometimes weakly infuscate basally	3
	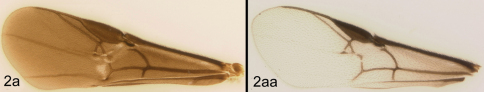	
3a	Notauli sculptured with crenulae in anterior half	*Lytopylus bradzlotnicki* sp. n.
3aa	Notauli mostly or entirely smooth in anterior half	*Lytopylus rebeccashapleyae* sp. n.
	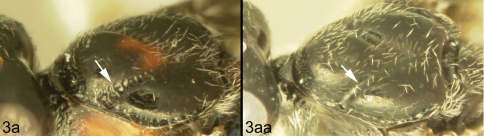	
4a	Median syntergite 2+3 forming a carapace that almost covers all more posterior terga; posterior margin of median syntergite 2+3 convex; 4b. median areola of propodeum well-defined and smooth with a rounded anterior border	11
4aa	Median syntergite 2+3 not forming a carapace that almost covers all more posterior terga; posterior margin of median syntergite 2+3 straight; 4bb. median areola of propodeum not as above, either pentagonal or triangular with sharp angles, or absent	5
	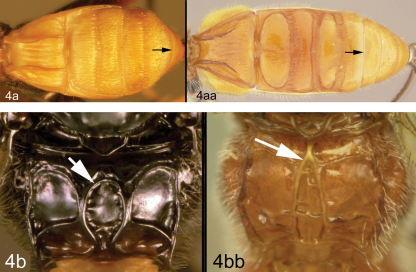	
5a	Forewing banded	*Lytopylus sandraberriosae* sp. n.
5aa	Forewing not banded, mostly or entirely infuscate	6
	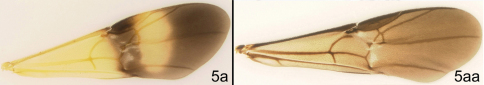	
6a	Mesoscutum mostly or entirely pale, orange or reddish yellow	7
6aa	Mesoscutum mostly or entirely melanic	8
	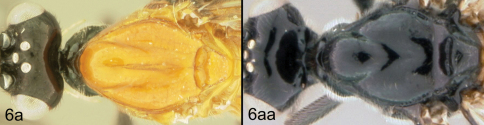	
7a	Propodeum mostly smooth, lacking areolae	*Lytopylus flavicalcar* (Enderlein)
7aa	Propodeum sculptured with areolae	*Lytopylus robpringlei* sp. n.
	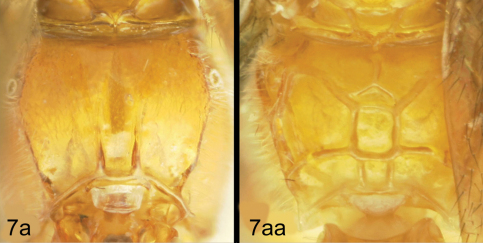	
8a	Hind leg almost entirely black	9
8aa	Hind leg reddish in large part, either coxa or femur or both reddish	10
	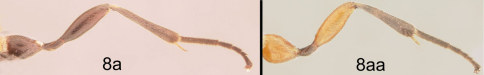	
9a	Occiput relatively sharply angled at mid-height	*Lytopylus gregburtoni* sp. n.
9aa	Occiput evenly rounded	*Lytopylus colleenhitchcockae* sp. n.
	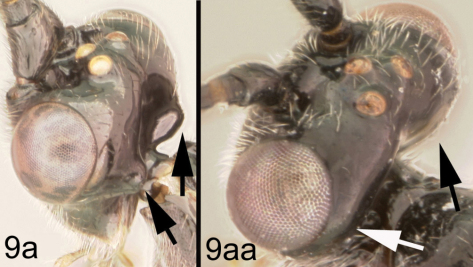	
10a	Gena acute posterolaterally; 10b. median syntergite 2+3 not heavily sculptured	*Lytopylus jessicadimauroae* sp. n.
10aa	Gena rounded or obtuse posteroventrally; 10bb. median syntergite 2+3 heavily sculptured	*Lytopylus macadamiae* (Briceño and Sharkey)
	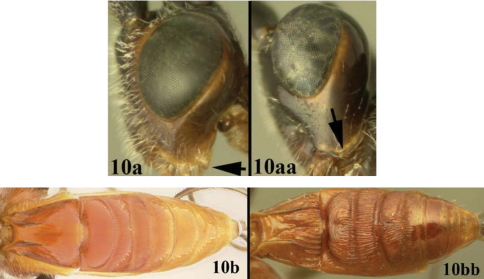	
11a	Third lobe of median syntergite 2+3 entirely striate	*Lytopylus jessiehillae* sp. n.
11aa	Third lobe of median syntergite 2+3 smooth, at least posteriorly	*Lytopylus mingfangi* sp. n.
	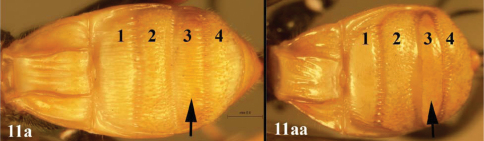	

## Species treatments

### Lytopylus bradzlotnicki

Sharkey sp. n.

urn:lsid:zoobank.org:act:D484B71E-5CE6-48DF-AB4E-01F5D673495E

http://species-id.net/wiki/Lytopylus_bradzlotnicki

Figs 3 4

#### Description.

**Figure 3.**
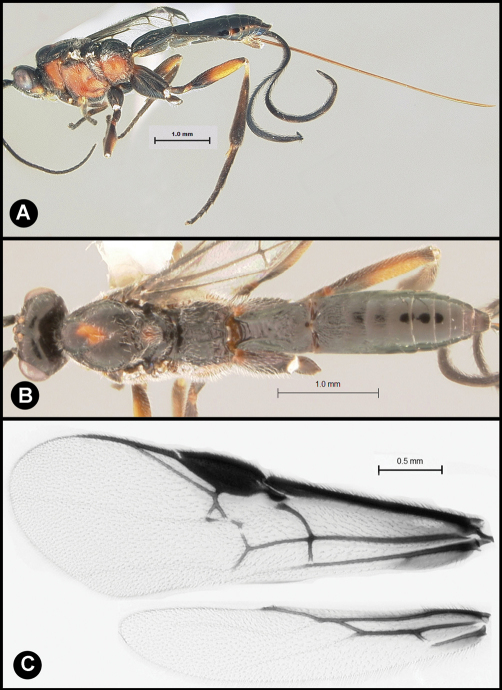
*Lytopylus bradzlotnicki* sp. n. Holotype **a** lateral habitus **b** dorsal habitus **c** wings.

**Figure 4.**
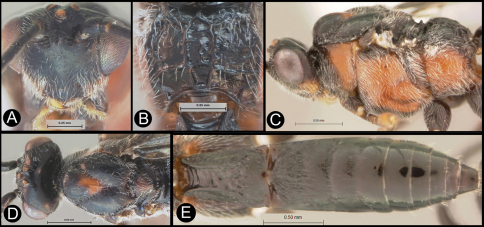
*Lytopylus bradzlotnicki* sp. n. Holotype **a** anterior head **b** dorsal propodeum **c** lateral head and mesosoma **d** dorsal head and mesosoma **e** dorsal metasoma.

Body length 4.1 – 4.5 mm. Ovipositor length 3.9 – 4.2 mm. Gena rounded or with an obtuse angle posterolaterally. Longitudinal groove on interantennal prominence absent. Protuberances on occiput absent. Propodeum with carinae forming areolae, median areola not rounded anteriorly. Notauli well-impressed, with crenulae extending well along its length. Posterior margin of syntergum 2+3 straight. Median syntergite 2 + 3 weakly longitudinally rugose in anterior half to two-thirds, smooth posteriorly. Forewing clear except base slightly infuscate. Color as in Figs 3, 4. Color variation: Pale color of mesonotum may be more extensive or completely absent. Pale color of pronotum may be much reduced but always extensive on meso-and metapleuron. Pale color of hind femur varies from almost completely pale to almost completely melanic.

#### Molecular data.

BOLD process ID/ Janzen parasitoid voucher/GenBank accession:

ASHYC4707-10/DHJPAR0037962/JN034688;

ASHYD1561-09/DHJPAR0036370/JN034687;

ASHYC4706-10/DHJPAR0037961/JN034686-

#### Distribution.

Guanacaste Province and Alajuela Province, Costa Rica. Click here for a distribution map.

#### Biology.

All three rearings are from an unidentified Tortricidae of the same species feeding on rain forest *Psidium guajava* (Myrtaceae). Since this food plant is widely introduced into ACG pastures, it is assumed that the “natural” food plant of this caterpillar is some other plant and therefore the *Lytopylus* may be there as well. This tortricid lightly silks two overlapping leaves together and lives in a silk and fecal pellet tangle between them, while skeletonizing the leaf. Therefore, the wasp likely accesses the caterpillar by ovipositing through the leaf. This wasp and *Lytopylus rebeccashapleyae* are the only *Lytopylus* reared from more than 5,000 ACG tortricid rearings of dry forest and rain forest caterpillars. *Austroearinus* (Agathidinae) also has been reared in very low numbers from other species of tortricids than those that are *Lytopylus* hosts.

#### Etymology.

*Lytopylus bradzlotnicki* is named in honor of Brad Zlotnick of San Diego, California, who has enthusiastically supported the conservation of the ACG rain forest occupied by this parasitoid wasp.

#### Material examined.

**Holotype**: ♀, H6409 (DHJPAR0037961) Costa Rica: Guanacaste: Area de Conservación Guanacaste: Sector Pitilla: Estacion Pitilla: 7.ii.2010, 10.9893N, 85.4258W, 675m [AEI].

**Paratypes** [AEI, HIC, INBio]: Costa Rica: Alajuela: Area de Conservación Guanacaste: Sector Rincon Rainforest: Estacion Caribe: 26.v.2009, 10.9018N, 85.2749W, 415m: ♀, H7078 (DHJPAR0036370). Sector Pitilla: Estacion Pitilla: 7.ii.2010, 10.989N, 85.425W, 675m: ♀, H7071 (DHJPAR0037962). San Carlos: P.N. Arenal: Colada: 1.ii-iii.2000, 10.635N, 84.47W, 600m: ♀ H7081, ♀ H7070, ♀ H7073, ♀ H7072, ♀ H7069.

### Lytopylus colleenhitchcockae

Sharkey sp. n.

urn:lsid:zoobank.org:act:6432177D-C7D2-4433-A0C2-FA0E1EA572D8

http://species-id.net/wiki/Lytopylus_colleenhitchcockae

Figs 5 6

#### Description.

**Figure 5.**
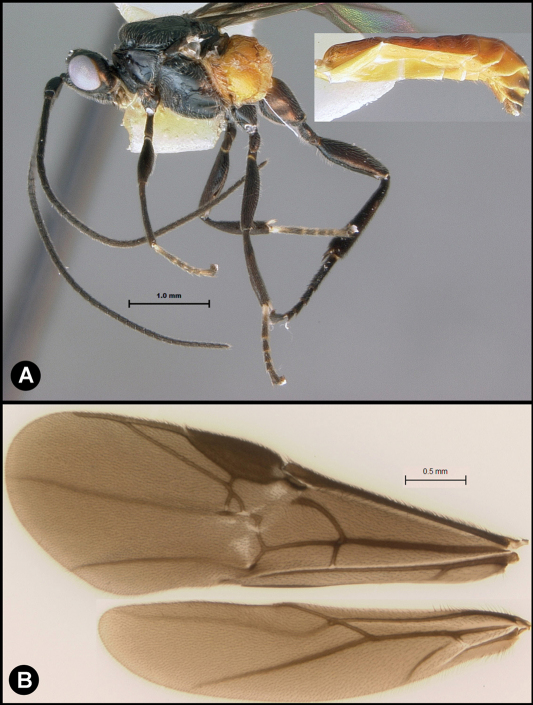
*Lytopylus colleenhitchcockae* sp. n. Holotype **a** lateral habitus **b** wings.

**Figure 6.**
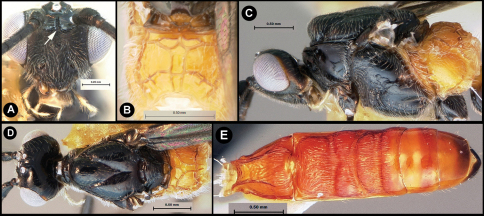
*Lytopylus colleenhitchcockae* sp. n. Holotype **a** anterior head **b** dorsal propodeum **c** lateral head and mesosoma **d** dorsal head and mesosoma **e** dorsal metasoma.

Body length 5.2 mm. Gena acute posterolaterally, or rounded or with an obtuse angle posterolaterally. Longitudinal groove on interantennal prominence present. Protuberances on occiput absent. Propodeum with carinae forming areolae, median areola not rounded anteriorly. Notauli well-impressed, with one or two crenulae restricted to extreme anterior apex along border of mesoscutum. Posterior margin of syntergum 2+3 straight. Median syntergite 2 + 3 longitudinally striate except extreme apex smooth. Forewing mostly or entirely infuscate. Color as in Figs 5, 6.

#### Molecular data.

BOLD process ID/Janzen parasitoid voucher/GenBank accession:

ASHYF042-09/DHJPAR0028280/JN034689.

#### Distribution.

Guanacaste Province, Costa Rica.Click here for a distribution map.

#### Biology.

**Figure 7.**
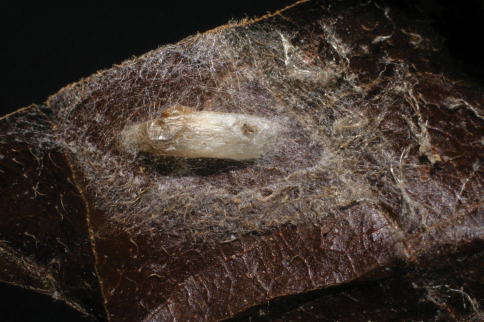
*Lytopylus colleenhitchcockae* cocoon.

The single rearing is from *Antaeotricha* biolep46 (Elachistidae, Stenomatinae) feeding on rain forest *Inga chocoensis* (Fabaceae). It is the only *Lytopylus* reared from *Inga*-eating caterpillars in ACG. The caterpillar lightly webs two overlapping leaves as described for the elachistid hosts of *Lytopylus robpringlei*. The flimsy white wasp cocoon (Fig. 7) is spun between the same two leaves and the wasp eclosed 10 days after spinning.

The inventory has reared 101 wild-caught Elachistidae from *Inga chocoensis* and this is the sole agathidine wasp obtained. The score for all 1,846 rearings of Elachistidae from all species of *Inga* during 33 years is this single *Lytopylus* plus five *Austroearinus* spp. (to be treated elsewhere).

#### Etymology.

Named in honor of Colleen Hitchcock of Boston, Massachusetts, who has enthusiastically supported the conservation of the ACG forest occupied by this parasitoid wasp.

#### Material examined.

**Holotype**: ♂, H6836 (DHJPAR0028280) Costa Rica: Guanacaste: Area de Conservación Guanacaste: Sector Pitilla: Ingas: 11.0031N, 85.4204W, 580m, 2.x.2007 [AEI].

### Lytopylus flavicalcar

(Enderlein) comb. n.

http://species-id.net/wiki/Lytopylus_flavicalcar

Figs 8 9

Metriosoma flavicalcar Enderlein 1920

#### Description.

**Figure 8.**
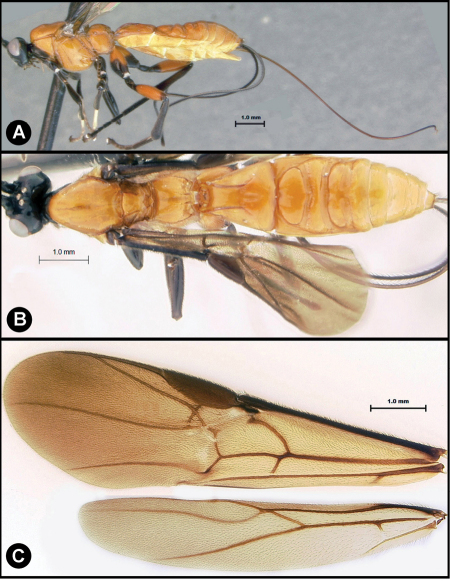
*Lytopylus flavicalcar* (Enderlein) **a** lateral habitus **b** dorsal habitus **c** wings.

**Figure 9.**
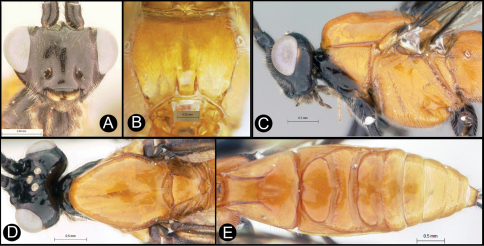
*Lytopylus flavicalcar* (Enderlein) **a** anterior head **b** dorsal propodeum **c** lateral head and mesosoma **d** dorsal head and mesosoma **e** dorsal metasoma.

Body length 8.2 – 9.5 mm. Ovipositor length 9.0 – 10.5 mm. Gena rounded or with an obtuse angle posterolaterally. Longitudinal groove on interantennal prominence absent. Protuberances on occiput absent. Propodeum mostly smooth, lacking areolae. Notauli absent, or indicated by a weak smooth medio-posterior depression. Posterior margin of syntergum 2+3 straight. Median syntergite 2 + 3 smooth, lacking microsculpture. Forewing mostly or entirely infuscate. Color as in Figs 8, 9. Color variation: I have only included specimens here that are colored almost exactly as is the holotype. There are many other specimens from Costa Rica (INBio) that seem structurally to be very similar but differ primarily in that the hind femur is completely melanic. These may or may not be conspecific.

#### Distribution.

Puntarenas and Alajuela provinces, Costa Rica. Click here for a distribution map.

#### Biology.

Unknown.

#### Material examined.

**Holotype:** ♀, Colombia, Fusagasuga (about 30 km. S. of Bogota), E. Pehlke S. (collector), Museum of the Institute of Zoology, Polish Academy of Science**.**

#### Non-type material:

[HIC, InBio]: Costa Rica: Puntarenas: Buenos Aires: Estacion Altamira: Sendero Los Gigantes: 10.469N, 84.339W, 1450m, 15.vii.-16.viii.2000.♀ H6407, ♀ H6465. Alajuela: Upala: Albergue Heliconias: Sendero Mirador, 10.891N, 85.015W, 1000m, 20–31.viii.2000: ♀ H7082

### Lytopylus gregburtoni

Sharkey sp. n.

urn:lsid:zoobank.org:act:CED31100-2B25-44E4-8F89-13EF5CD5745E

http://species-id.net/wiki/Lytopylus_gregburtoni

Figs 10 11

#### Description.

**Figure 10.**
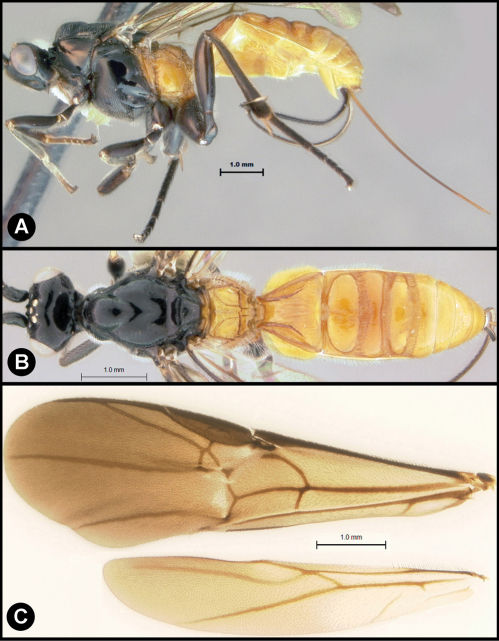
*Lytopylus gregburtoni* sp. n. **a** lateral habitus **b** dorsal habitus **c** wings.

**Figure 11.**
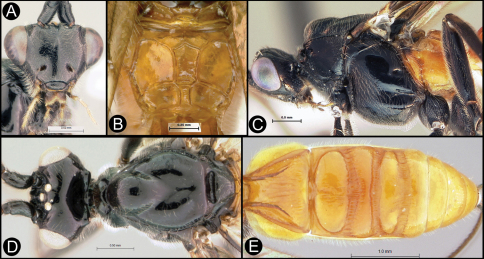
*Lytopylus gregburtoni* sp. n. **a** anterior head **b** dorsal propodeum **c** lateral head and mesosoma **d** dorsal head and mesosoma **e** dorsal metasoma.

Body length 6.4 – 7.9 mm. Ovipositor length 3.7 – 5.5 mm. Gena acute posterolaterally, or rounded or with an obtuse angle posterolaterally. Longitudinal groove on interantennal prominence present. Protuberances on occiput present. Propodeum with carinae forming areolae, median areola not rounded anteriorly. Notauli well-impressed, smooth without crenulae, or with one or two crenulae restricted to extreme anterior apex along border of mesoscutum. Posterior margin of syntergum 2+3 straight. Median syntergite 2 + 3 mostly smooth with longitudinal striae in the three transverse depressions and sometimes striate anteromedially anterad first transverse depression. Forewing mostly or entirely infuscate. Color as in Figs 10, 11. Color variation: This species is very consistent in color across all 18 specimens examined.

#### Distribution.

Puntarenas Province and Alajuela Province, Costa Rica. Click here for a distribution map.

#### Biology.

The single rearing is from *Desmia* Solis19 (Crambidae, Spilomelinae) feeding on *Psychotria panamensis* (Rubiaceae) in ACG rain forest. The caterpillar makes a large sloppy leaf roll and lives within it, among leaf fragments and fecal pellets. The wasp spins its cocoon inside the leaf roll. This caterpillar has been reared 150+ times from a diverse array of Rubiaceae in the rain forest understory, and this is the only Agathidinae to appear (but it is accompanied by a diverse array of tachinids, ichneumonids and other braconids). *Desmia*, as a generic concept, has been reared 4,000+ times by the inventory, and yielded 51 Agathidinae; all but this one are in the genus *Alabagrus*.

#### Etymology.

Named in honor of Greg Burton of Mountain View, California, who has enthusiastically supported the conservation of the ACG forest occupied by this parasitoid wasp.

#### Material Examined.

**Holotype**: ♀H6408 (DHJPAR0036705) Costa Rica: Alajuela: Area de Conservación Guanacaste: Sector Rincon Rain Forest: Camino Albergue Oscar: 27.viii.2009, 10.8774N, 85.3236W, 560m [AEI].

**Paratypes** [AEI, HIC, INBio]: Costa Rica: Alajuela: Area de Conservación Guanacaste: Sector Cacao: Cerro Pedregal: 10.928N, 85.475W, 1080m: ♀: 4–11.vii.2009: H2408, H2407; H2490 30.v.-6.vi.2009; H4131 13–20.vi.2009; H2493 23–30.v.2009. Puntarenas: Las Tablas: 30.viii.-5.ix.1995, 8.955N, 83.12W, 1530m:♀: H6795, H7034, H7032, H7067, H7068, H7066, H7031. Embalse: 17.vi.-13.viii.1996, 1300m: ♀: H6421, H7037. Finca Marcos Morales: 5.vii.-25.viii.1995, 1200m: ♀: H7039, H7033, H6414.

### Lytopylus jessicadimauroae

Sharkey sp. n.

urn:lsid:zoobank.org:act:1B8045DE-2662-44D1-BDC5-19F131789A69

http://species-id.net/wiki/Lytopylus_jessicadimauroae

Figs 12 13

#### Description.

**Figure 12.**
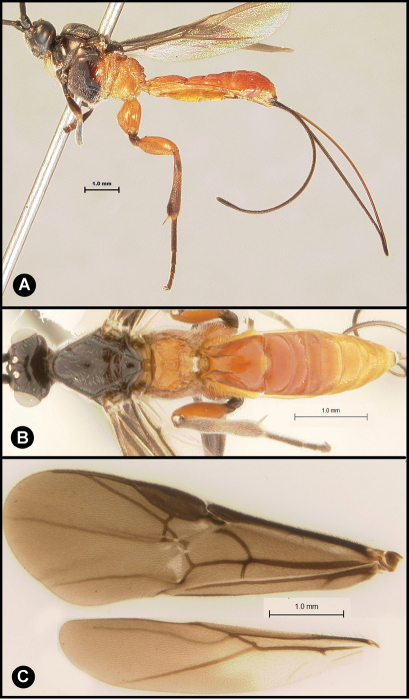
*Lytopylus jessicadimauroae* sp. n. Holotype **a** lateral habitus **b** dorsal habitus **c** wings.

**Figure 13.**
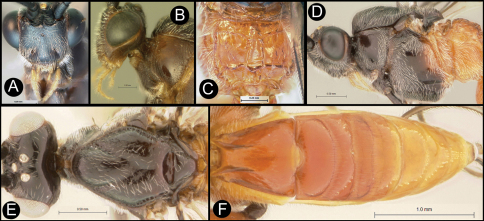
*Lytopylus jessicadimauroae* sp. n. Holotype **a** anterior head **b** lateral head **c** dorsal propodeum **d** lateral head and mesosoma **e** dorsal head and mesosoma **f** dorsal metasoma.

Body length 6.0 – 6.3 mm. Ovipositor length 5.2 – 5.4 mm. Gena acute posterolaterally. Longitudinal groove on interantennal prominence absent. Protuberances on occiput absent. Propodeum with carinae forming areolae, median areola not rounded anteriorly. Notauli well-impressed, smooth without crenulae, or with one or two crenulae restricted to extreme anterior apex along border of mesoscutum. Posterior margin of syntergum 2+3 straight. Median syntergite 2 + 3 mostly smooth with longitudinal striae in the transverse grooves, and striae over much of the third lobe. Forewing mostly or entirely infuscate. Color as in Figs 12, 13. Color variation: The three paratypes differ in having more pale coloration on the thorax than does the holotype, although none is completely pale.

#### Distribution.

Guanacaste Province, Costa Rica. Click here for a distribution map.

#### Biology.

All four rearings are from elachJanzen01 Janzen241, a common leaf-rolling Elachistidae (Stenomatinae) that attacks only *Casearia arguta* (Salicaceae - formerly, Flacourtiaceae) in ACG dry forest. The host caterpillar very distinctively rolls the leaf from the tip to the base instead of from side to side. The tubular cavity in the roll is full of silk and fecal pellets. The wasp cocoon is spun inside the leaf roll. While this caterpillar is abundant, the caterpillars from only about 50 leaf rolls were reared to obtain these four wasps. No other parasitoids were encountered in these caterpillars. This is the only “deep dry forest” *Lytopylus* encountered by the inventory, as all the others are from rain forest or from the interface between dry forest and rain forest.

#### Etymology.

Named in honor of Jessica Dimauro of Toronto, Ontario, Canada, who has enthusiastically supported the conservation of the ACG forest occupied by this parasitoid wasp.

#### Material examined.

Holotype: ♀ H6611 (DHJPAR0015415) Costa Rica: Guanacaste: Area de Conservación Guanacaste: Sector Santa Rosa, Area Administrativa 11.viii.1999, 10.8376N, 85.6187W, 295m [AEI].

**Paratypes** [AEI]: Costa Rica: Guanacaste: Area de Conservación Guanacaste: Sector Santa Rosa: Area Administrativa:,10.8376N, 85.6187W, 295m: H7076, sex unknown, metasoma largely destroyed by dermestids (DHJPAR0015504) 14.vii.1980. ♀ H6616 (DHJPAR0015505) 12.vii.1980. Bosque San Emilio: 22.x.1987, 10.8438N, 85.6138W, 300m. ♂ H7074 (DHJPAR0015506).

### Lytopylus jessiehillae

Sharkey sp. n.

urn:lsid:zoobank.org:act:5ECF221A-A5D7-4923-AA1E-33BB0E1DCF34

http://species-id.net/wiki/Lytopylus_jessiehillae

Figs 14 15

#### Description.

**Figure 14.**
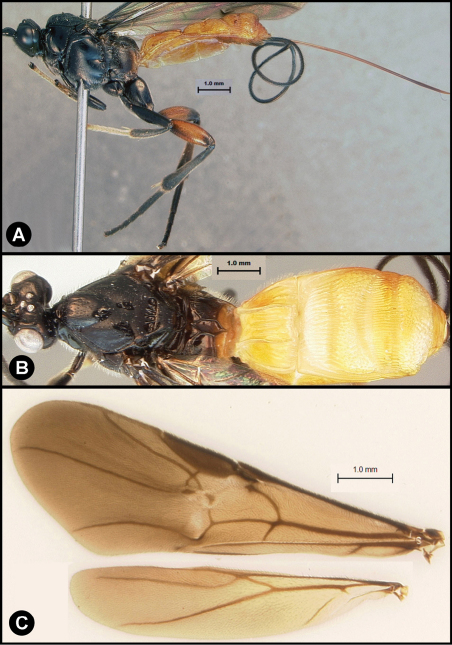
*Lytopylus jessiehillae* sp. n. **a** lateral habitus **b** dorsal habitus **c** wings.

**Figure 15.**
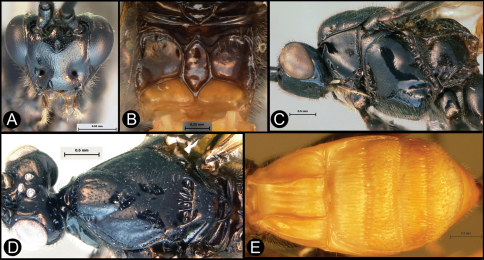
*Lytopylus jessiehillae* sp. n. **a** anterior head **b** dorsal propodeum **c** lateral head and mesosoma **d** dorsal head and mesosoma **e** dorsal metasoma.

Body length 7.0 – 8.3 mm. Ovipositor length 7.2 – 7.7 mm. Gena rounded or with an obtuse angle posterolaterally. Longitudinal groove on interantennal prominence absent. Protuberances on occiput absent. Propodeum with carinae forming areolae, median areola rounded anteriorly. Notauli well-impressed, smooth without crenulae, or with one or two crenulae restricted to extreme anterior apex along border of mesoscutum. Posterior margin of syntergum 2+3 convex, covering most of terminal terga. Median syntergite 2 + 3 longitudinally striate except apical transverse lobe rugose. Forewing mostly or entirely infuscate. Color as in Figs 14, 15. Color variation: Most commonly the mesosoma varies from completely black, to black except for the propodeum and mesothorax which are yellow or orange in whole or in part. Rarely, two specimens, the mesosoma is almost entirely pale. The hind femur varies from entirely melanic to mostly pale. Pale parts of the body vary from yellow to orange.

#### Molecular data.

BOLD process ID/Janzen parasitoid voucher/GenBank accession:

ASAG139-07/DHJPAR0015453/JN034705

ASHYE376-08/DHJPAR0028139/JN034704

ASAG138-07/DHJPAR0015452/JN034704

ASHYF079-09/DHJPAR0028317/JN034702

ASHYE1603-09/DHJPAR0036692/JN034701

ASHYE1631-09/DHJPAR0036720/JN34700

ASHYF080-09/DHJPAR0028318/JN034699

ASHYF019-09/DHJPAR0028257/JN034698

ASHYF078-09/DHJPAR0028316/JN034697

ASHYF017-09/DHJPAR0028255/JN034696

ASHYF018-09/DHJPAR0028256/JN034695

ASHYF081-09/DHJPAR0028319/JN034694

ASHYF068-09/DHJPAR0028306/JN034693

#### Distribution.

Guanacaste Province and Heredia Province, Costa Rica. Click here for a distribution map.

#### Biology.

This wasp is the only species of *Lytopylus* found to date using *Dysodia* (Thyrididae) as its host caterpillar (22 wasp rearings from 3,500+ rearings of about 20 *Dysodia* species). These wasp rearings are almost entirely from three species of rain forest *Dysodia* (89 *Dysodia* Janzen12 feeding exclusively on *Hieronyma oblonga*; 1,241 *Dysodia* Janzen06 feeding on many plant families; 182 *Dysodia* Janzen35 feeding on Annonaceae) in dry forest and dry-rain forest intergrades. Additionally, a single *Lytopylus jessiehillae* has been reared from *Collinsa ferreiceps* (Thyrididae) feeding on the rain forest tree *Sloanea faginea* (Elaeocarpaceae; 73 rearings); the adult of this moth is very similar in body size and wing shape to *Zeuzerodes caenosa* (and very different from *Dysodia*) but the caterpillar is very similar to that of *Dysodia* in body size and leaf roll type. Furthermore, it is the only agathidine reared from the 73 rearings of *Collinsa* on *Sloanea*, and I (DHJ) feel that it is very likely to be an “abnormal” host record.

Only one other agathidine has been reared from the 3,500+ rearings of *Dysodia*, a single specimen of *Amputoearinus niger* from the rain forest *Heisteria concinna* (Olacaceae) being fed on by *Dysodia* Janzen06. *Dysodia* in ACG is, however, also used by at least 7 species of Tachinidae and Microgastrinae braconids.

**Figure 16.**
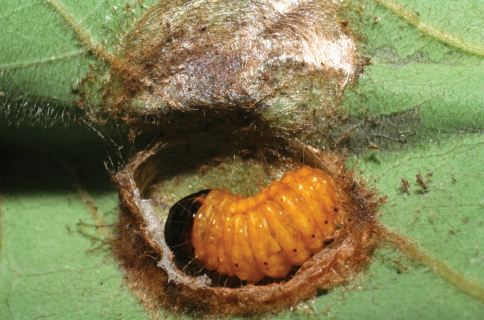
*Lytopylus jessiehillae* prepupal host caterpillar.

**Figure 17.**
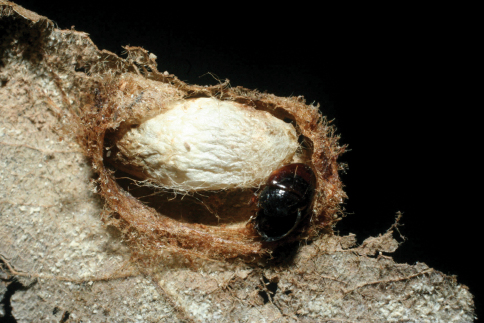
*Lytopylus jessiehillae* cocoon.

*Dysodia* caterpillars construct a close-fitting conical-tubular shelter by cutting a lengthy curved slit in the food plant leaf and rolling that partly detached segment up around its base. The caterpillar then eats the inner parts of the leaf roll. The ovipositing wasp must then penetrate 1–3 layers of leaf and the caterpillar can only move forward and backward to escape. The caterpillar remains in one leaf roll for many days, pushing or ejecting fecal pellets out the “front door”. The distinctive leaf rolls are easily recognized by the researcher, and presumably by a searching wasp as well, but many are empty because the caterpillar has moved on or been taken by a predator. The prepupal caterpillar (Fig. 16) spins a tough silk cocoon between two overlapping leaves (or in the leaf roll). At this time the wasp larva feeds heavily and within 2–3 days has consumed the caterpillar innards and ruptures the caterpillar cuticle to emerge into the pupal chamber and spin a tough white cocoon with the caterpillar head capsule and remaining pelt scrunched onto one end (Fig. 17).

#### Etymology.

Named in honor of Jessie Hill of Hawaii, who has enthusiastically supported the conservation and biodiversity inventory of the ACG forest occupied by this parasitoid wasp.

#### Material examined.

**Holotype**: ♀, H7093 (DHJPAR0028316 – this is a short sequence ) Costa Rica: Guanacaste: Area de Conservación Guanacaste: Sector Pitilla: Loaiciga, 7.ix.2008, 11.019N, 85.4134W, 445m [AEI].

**Paratypes** [AEI, HIC, INBio]: Costa Rica: Guanacaste: Area de Conservación Guanacaste:Sector Pitilla: Loaiciga, 11.019N, 85.4134W, 445m: ♀ H7090 (DHJPAR0028305) 7.ix.2008, ♀ H7091 (DHJPAR0028257) 5.ix.2008, ♀ H7098 (DHJPAR0028319) 4.ix.2008, ♀ H7063 (DHJPAR0028306) 4.ix.2008, ♀ H7083 (DHJPAR0028309) 9.iv.2008. ♂ H7085 (DHJPAR0028313) 6.ix.2008, ♂ H7096 (DHJPAR0028255) 4.ix.2008, ♂ H7089 (DHJPAR0028256) 28.viii.2008. Sendero Cuestona, 10.9945N, 85.4146W, 640m: ♀ H8398 (DHJPAR0015455) 9.iv.2004, ♂ H8399 (DHJPAR0015454) 7.iv.2004. Sector Cacao: Sendero Toma Agua, 10.928N, 85.4668W, 1140m: ♀ 4.iv.2001: H7088 (DHJPAR0015453), ♀ H7086 (DHJPAR0015452), ♂ H8396 (DHJPAR0015451). Sector Del Oro: Guacimos, 11.014N, 85.4749W, 380m: ♂ H7087 (DHJPAR0036692) 3.viii.2009. Sector Mundo Nuevo: Vado Ocotea, 10.7638N, 85.3784W, 565m: ♀ H7095 (DHJPAR0028139) 30.vii.2008. Vado Zanja Tapada, 10.7648N, 85.3845W, 550m: ♀ H7094 (DHJPAR0040216) 4.vii.2010, ♀ H7099 (DHJPAR0040224) 3.vii.2010. Porton Rivas, 10.7586N, 85.3727W, 570m: ♀ H6410 (DHJPAR0028318) 9.vii.2008, ♂ H7092 (DHJPAR0028317) 6.vii.2008. Punta Plancha, 10.7416N, 85.4273W, 420m: ♀ H7097 (DHJPAR0040226) 1.vii.2010. Area de Conservación Guanacaste: Sector Rincon Rain Forest: Camino Albergue Oscar, 10.8774N, 85.3236W, 560m: ♀ H7084 (DHJPAR0036720) 1.ix.2009.

Costa Rica: Heredia: Est. Biol. La Selva, 10.433N, 84.017W, 150m: ♀ H8397 vii.1993.

### Lytopylus macadamiae

(Briceño and Sharkey) comb. n.

http://species-id.net/wiki/Lytopylus_macadamiae

Fig. 18 19

Bassus macadamiae Briceño and Sharkey, 2000.

#### Description.

**Figure 18.**
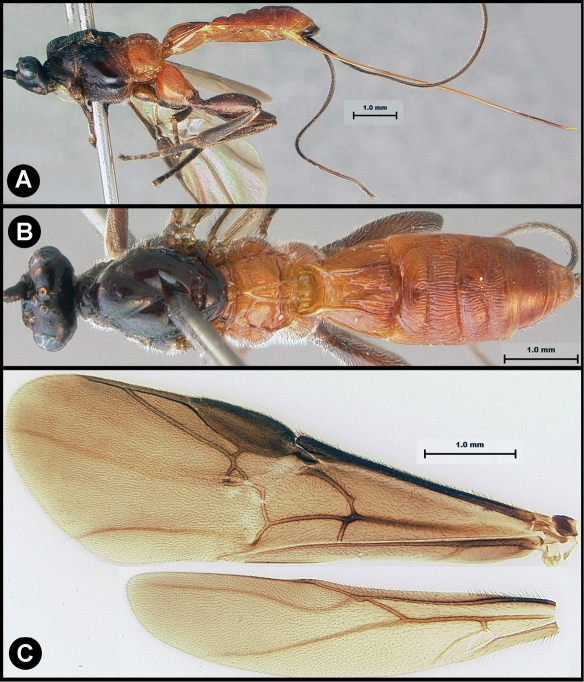
*Lytopylus macadamiae* (Briceno and Sharkey) **a** lateral habitus **b** dorsal habitus **c** wings.

**Figure 19.**
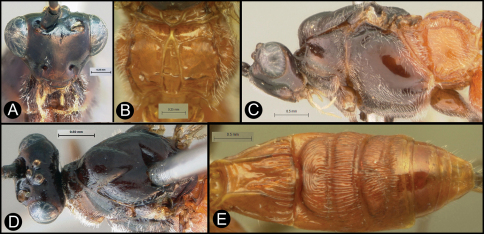
*Lytopylus macadamiae* (Briceno and Sharkey) **a** anterior head **b** dorsal propodeum **c** lateral head and mesosoma **d** dorsal head and mesosoma **e** dorsal metasoma.

Body length 4.4 – 6.8 mm. Ovipositor length 4.7 – 7.1. Gena rounded or with an obtuse angle posterolaterally. Longitudinal groove on interantennal prominence absent. Protuberances on occiput absent. Propodeum with carinae forming areolae, median areola not rounded anteriorly. Notauli well-impressed, smooth without crenulae, or with one or two crenulae restricted to extreme anterior apex along border of mesoscutum. Posterior margin of syntergum 2+3 straight. Median syntergite 2 + 3 longitudinally striate except extreme apex and medial transverse groove smooth. Forewing mostly or entirely infuscate. Color as in Figs 18, 19. Color variation: There is very little variation in color.

#### Distribution.

Known from Venezuela and Costa Rica. For a distribution map, click here.

#### Biology.

Reared from*Ecdytolopha aurantiana* and *Ecedytolopha tortricornis***_,_** (Tortricidae), both on *Macadamia integrifolia* (see Briceño and Sharkey 2000).

#### Material examined.

See Briceño and Sharkey, 2000.

### Lytopylus mingfangi

Sharkey sp. n.

urn:lsid:zoobank.org:act:3A35BACA-FDC2-4A1C-B4CF-F058004F07D6

http://species-id.net/wiki/Lytopylus_mingfangi

Figs 20 21

#### Description.

**Figure 20.**
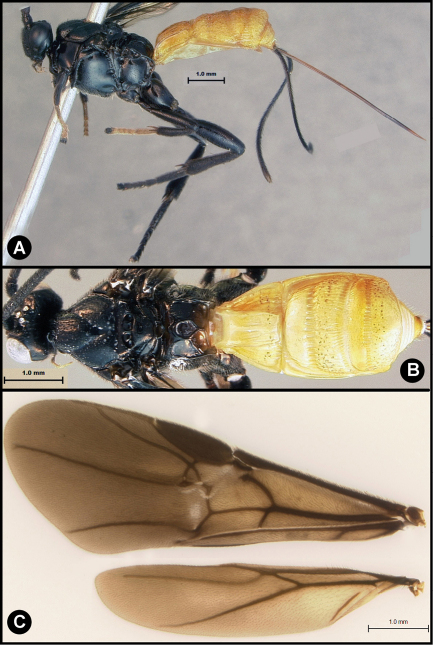
*Lytopylus mingfangi* sp. n. **a** lateral habitus **b** dorsal habitus **c** wings.

**Figure 21.**
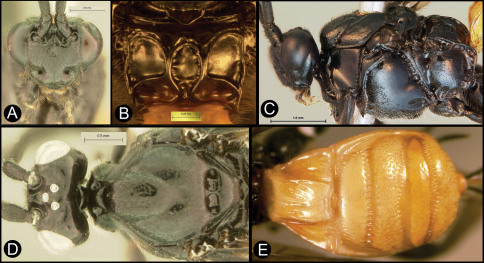
*Lytopylus mingfangi* sp. n. **a** anterior head **b** dorsal propodeum **c** lateral head and mesosoma **d** dorsal head and mesosoma **e** dorsal metasoma.

Body length 6.5 – 6.9 mm. Ovipositor length 5.3 – 5.6 mm. Gena rounded or with an obtuse angle posterolaterally. Longitudinal groove on interantennal prominence absent. Protuberances on occiput absent. Propodeum with carinae forming areolae, median areola rounded anteriorly. Notauli well-impressed, smooth without crenulae, or with one or two crenulae restricted to extreme anterior apex along border of mesoscutum. Posterior margin of syntergum 2+3 convex, covering most of terminal terga. Median syntergite 2 + 3 striate between lobes, first lobe usually smooth but varies from smooth to weakly rugose; second lobe varies from smooth to weakly rugose to striate; third lobe always smooth medially; fourth lobe varies from weakly striate to weakly rugose to smooth. Forewing mostly or entirely infuscate. Color as in Figs 20, 21. Color variation: Quite consistent in color. The propodeum may be pale posteriorly. The pale color of the abdomen is almost always yellow but it is orange in a few specimens.

#### Molecular data.

BOLD process ID/Janzen parasitoid voucher/GenBank accession:

ASBC962-07/DHJPAR0021150/JN034721

ASHYC4672-10/DHJPAR0037927/JN034720

ASHYM043-08/DHJPAR0023291/JN034719

ASAG218-07/DHJPAR0015532/JN034718

ASHYD1531-09/DHJPAR0036340/JN034717

ASHYF030-09/DHJPAR0028268/JN034716

ASHYD1533-09/DHJPAR0036342/JN034715

ASHYD1534-09/DHJPAR0036343/JN034714

ASHYE1634-09/DHJPAR0036723/JN034713

ASHYE268-08/DHJPAR0028031/JN034712

ASHYD2379-10/DHJPAR0038806/JN034711

ASBC979-07/DHJPAR0021167/JN034721

ASHYC4673-10/DHJPAR0037928/JN034709

ASHYC4672-10/DHJPAR0037927/JN034720

#### Distribution.

Alajuela Province, Costa Rica. Click here for a distribution map.

#### Biology.

This wasp is the only species of *Lytopylus* found to date using *Zeuzerodes caenosa* (Thyrididae) as its host caterpillar (26 wasp rearings from 193 rearings of wild caught *Zeuzerodes caenosa* caterpillars), and apparently uses only this caterpillar. These parasitoid rearings are from the small rain forest trees *Mortoniodendron costaricense* (Tiliaceae, n =16) and *Quararibea funebris* (Bombacaceae, n = 10). Although the DNA barcode cluster contains significant divergence (max K2P 4.98%, average 1.216%), there is no morphological or ecological indication that these host-tree records suggest a wasp species complex, but rather, that the wasp finds *Zeuzerodes caenosa* wherever it is.

**Figure 22.**
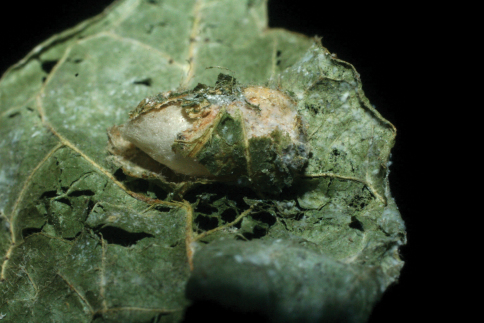
*Lytopylus mingfangi* cocoon.

*Zeuzerodes caenosa* caterpillars construct a leaf roll shelter as described for *Dysodia*, but it is more sloppy and loose-fitting to the caterpillar, seemingly with more room to move around in (and perhaps in which to flee from a wasp ovipositor). The wasp cocoon is spun inside the caterpillar prepupal chamber (Fig. 22) but usually kills the prepupal caterpillar before it has had the occasion to spin a full (and very tough) cocoon.

In ACG rain forest, *Zeuzerodes caenosa* is used only by *Lytopylus mingfangi*, with the exception of a single unidentified tachinid fly parasitoid record.

Of all 5,700+ rearings of ACG Thyrididae caterpillars, only these two *Lytopylus* species appear to be regular users of the thryridid caterpillar fauna. The single rearing of *Austroearinus niger* and of *Therophilus* Sharkey01 from Thyrididae suggest that these two species of wasps normally use other species of host caterpillars, leaving *Lytopylus mingfangi* and *Lytopylus jessiehillae* as the two agathidine parasitoids of the ACG thyridids known to date.

#### Etymology.

Named in honor of Ming Fang of Hawaii and China, who has enthusiastically supported the conservation of the ACG forest occupied by this parasitoid wasp.

#### Material examined.

**Holotype:** ♀, H8016 (DHJPAR0040338), Costa Rica: Guanacaste: Area de Conservación Guanacaste: Sector San Cristobal: Tajo Angeles, 7.viii.2010, 10.86472N, 85.4153W, 540m [AEI].

**Paratypes** [AEI, HIC, INBio]: Costa Rica: Guanacaste and Alajuela: Area de Conservación Guanacaste: Sector San Cristobal: Rio Blanco Abajo, 10.90037N, 85.3725W, 500m: ♀H7055 (DHJPAR0021150) 18.vii.2007. ♂ H7062 (DHJPAR0021167) 5.viii.2007. San Gabriel, 10.87766N, 85.3934W, 645m: ♀ H6416 (DHJPAR0023291) 9.vii.2007. ♀ H7059 (DHJPAR0028031) 3.vii.2008. ♀ H7058 (DHJPAR0037927) 10.xi.2009. ♂ H7057 (DHJPAR0038806) 11.iii.2010. ♂ H7061 (DHJPAR0037928) 12.xi.2009. Sendero Carmona, 10.87621N, 85.3863W, 670m: ♀ H7060 (DHJPAR0028268) 28.viii.2008. Sendero Colegio, 10.89296N/85.3788. 520m: ♀ H7064 (DHJPAR0015532) 31.x.2005. Sendero Corredor, 10.87868N, 85.3896W, 620m.♀ H7054 (DHJPAR0036723) 22.viii.2009. Tajo Angeles, 10.86472N, 85.4153W, 540m: ♀ H6626 (DHJPAR0036342) 8.viii.2009. ♀ H7961 (DHJPAR0041593) 25.x.2010. ♀ H7052 (DHJPAR0036344) 9.viii.2009. ♀ H7905 (DHJPAR0041599) 13.viii.2010. ♀ H8014 (DHJPAR0040346) 4.viii.2010. ♀ H7065 (DHJPAR0036340) 7.viii.2009. ♂ H7051 (DHJPAR0038907) 28.viii.2010. H7053 (DHJPAR0036343) 7.viii.2009. H7955 (DHJPAR0041595) 11.x.2010. ♂ H7952 (DHJPAR0041590) 25.x.2010. Sector Rincon Rain Forest: Sendero Albergue Crater, 10.84866N, 85.3281W, 980m: ♀ H7056 (DHJPAR0037916) 6.xi.2009.

### Lytopylus rebeccashapleyae

Sharkey sp. n.

urn:lsid:zoobank.org:act:8311E1B3-19D9-4D87-ABAF-15E4971D5721

http://species-id.net/wiki/Lytopylus_rebeccashapleyae

Figs 23 24

#### Description.

**Figure 23.**
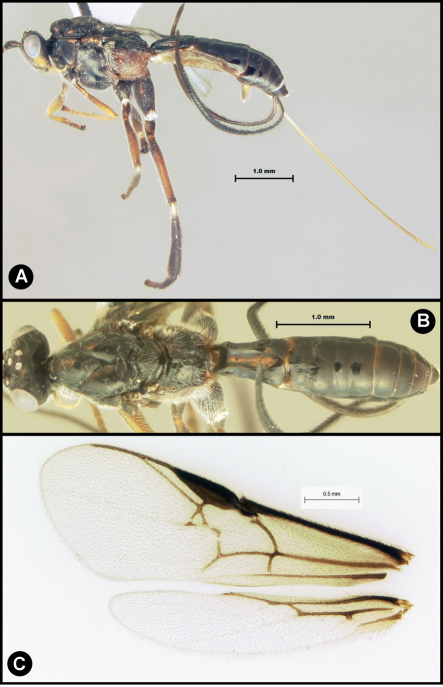
*Lytopylus rebeccashapleyae* sp. n. **a** lateral habitus **b** dorsal habitus **c** wings.

**Figure 24.**
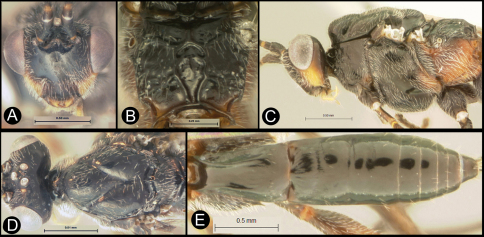
*Lytopylus rebeccashapleyae* sp. n. **a** anterior head **b** dorsal propodeum **c** lateral head and mesosoma **d** dorsal head and mesosoma **e** dorsal metasoma.

Body length 4.2 – 4.9 mm. Ovipositor length 4.2 – 4.5 mm. Gena acute posterolaterally. Longitudinal groove on interantennal prominence absent. Protuberances on occiput absent. Propodeum with carinae forming areolae, median areola rounded anteriorly, or with carinae forming areolae, median areola not rounded anteriorly. Notauli well-impressed, smooth without crenulae, or with one or two crenulae restricted to extreme anterior apex along border of mesoscutum. Posterior margin of syntergum 2+3 straight. Median syntergite 2 + 3 mostly smooth except for weak striae anteromedially. Forewing clear except base slightly infuscate. Color as in Figs 23, 24. Color variation: Body color is consistent across the five specimens examined.

#### Molecular data.

BOLD process ID/Janzen parasitoid voucher/GenBank accession:

ASHYB1809-10/DHJPAR0039521/JN034725

ASHYB1810-10/DHJPAR0039522/JN034724

ASHYB1794-10/DHJPAR0039506/JN034723

ASHYB1813-10/DHJPAR0039525/JN024722

#### Distribution.

Alajuela Province and Puntarenas Province, Costa Rica. Click here for a distribution map.

#### Biology.

All five rearings are from *Episimu*s Brown002 (Tortricidae, Olethreutinae), a relatively common solitary leaf webber feeding on the shrubs *Vismia baccifera* and *Vismia billbergiana* (Clusiaceae) that occur throughout disturbed ACG rain forest sites (150+ caterpillar rearings). However, all five wasp rearings are from a batch of *Episimus* Brown002 larvae collected in late April and early May in just one intermediate elevation site on the very wet Caribbean slopes of Volcan Rincon de la Vieja in Sector Rincon Rain Forest.

#### Etymology.

*Lytopylus rebeccashapleyae* is named in honor of Rebecca Shapley of Mountain View, California, who has enthusiastically supported the conservation of the ACG forest occupied by this parasitoid wasp.

#### Material examined.

**Holotype**: ♀, H8047 (DHJPAR0039525) Costa Rica: Alajuela: Area de Conservación Guanacaste: Sector Rincon Rain Forest: Sendero al Crater: 19.v.2010, 10.8488N, 85.3281W, 980m. [AEI].

**Paratypes** [AEI, HIC, INBio]: Costa Rica: Alajuela: Area de Conservación Guanacaste: Sector Rincon Rain Forest: Camino Albergue Oscar: 10.8774N, 85.3236W, 560m: 17.v.2010: ♀ H8020 (DHJPAR0039521), ♀ H7959 (DHJPAR0039506); ♀ H7960 (DHJPAR0039507) 14.v.2010. ♀ H8032 (DHJPAR0039522) 8.v.2010.

### Lytopylus robpringlei

Sharkey sp. n.

urn:lsid:zoobank.org:act:D23EE5BE-6C35-4348-B9F3-E8E815FC0868

http://species-id.net/wiki/Lytopylus_robpringlei

Figs 25 26

#### Description.

**Figure 25.**
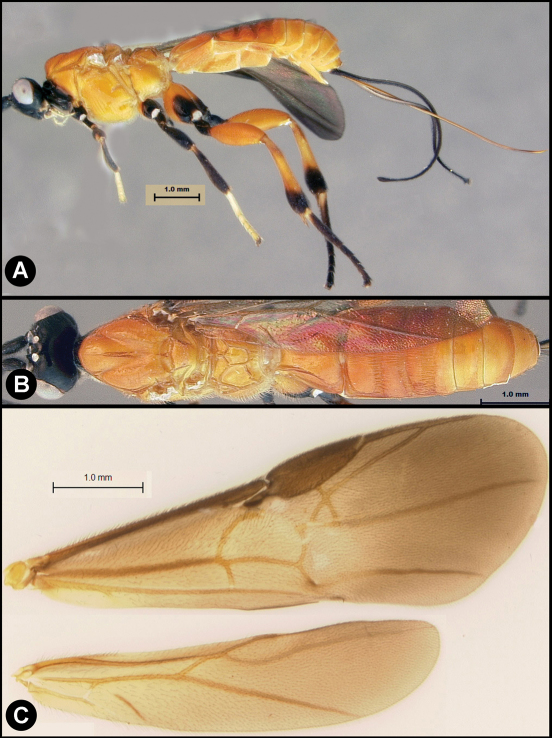
*Lytopylus robpringlei* sp. n. Holotype **a** lateral habitus **b** dorsal habitus **c** wings.

**Figure 26.**
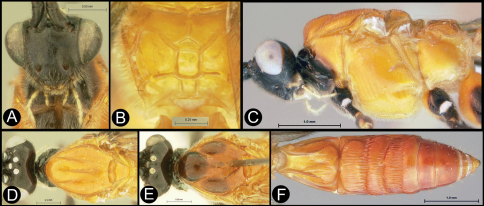
*Lytopylus robpringlei* sp. n. Holotype **a** anterior head **b** dorsal propodeum **c** lateral head and mesosoma **d** dorsal head and mesosoma **e** dorsal head and mesosoma variation **f** dorsal metasoma.

Body length 7.5 mm sole female (5.8 – 7.2 males). Ovipositor length 5.8 mm. Gena rounded or with an obtuse angle posterolaterally. Longitudinal groove on interantennal prominence present. Protuberances on occiput absent. Propodeum with carinae forming areolae, median areola not rounded anteriorly. Notauli well-impressed, smooth without crenulae, or with one or two crenulae restricted to extreme anterior apex along border of mesoscutum. Posterior margin of syntergum 2+3 straight. Median syntergite 2 + 3 varying from entirely striate except for smooth apex to smooth with longitudinal striae restricted to the transverse grooves. Forewing mostly or entirely infuscate. Color as in Figs 25, 26. Color variation: Mesoscutal lobes slightly melanic in one male specimen otherwise color consistent across the four examined specimens.

#### Molecular data.

BOLD process ID/Janzen parasitoid voucher/GenBank accession:

ASHYB1814-10/DHJPAR0039526/JN034728

ASHYE1114-09/DHJPAR0035292/JN034727

#### Distribution.

Alajuela Province, Costa Rica.Click here for a distribution map.

#### Biology.

All four rearings are from Elachistidae (Stenomatinae) leaf tiers feeding on rain forest Sapotaceae: 3 from *Chlamydastis rhomaeopa* on *Chrysophyllum brenesii*, and 1 elachJanzen01 Janzen35 from *Pouteria* 17499. These leaf tiers construct a somewhat tubular channel of silk and a few fecal pellets between two overlapping leaves that are quite tightly silked together, and then surface skeletonize the leaf and feed at the leaf edges. The ovipositing wasp would need to penetrate through the leaf from above or below, skewering the caterpillar in the process. There is no externally visible indication that there is a wasp egg or larva inside the wild-collected caterpillar. At about the time the caterpillar is prepupal (which tends to be at least 5–10 days after the caterpillar is brought in from the wild), the wasp larva feeds strongly enough to immobilize the caterpillar, and 1–2 days later emerges through the body wall to spin a white cocoon in the caterpillar pupal chamber that is a space between the two leaves silked together and in a loosely defined tubular nest of silk and fecal pellets. The cranium and pelt fragments of the caterpillar are bunched together at one end of the wasp cocoon. The pupal period of the wasp is 12–20 days.

The elachistid host caterpillars feeding on Sapotaceae are parasitized by a variety of tachinid flies, ichneumonid wasps and microgastrine braconid wasps, but (at least with this small number of rearing records) appear not to be also attacked by other agathidine wasps.

#### Etymology.

*Lytopylus robpringlei* is named in honor of Rob Pringle of Princeton, New Jersey, who has enthusiastically supported the conservation of the ACG forest occupied by this parasitoid wasp.

#### Material examined.

**Holotype**: ♀, H8019 (DHJPAR0039526) Costa Rica: Alajuela: Area de Conservación Guanacaste: Sector San Cristobal: Tajo Angeles: 11.vi.2010, 10.865N, 85.415W, 540m [AEI].

**Paratypes** [AEI, HIC, INBio]: Costa Rica: Alajuela: Area de Conservación Guanacaste: Sector San Cristobal: Tajo Angeles, 10.8647N, 85.4153W, 540m: ♂ H7814 (DHJPAR0041557) 8.i.2011. ♂ H6635 (DHJPAR0035292) 14.v.2009. Rio Blanco Abajo, 10.9003N, 85.3725W, 500m: ♂ H6435 (DHJPAR0015456) 14.iv.2003.

### Lytopylus sandraberriosae

Sharkey sp. n.

urn:lsid:zoobank.org:act:826936F6-CEDA-46CB-AB53-D081E7F3A7CE

http://species-id.net/wiki/Lytopylus_sandraberriosae

Figs 27 28

#### Description.

**Figure 27.**
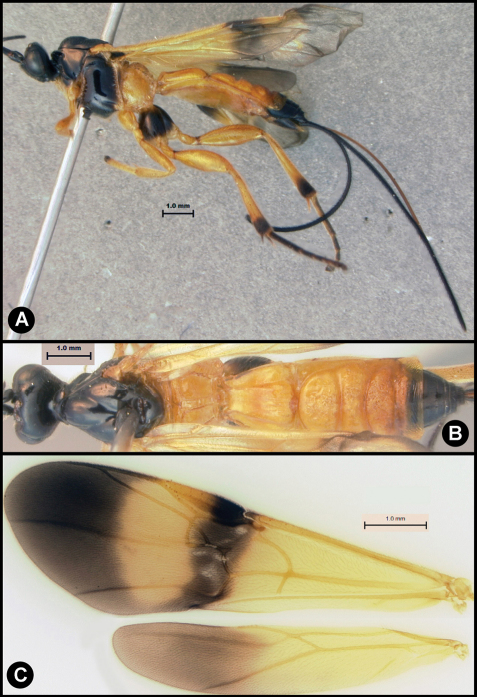
*Lytopylus sandraberriosae* sp. n. Holotype **a** lateral habitus **b** dorsal habitus **c** wings.

**Figure 28.**
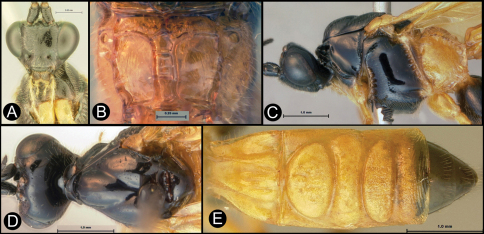
*Lytopylus sandraberriosae* sp. n. Holotype **a** anterior head **b** dorsal propodeum **c** lateral head and mesosoma **d** dorsal head and mesosoma **e** dorsal metasoma.

Body length 8.9 mm (sole female) 7.9 – 8.2 (2 males). Ovipositor length 8.9 mm. Gena rounded or with an obtuse angle posterolaterally. Longitudinal groove on interantennal prominence absent. Protuberances on occiput absent. Propodeum with carinae forming areolae, median areola rounded anteriorly, or with carinae forming areolae, median areola not rounded anteriorly. Notauli well-impressed, smooth without crenulae, or with one or two crenulae restricted to extreme anterior apex along border of mesoscutum. Posterior margin of syntergum 2+3 straight. Median syntergite 2 + 3 mostly weakly rugose with fine transverse striae apically. Forewing banded yellow and infuscate. Color as in Figs 27, 28. Color variation: There is little color variation across the three specimens examined.

#### Molecular data.

BOLD process ID/ Janzen parasitoid voucher/GenBank accession:

ASBC960-07/DHJPAR0021148/JN034729.

#### Distribution.

Guanacaste province, Costa Rica.Click here for a distribution map.

#### Biology.

All three rearings are from Pyralidae (Epipaschiinae) gregarious leaf webbers (*Deuterollyta oediperalis*) that live in conspicuous groups of 2–50 sib larvae moving freely through the silk and leaf mass in the crowns of rain forest understory Lauraceae (in this case, *Nectandra hihua* and an unidentified species of Lauraceae). Oviposition into one of the unrestrained caterpillars in the silk and leaf tangle will be a quite different challenge from oviposition into a single larva in its nest between two silked-together leaves (as is the case with other *Lytopylus*). Only DHJPAR0021148 has successfully barcoded, so the grouping of these three individuals as one species is based solely on their morphology. Their host records give credence to this grouping.

**Figure 29.**
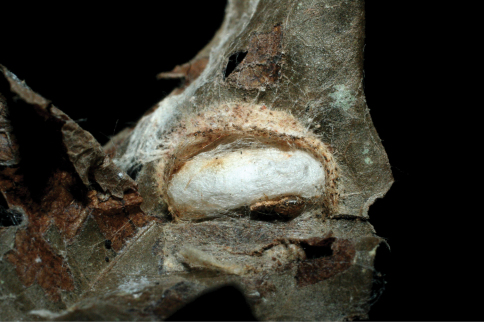
*Lytopylus sandraberriosae* cocoon.

The wasp cocoon is spun in the caterpillar’s prepupal chamber on the leaf surface (Fig. 29). The inventory has reared 5,422 epipaschiine pyralids from various food plants in ACG rain forest and dry forest (about half in the Lauraceae) during 33 years, to yield these three *Lytopylus* specimens (plus one *Alabagrus* and two *Austroearinus*).

*Lytopylus sandraberriosae* is a strange agathidine morphologically, biologically, and in COI composition. When we included another outgroup, a species of *Alabagrus*, in parsimony and NJ analyses (not shown), *Lytopylus sandraberriosae* did not nest with the other *Lytopylus*. Rather, *Braunsia* was positioned as the sister to the remaining *Lytopylus*. The species could well represent a new genus, but more molecular data is needed to confirm this suspicion.

#### Etymology.

Named in honor of Sandra Berrios Torres of Atlanta, Georgia, who has enthusiastically supported the conservation of the ACG forest occupied by this parasitoid wasp.

#### Material examined.

**Holotype**: ♀, H6432 (DHJPAR0040220) Costa Rica: Alajuela: Area de Conservación Guanacaste: Sector Rincon Rain Forest: Conguera, 2.vii.2010, 10.9159N, 85.2663W, 420m [AEI].

**Paratypes** [AEI, HIC, INBio]: Costa Rica: Guanacaste: Area de Conservación Guanacaste: Sector Rincon Rain Forest: Conguera, 10.9159N, 85.2663W, 420m: ♂ H6428 (DHJPAR0040219) 4.vii.2010. Camino Rio Francia, 10.9042N, 85.2865W, 410m: ♂ H6628 (DHJPAR0021148) 7.vii.2007.

### Lytopylus vaughntani

Sharkey sp. n.

urn:lsid:zoobank.org:act:9BF81C64-B4BB-4485-9BAF-5235847A2D46

http://species-id.net/wiki/Lytopylus_vaughntani

Figs 30 31

#### Description.

**Figure 30.**
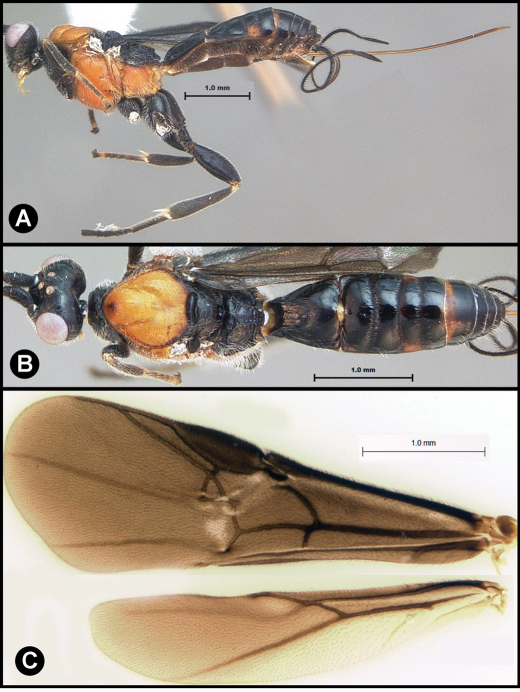
*Lytopylus vaughntani* sp. n. Holotype **a** lateral habitus **b** dorsal habitus **c** wings.

**Figure 31.**
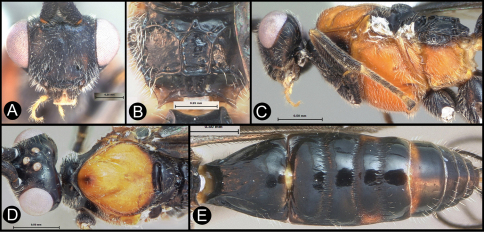
*Lytopylus vaughntani* sp. n. Holotype **a** anterior head **b** dorsal propodeum **c** lateral head and mesosoma **d** dorsal head and mesosoma **e** dorsal metasoma.

Body length 3.9 – 4.8 mm. Ovipositor length 3.1 – 3.8 mm. Gena acute posterolaterally, or rounded or with an obtuse angle posterolaterally. Longitudinal groove on interantennal prominence present. Protuberances on occiput absent. Propodeum with carinae forming areolae, median areola not rounded anteriorly. Notauli well-impressed, smooth without crenulae, or with one or two crenulae restricted to extreme anterior apex along border of mesoscutum. Posterior margin of syntergum 2+3 straight. Median syntergite 2 + 3 mostly smooth with longitudinal striae restricted to the transverse grooves, rarely the third lobe is partially striate. Forewing mostly or entirely infuscate. Color as in Figs 30, 31. Color variation: Color is quite consistent across the six specimens examined. Some specimens are slightly paler than that shown in figures 30 and 31 especially on the metasoma.

#### Molecular data.

BOLD process ID/ Janzen parasitoid voucher/GenBank accession:

ASHYB1349-09/DHJPAR0030608/JN034730.

#### Distribution.

Heredia and Alajuela provinces, Costa Rica. Click here for a distribution map.

#### Biology.

The single rearing is from an unidentified elachistid (Elachistidae, Stenomatinae) leaf roller/webber on the rain forest herbaceous composite *Fleischmannia pycnocephala* (Asteraceae).  While 52 rearing records from this plant have yielded a diverse array of Crambidae, Thyrididae, Tortricidae and Elachistidae parasitized by a diverse array of ichneumonids, tachinids and microgastrine braconids, this is the only Agathidinae encountered to date.

#### Etymology.

Named in honor of Vaughn Tan of Cambridge, Massachusetts, who has enthusiastically supported the conservation of the ACG forest occupied by this parasitoid wasp.

#### Material examined.

**Holotype**: ♀H6614 (DHJPAR0030608) Costa Rica: Alajuela: Area de Conservación Guanacaste: Sector San Cristobal: Puente Palma: 13.ii.2009, 10.916N, 85.379W, 460m [AEI].

**Paratypes** [AEI, HIC, INBio]: Costa Rica: Heredia: Est. Bio. La Selva: 10.433N, 84.017W, 150m: ♀H7080 vii.1998, ♂H7079 xii.1999. Sector Cocori: Limon: 1.1995, 10.365N, 83.734W, 100m: ♀: H6467, H7075, ♂H7077.

## Supplementary Material

XML Treatment for Lytopylus bradzlotnicki

XML Treatment for Lytopylus colleenhitchcockae

XML Treatment for Lytopylus flavicalcar

XML Treatment for Lytopylus gregburtoni

XML Treatment for Lytopylus jessicadimauroae

XML Treatment for Lytopylus jessiehillae

XML Treatment for Lytopylus macadamiae

XML Treatment for Lytopylus mingfangi

XML Treatment for Lytopylus rebeccashapleyae

XML Treatment for Lytopylus robpringlei

XML Treatment for Lytopylus sandraberriosae

XML Treatment for Lytopylus vaughntani

## Supplementary materials

Supplementary material 1 DELTA data matrix, images, and other files Data type: various Explanation note: DELTA data matrix, images, and other files to the dichotomous key for Lytopylus (Hymenoptera: Braconidae: Agathidinae) of Costa Rica. (doi: 10.3897/zookeys.130.1569.app1) This dataset is made available under the Open Database License (http://opendatacommons.org/licenses/odbl/1.0/). The Open Database License (ODbL) is a license agreement intended to allow users to freely share, modify, and use this Dataset while maintaining this same freedom for others, provided that the original source and author(s) are credited.Michael J. Sharkey, Stephanie Clutts, Erika M. Tucker, Daniel Janzen, Winnie Hallwachs, Tanya Dapkey, M. Alex Smith

Supplementary material 2 DELTA data matrix, images, and other files Data type: various Explanation note: DELTA data matrix, images, and other files to the dichotomous key for Lytopylus (Hymenoptera: Braconidae: Agathidinae) of Costa Rica. (doi: 10.3897/zookeys.130.1569.app2) This dataset is made available under the Open Database License (http://opendatacommons.org/licenses/odbl/1.0/). The Open Database License (ODbL) is a license agreement intended to allow users to freely share, modify, and use this Dataset while maintaining this same freedom for others, provided that the original source and author(s) are credited.Michael J. Sharkey, Stephanie Clutts, Erika M. Tucker, Daniel Janzen, Winnie Hallwachs, Tanya Dapkey, M. Alex Smith

Supplementary material 3 Interactive key, in IntKey format Data type: Interactive key Explanation note: Interactive key, in IntKey format, to Lytopylus (Hymenoptera: Braconidae: Agathidinae) of Costa Rica. (doi: 10.3897/zookeys.130.1569.app3) This dataset is made available under the Open Database License (http://opendatacommons.org/licenses/odbl/1.0/). The Open Database License (ODbL) is a license agreement intended to allow users to freely share, modify, and use this Dataset while maintaining this same freedom for others, provided that the original source and author(s) are credited.Michael J. Sharkey, Stephanie Clutts, Erika M. Tucker, Daniel Janzen, Winnie Hallwachs, Tanya Dapkey, M. Alex Smith
