# Supplementary figures and images for: Lytopylus Förster (Hymenoptera, Braconidae, Agathidinae) species from Costa Rica, with an emphasis on specimens reared from caterpillars in Area de Conservación Guanacaste
Source: Zookeys. 2011 Sep 24;(130):379–419. doi: 10.3897/zookeys.130.1569 (PMC3252762; doi:10.3897/zookeys.130.1569)

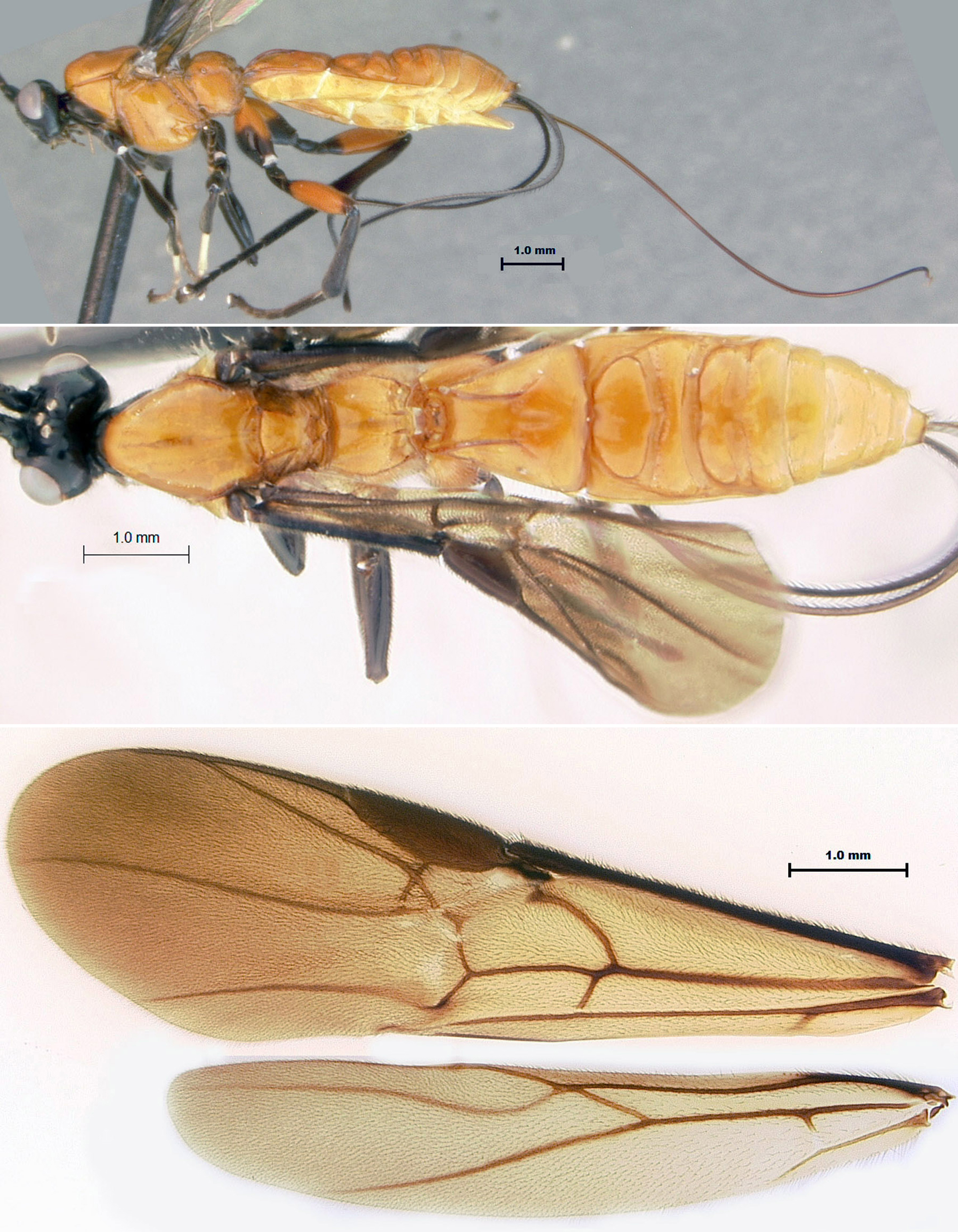

Supplement: Supplementary material 1 — DELTA data matrix, images, and other files [file ZooKeys-130-379-s001.zip › Lytopylus images/flavicalcar1A.jpg]

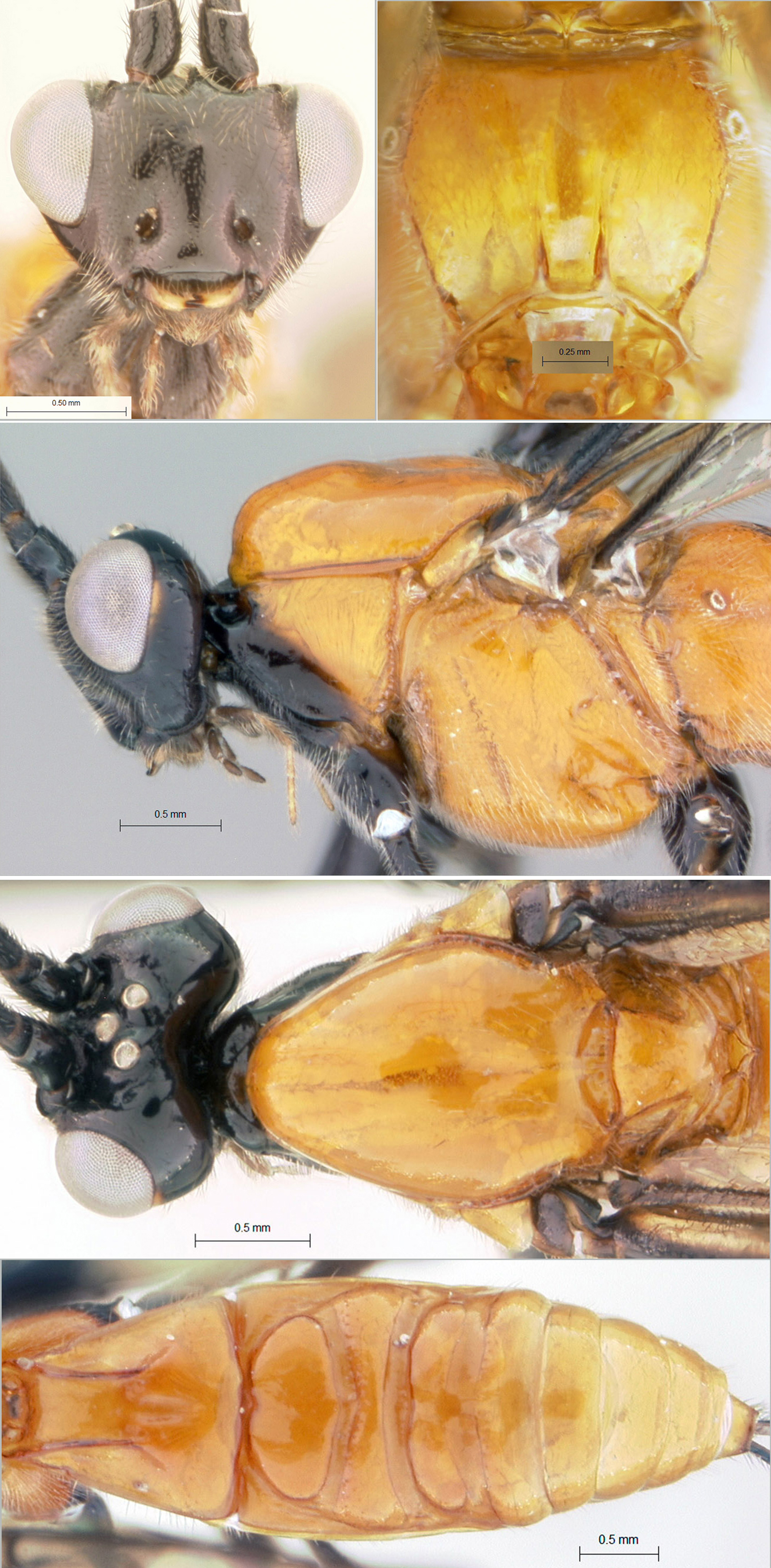

Supplement: Supplementary material 1 — DELTA data matrix, images, and other files [file ZooKeys-130-379-s001.zip › Lytopylus images/flavicalcar2A.jpg]

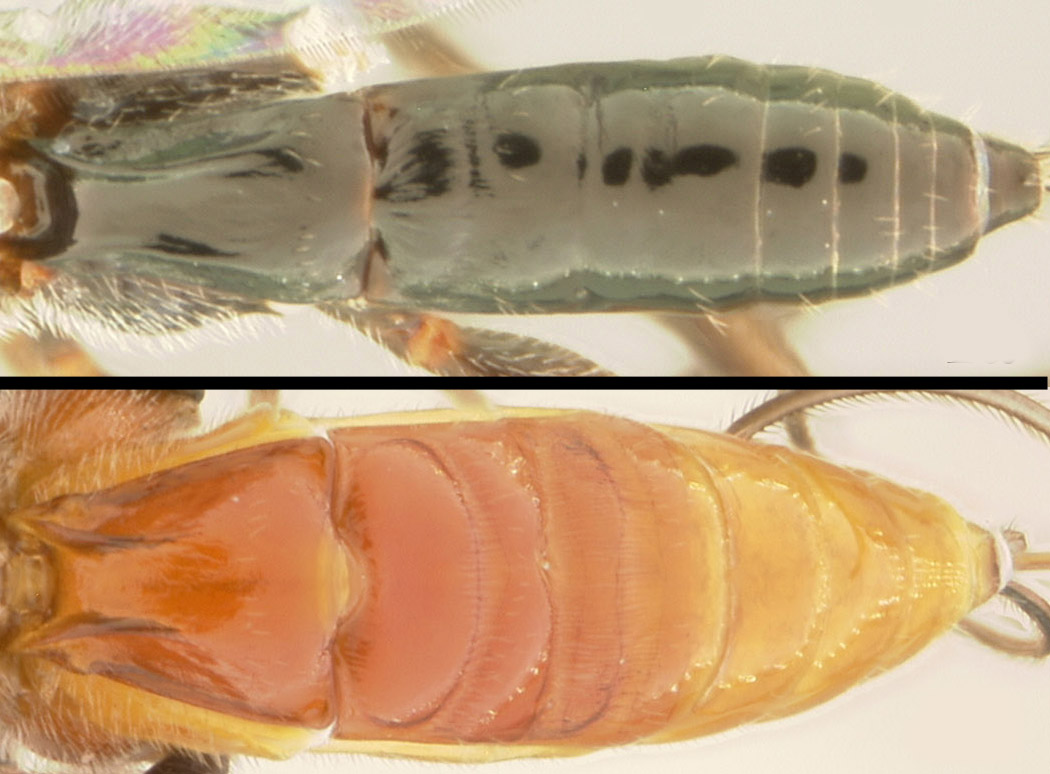

Supplement: Supplementary material 1 — DELTA data matrix, images, and other files [file ZooKeys-130-379-s001.zip › Lytopylus images/key1.jpg]

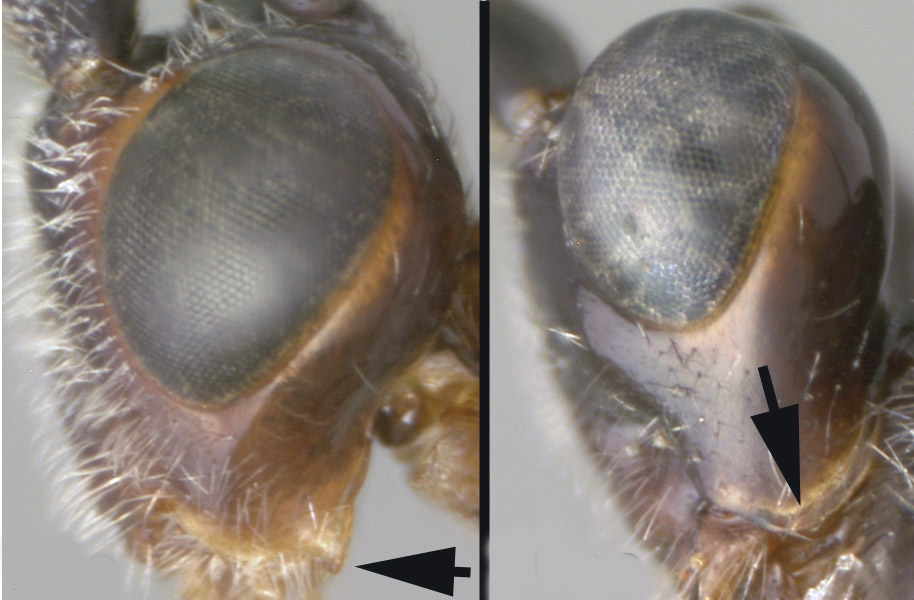

Supplement: Supplementary material 1 — DELTA data matrix, images, and other files [file ZooKeys-130-379-s001.zip › Lytopylus images/key10a.jpg]

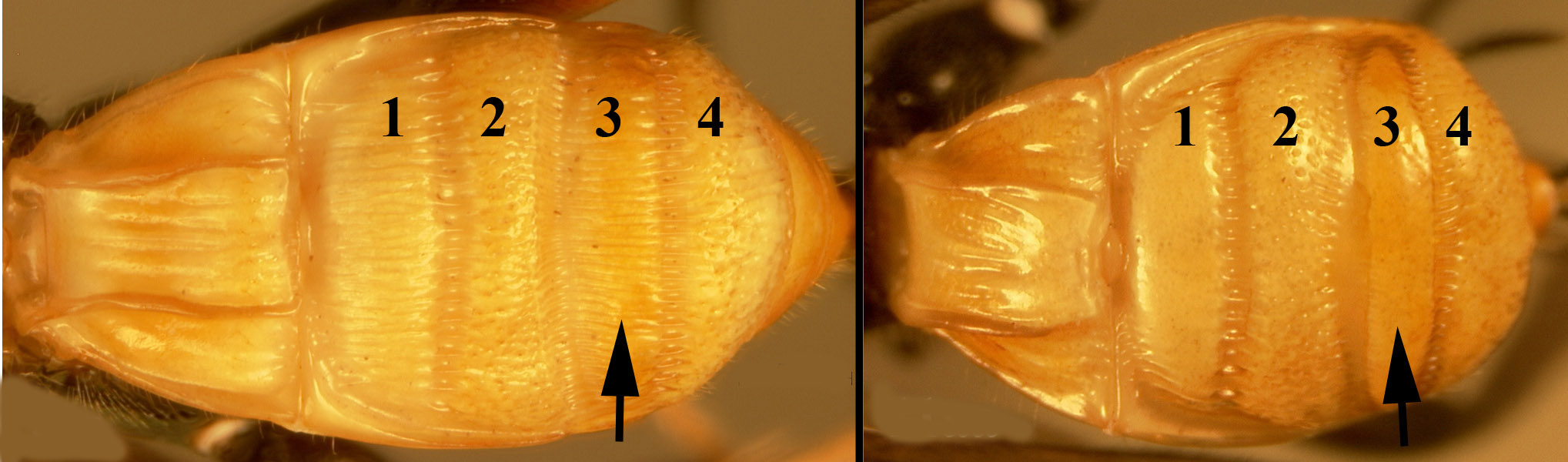

Supplement: Supplementary material 1 — DELTA data matrix, images, and other files [file ZooKeys-130-379-s001.zip › Lytopylus images/key11a.jpg]

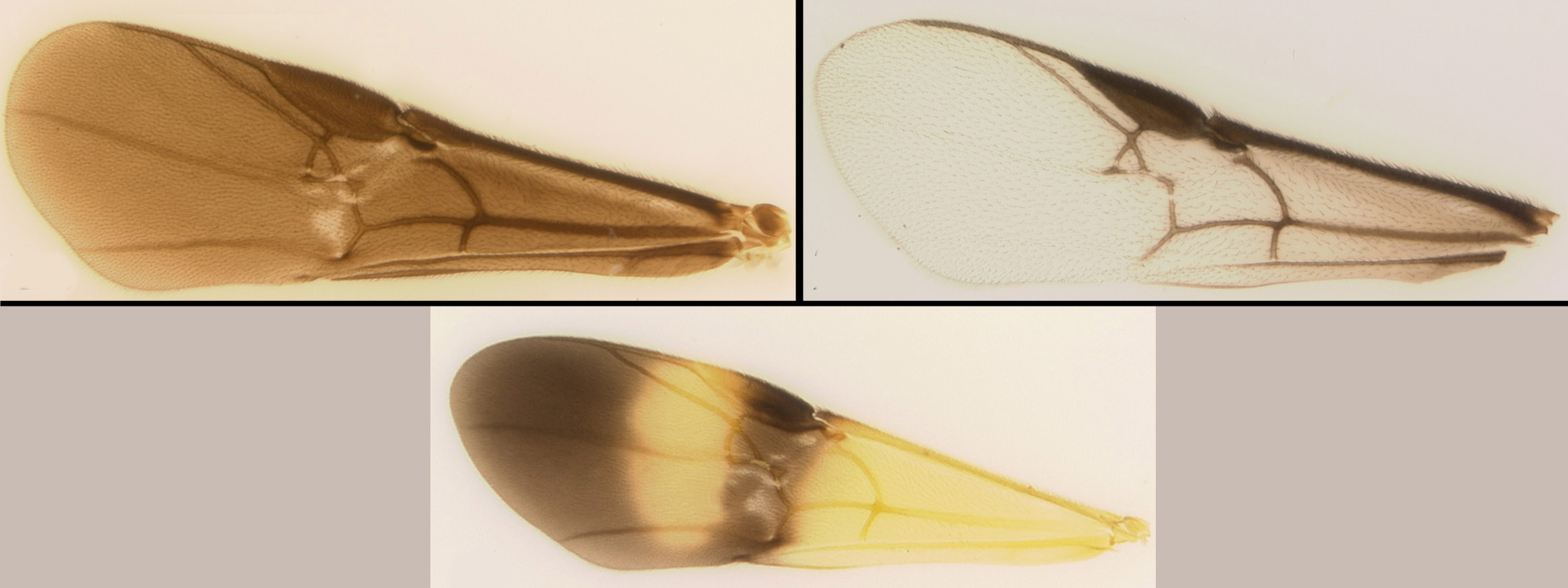

Supplement: Supplementary material 1 — DELTA data matrix, images, and other files [file ZooKeys-130-379-s001.zip › Lytopylus images/key2.jpg]

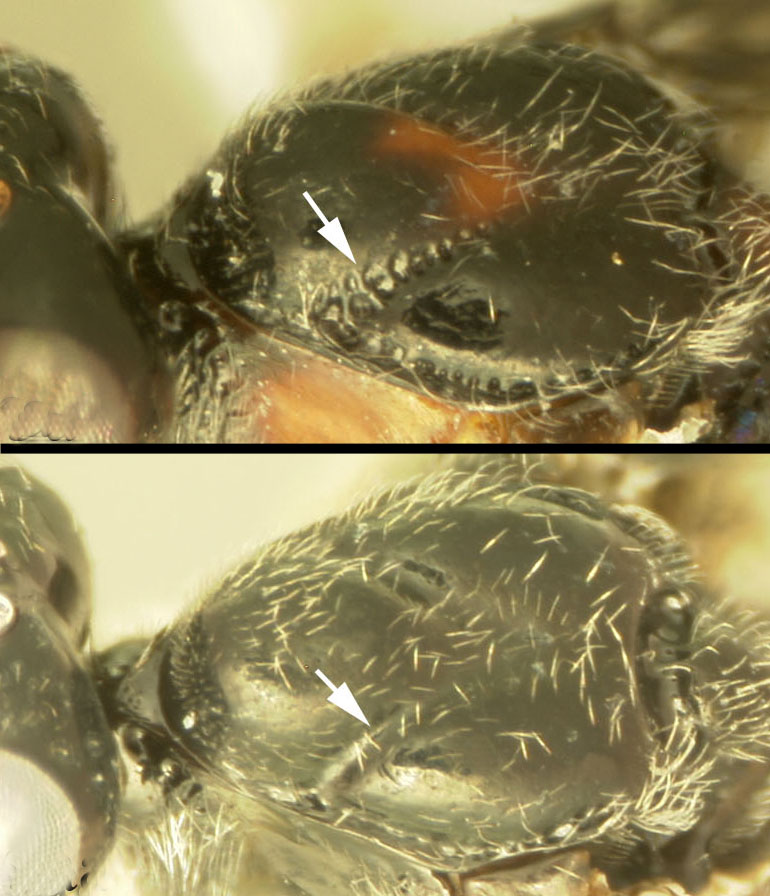

Supplement: Supplementary material 1 — DELTA data matrix, images, and other files [file ZooKeys-130-379-s001.zip › Lytopylus images/key3notauli.jpg]

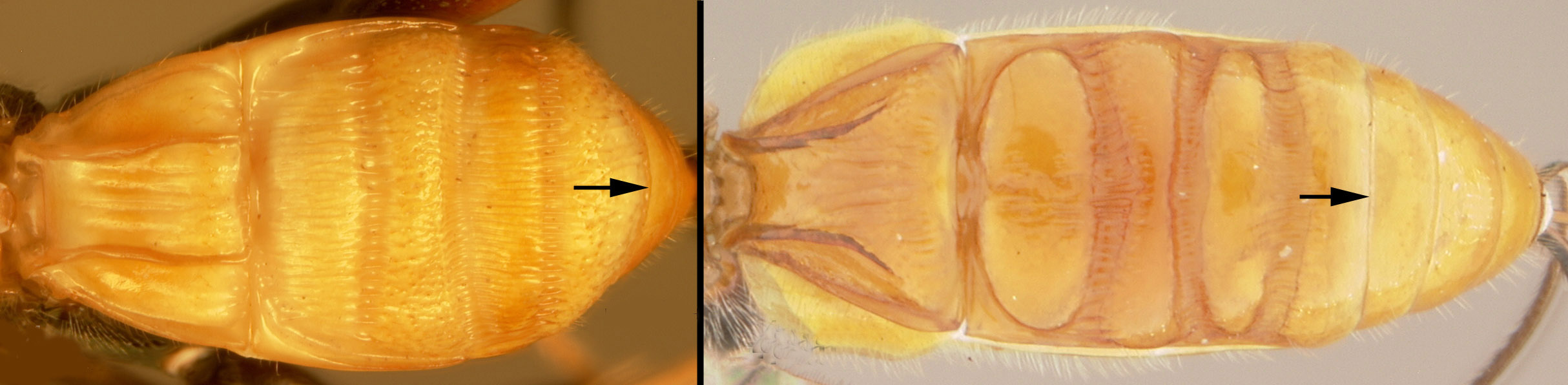

Supplement: Supplementary material 1 — DELTA data matrix, images, and other files [file ZooKeys-130-379-s001.zip › Lytopylus images/key4a.jpg]

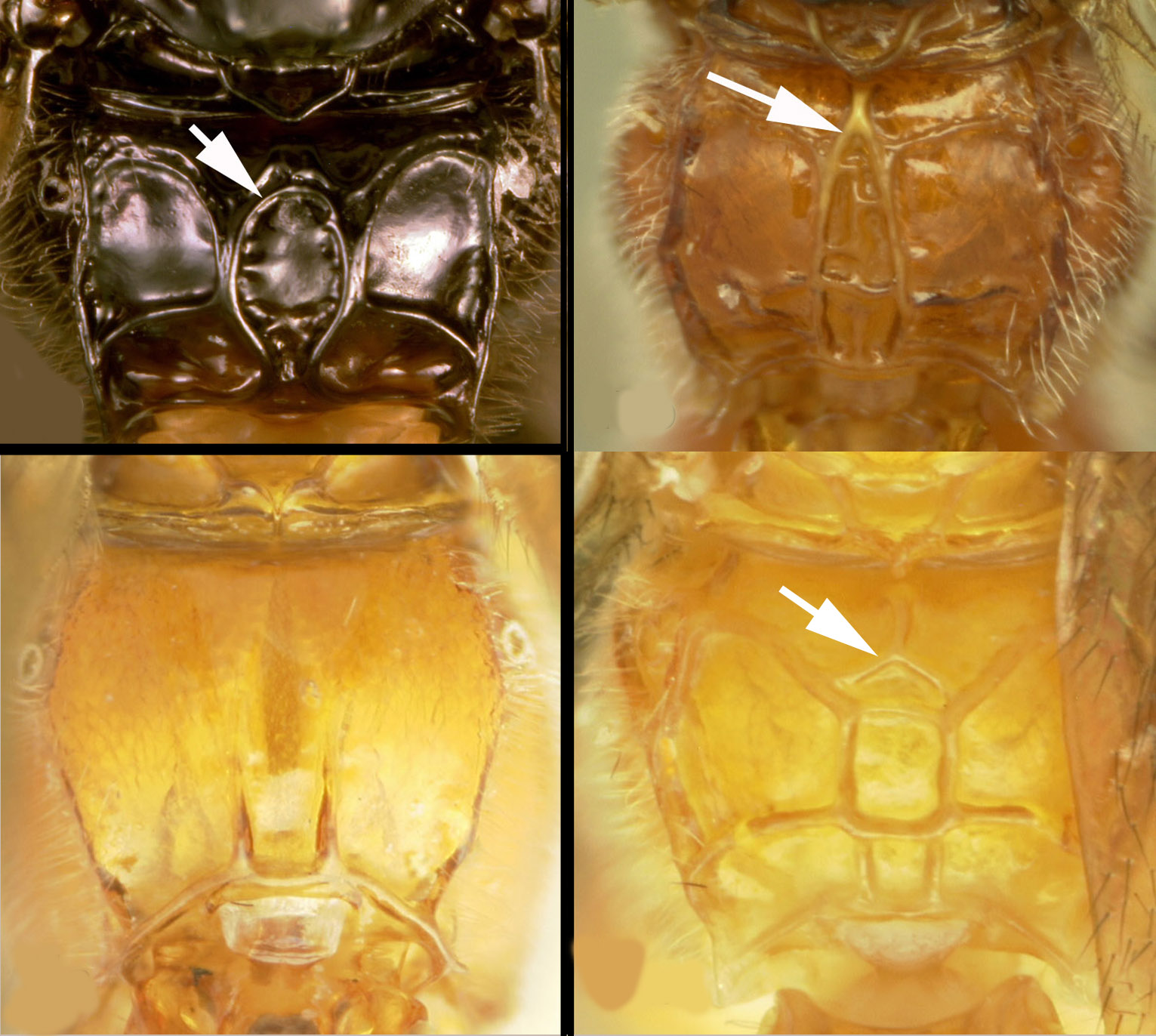

Supplement: Supplementary material 1 — DELTA data matrix, images, and other files [file ZooKeys-130-379-s001.zip › Lytopylus images/key4b.jpg]

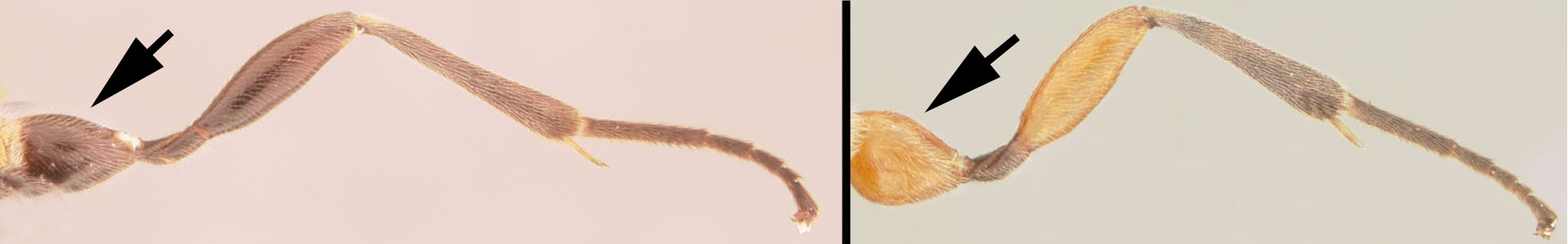

Supplement: Supplementary material 1 — DELTA data matrix, images, and other files [file ZooKeys-130-379-s001.zip › Lytopylus images/key8coxa.jpg]

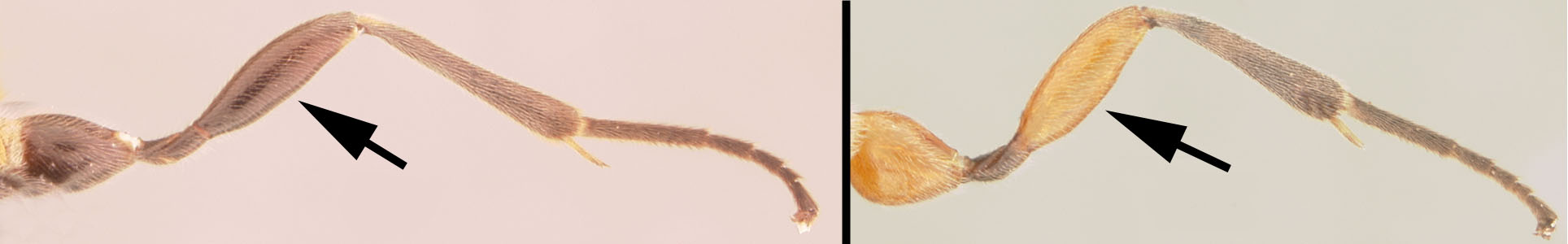

Supplement: Supplementary material 1 — DELTA data matrix, images, and other files [file ZooKeys-130-379-s001.zip › Lytopylus images/key8femur.jpg]

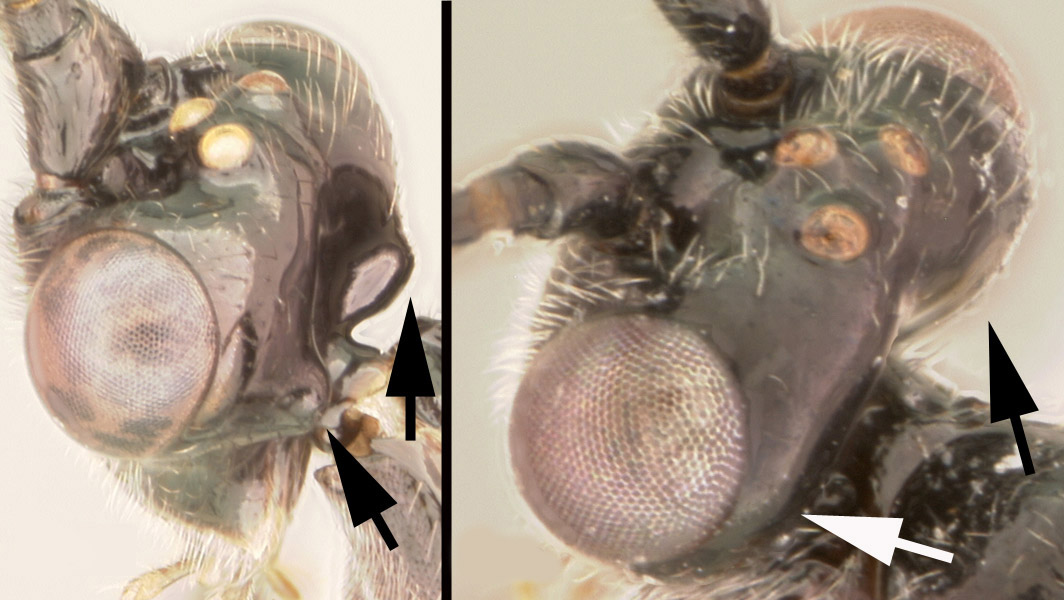

Supplement: Supplementary material 1 — DELTA data matrix, images, and other files [file ZooKeys-130-379-s001.zip › Lytopylus images/key9a.jpg]

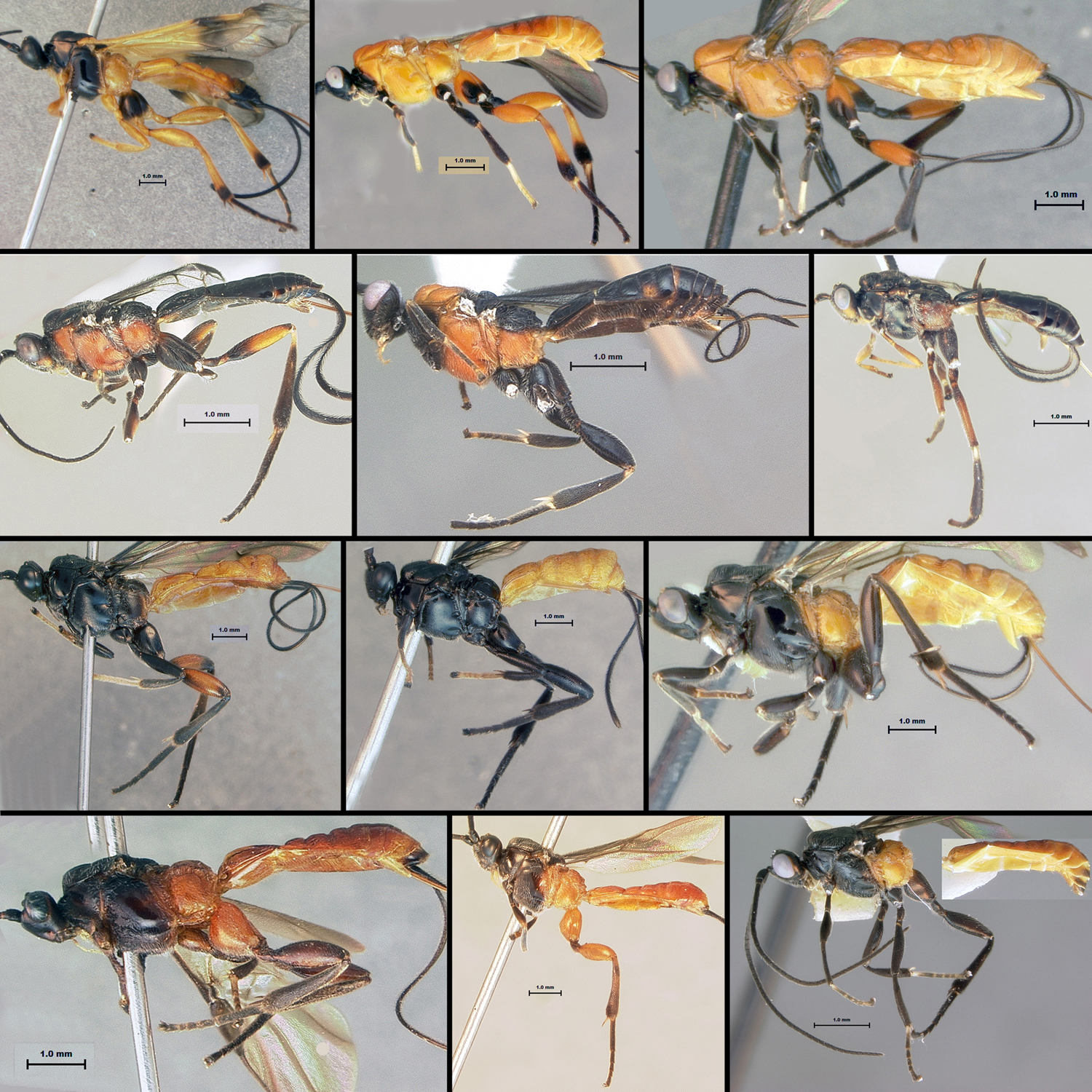

Supplement: Supplementary material 1 — DELTA data matrix, images, and other files [file ZooKeys-130-379-s001.zip › Lytopylus images/lathaball5x5.jpg]

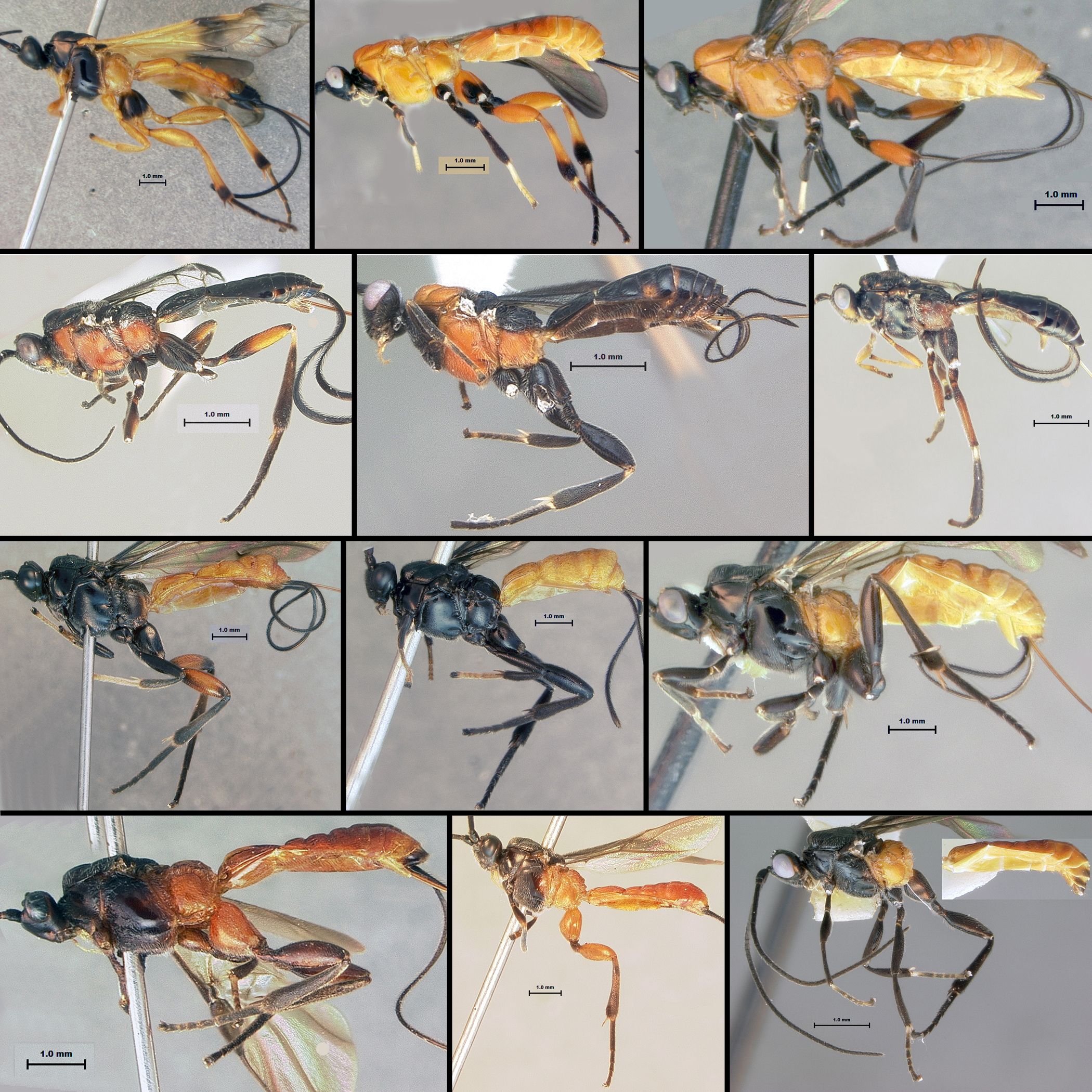

Supplement: Supplementary material 1 — DELTA data matrix, images, and other files [file ZooKeys-130-379-s001.zip › Lytopylus images/lat_hab_all.jpg]

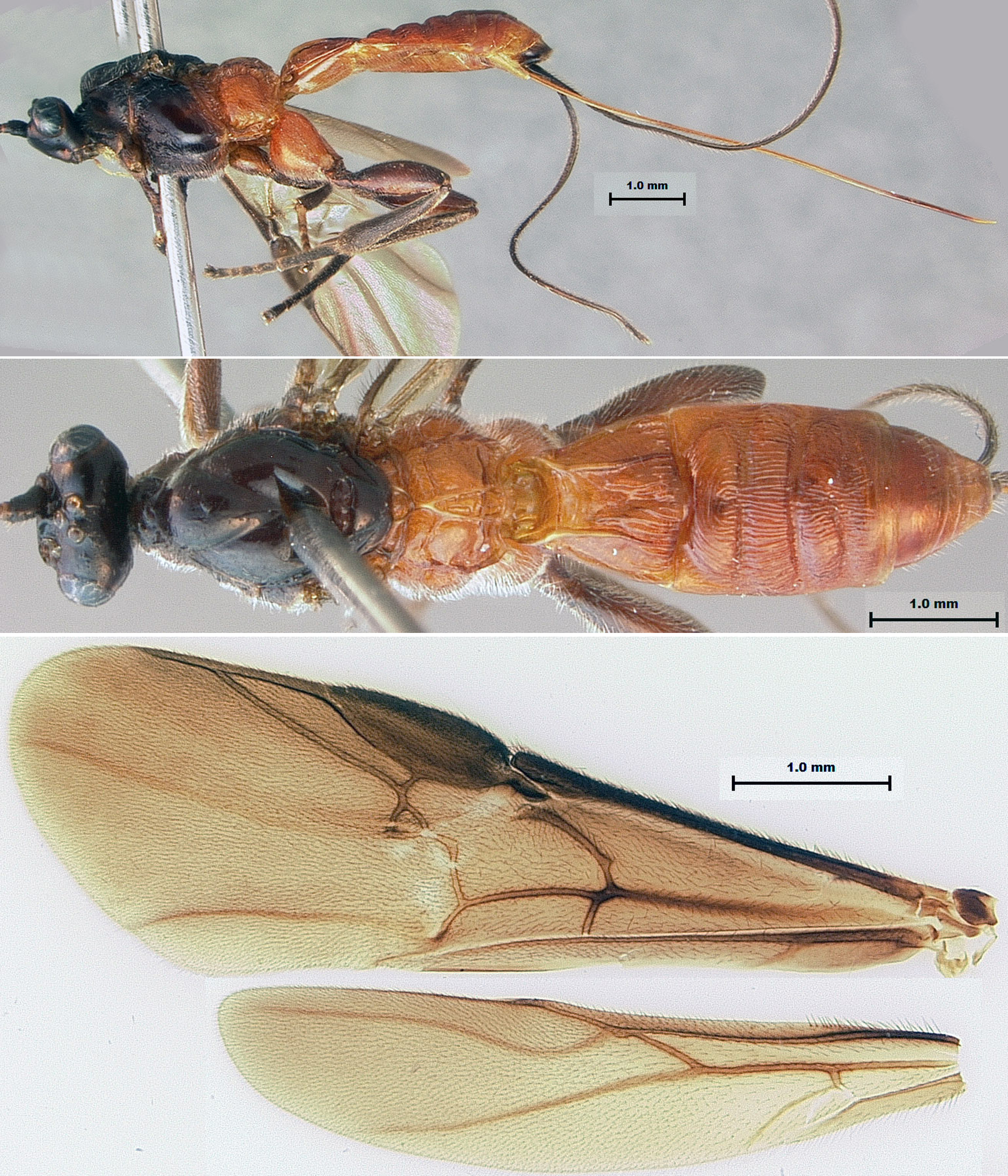

Supplement: Supplementary material 1 — DELTA data matrix, images, and other files [file ZooKeys-130-379-s001.zip › Lytopylus images/macadamaie_plate_1A.jpg]

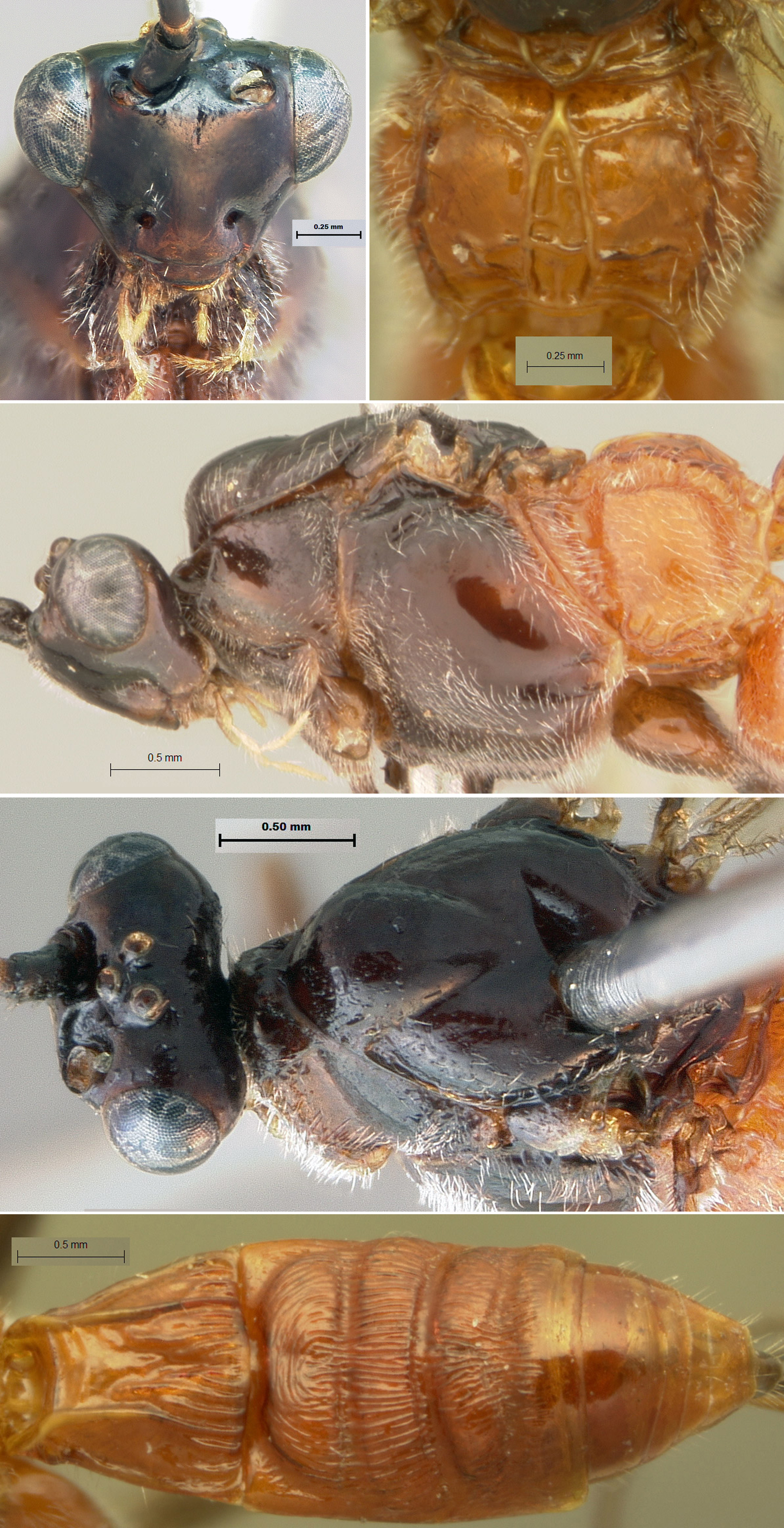

Supplement: Supplementary material 1 — DELTA data matrix, images, and other files [file ZooKeys-130-379-s001.zip › Lytopylus images/macadamaie_plate_2A.jpg]

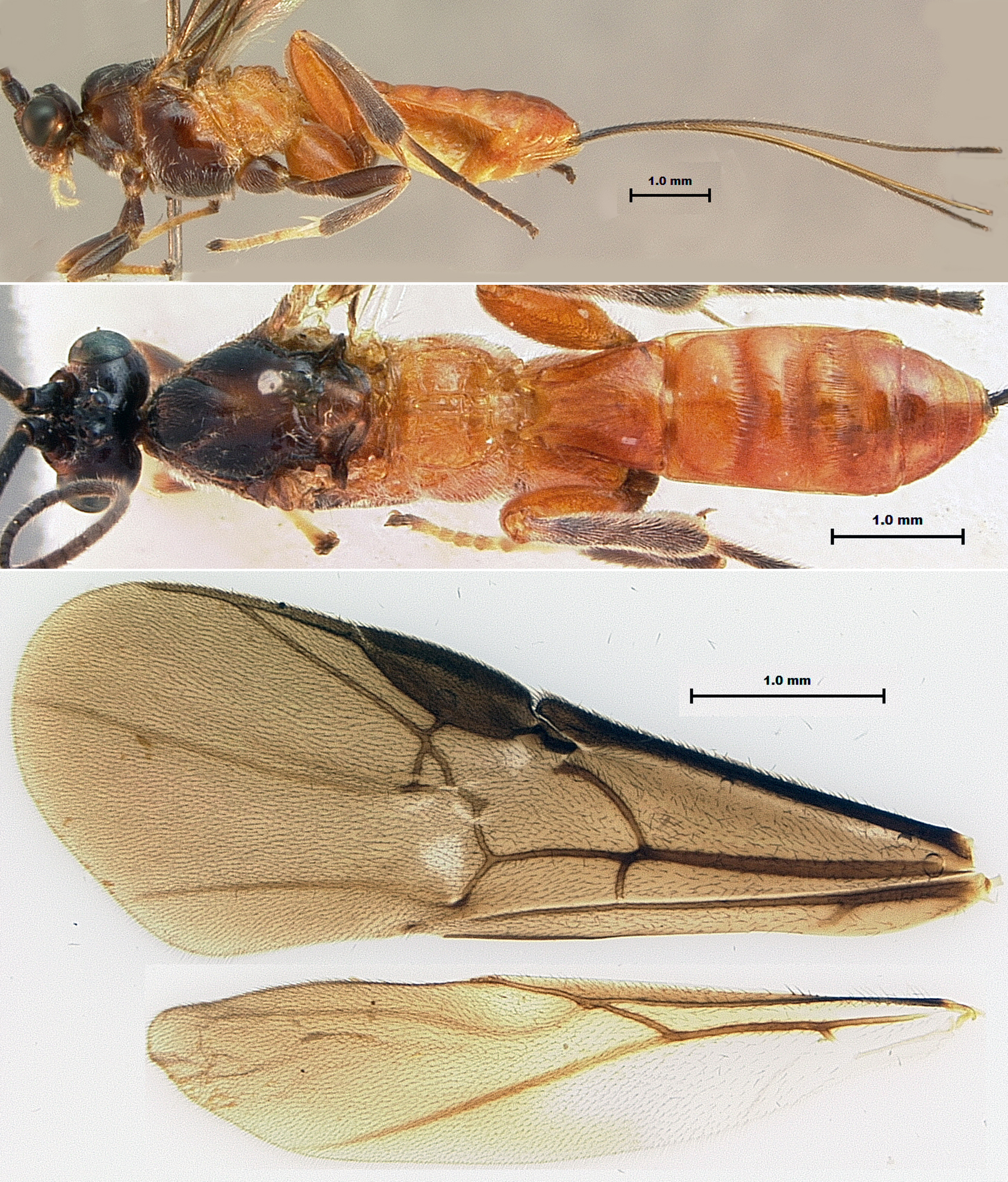

Supplement: Supplementary material 1 — DELTA data matrix, images, and other files [file ZooKeys-130-379-s001.zip › Lytopylus images/plate_1A.jpg]

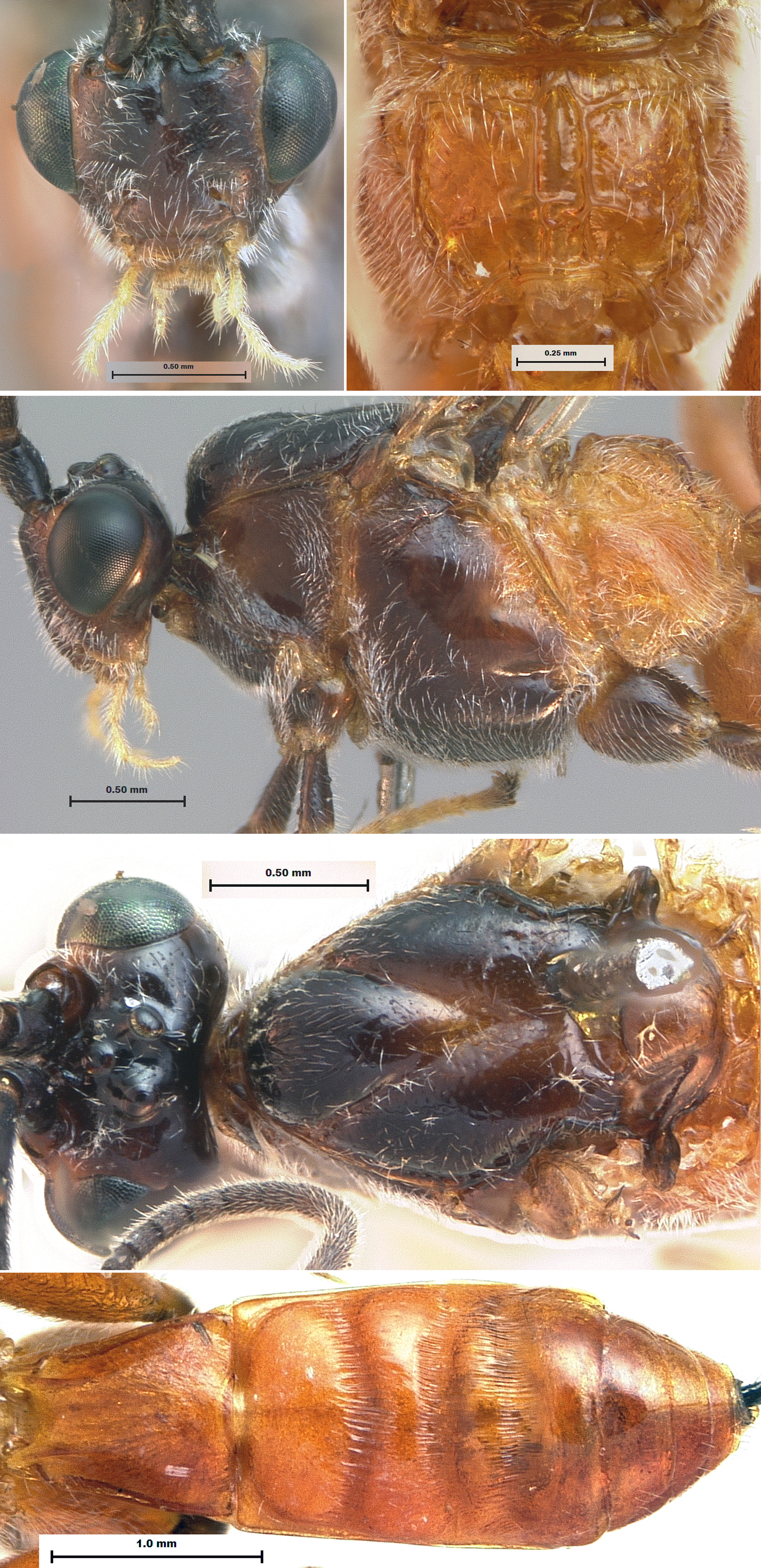

Supplement: Supplementary material 1 — DELTA data matrix, images, and other files [file ZooKeys-130-379-s001.zip › Lytopylus images/plate_2A.jpg]

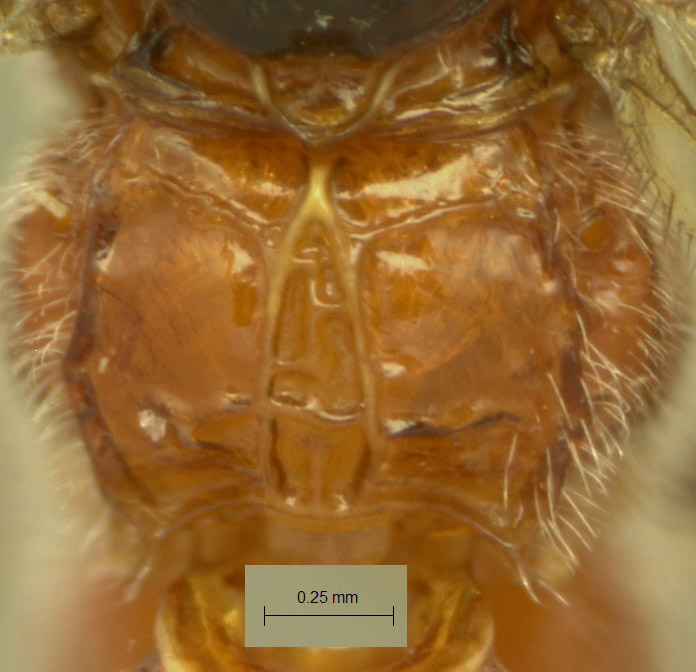

Supplement: Supplementary material 1 — DELTA data matrix, images, and other files [file ZooKeys-130-379-s001.zip › Lytopylus images/PP final.jpg]

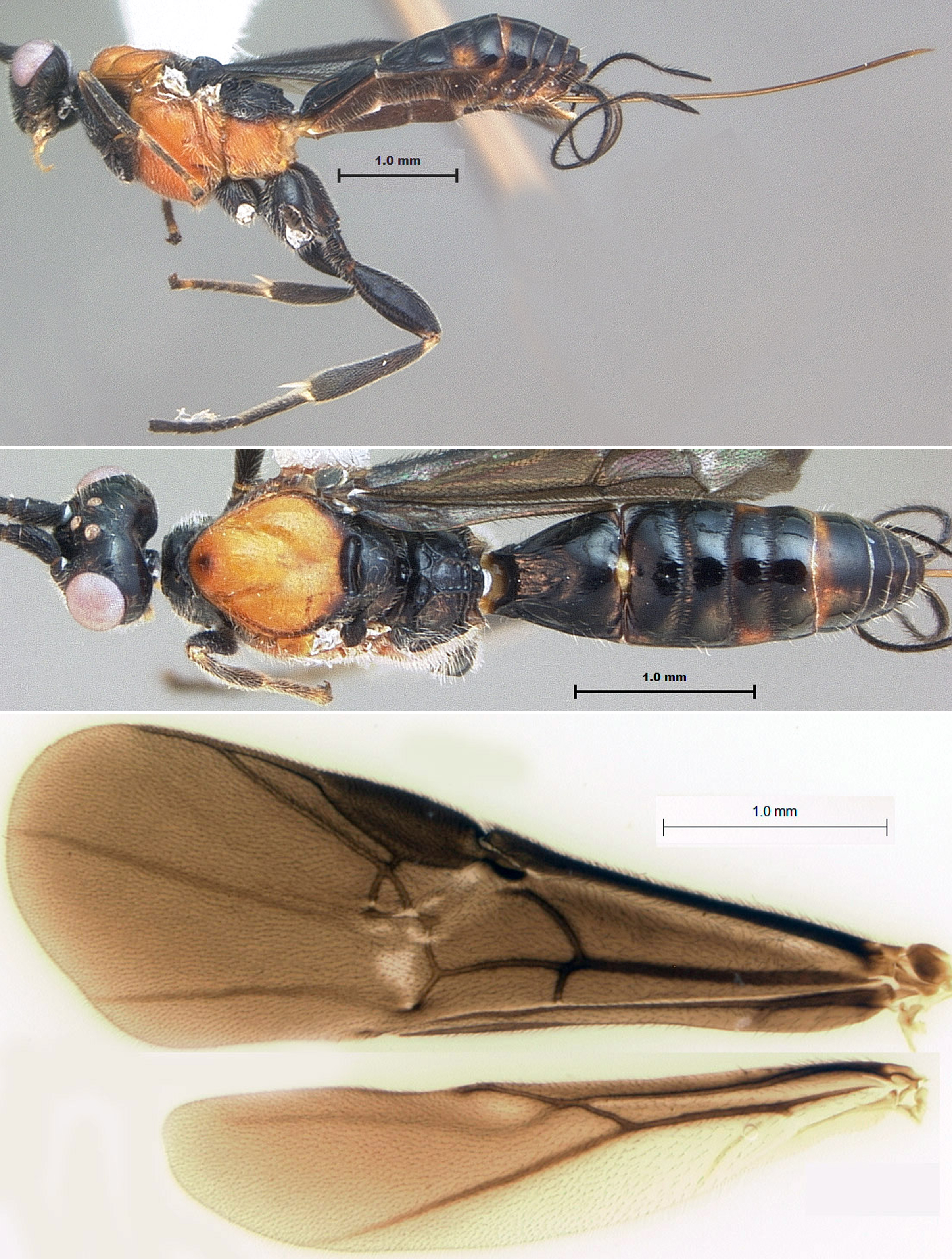

Supplement: Supplementary material 1 — DELTA data matrix, images, and other files [file ZooKeys-130-379-s001.zip › Lytopylus images/sp10_plate_1A.jpg]

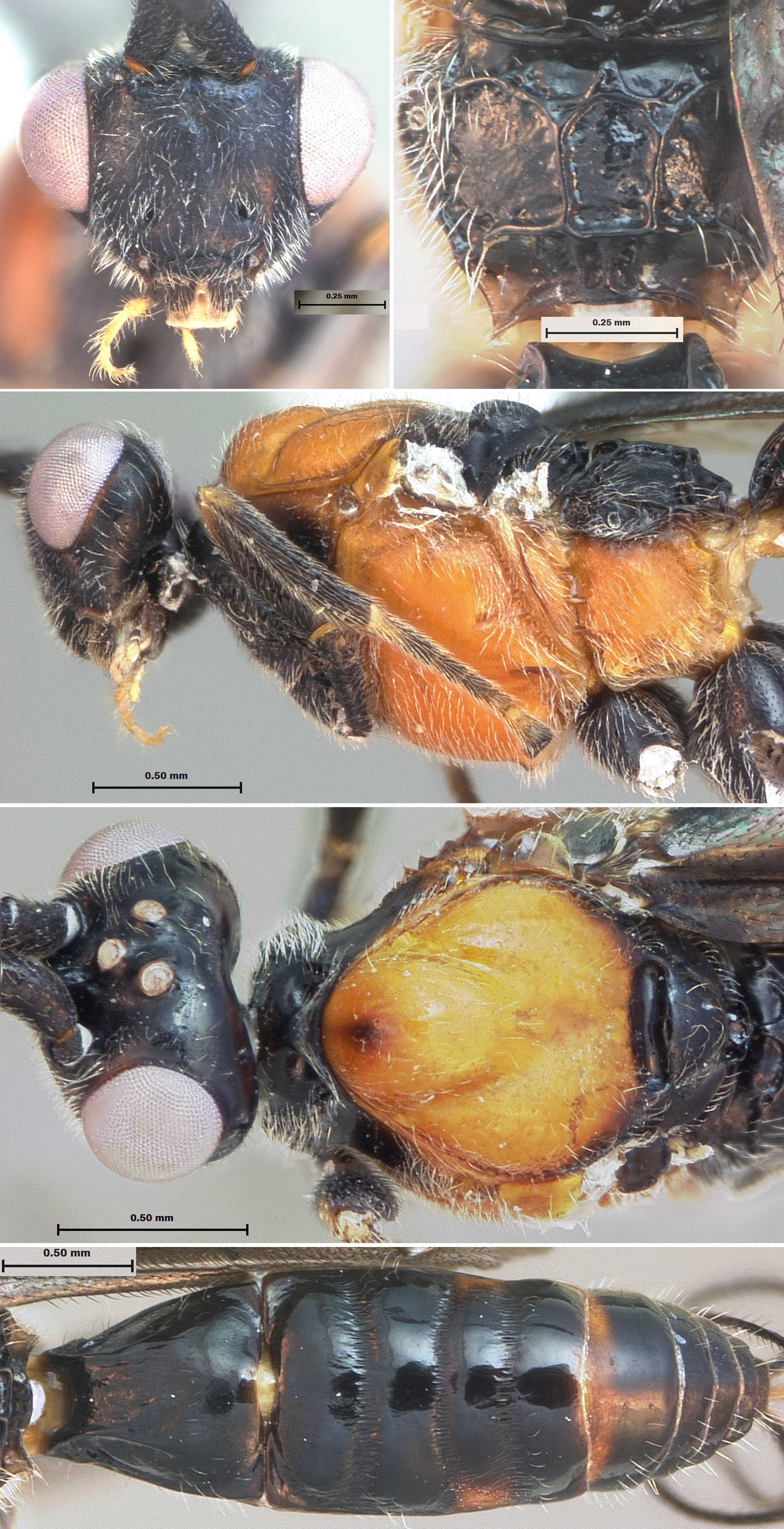

Supplement: Supplementary material 1 — DELTA data matrix, images, and other files [file ZooKeys-130-379-s001.zip › Lytopylus images/sp10_plate_2A.jpg]

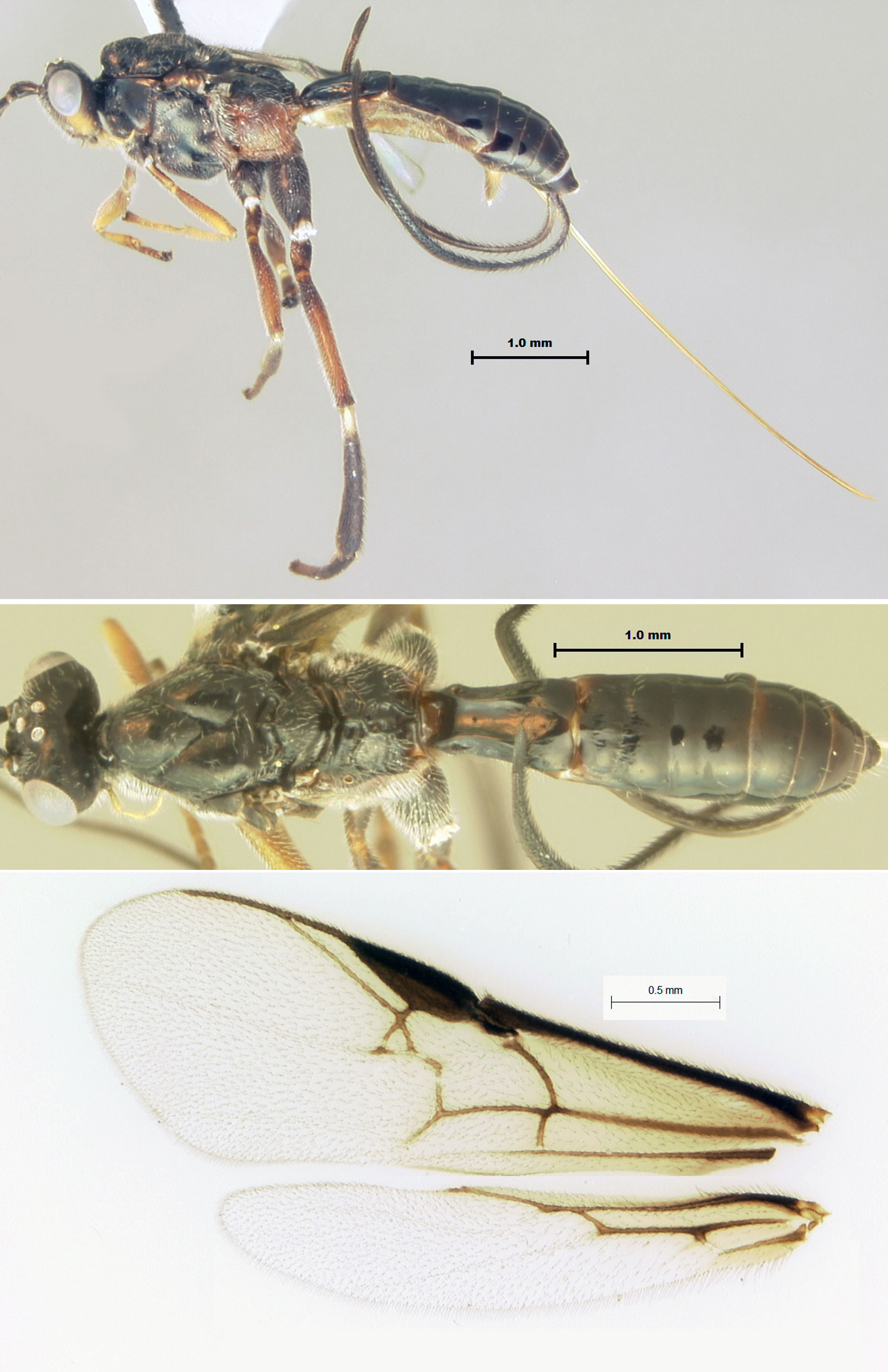

Supplement: Supplementary material 1 — DELTA data matrix, images, and other files [file ZooKeys-130-379-s001.zip › Lytopylus images/sp12_plate_1A.jpg]

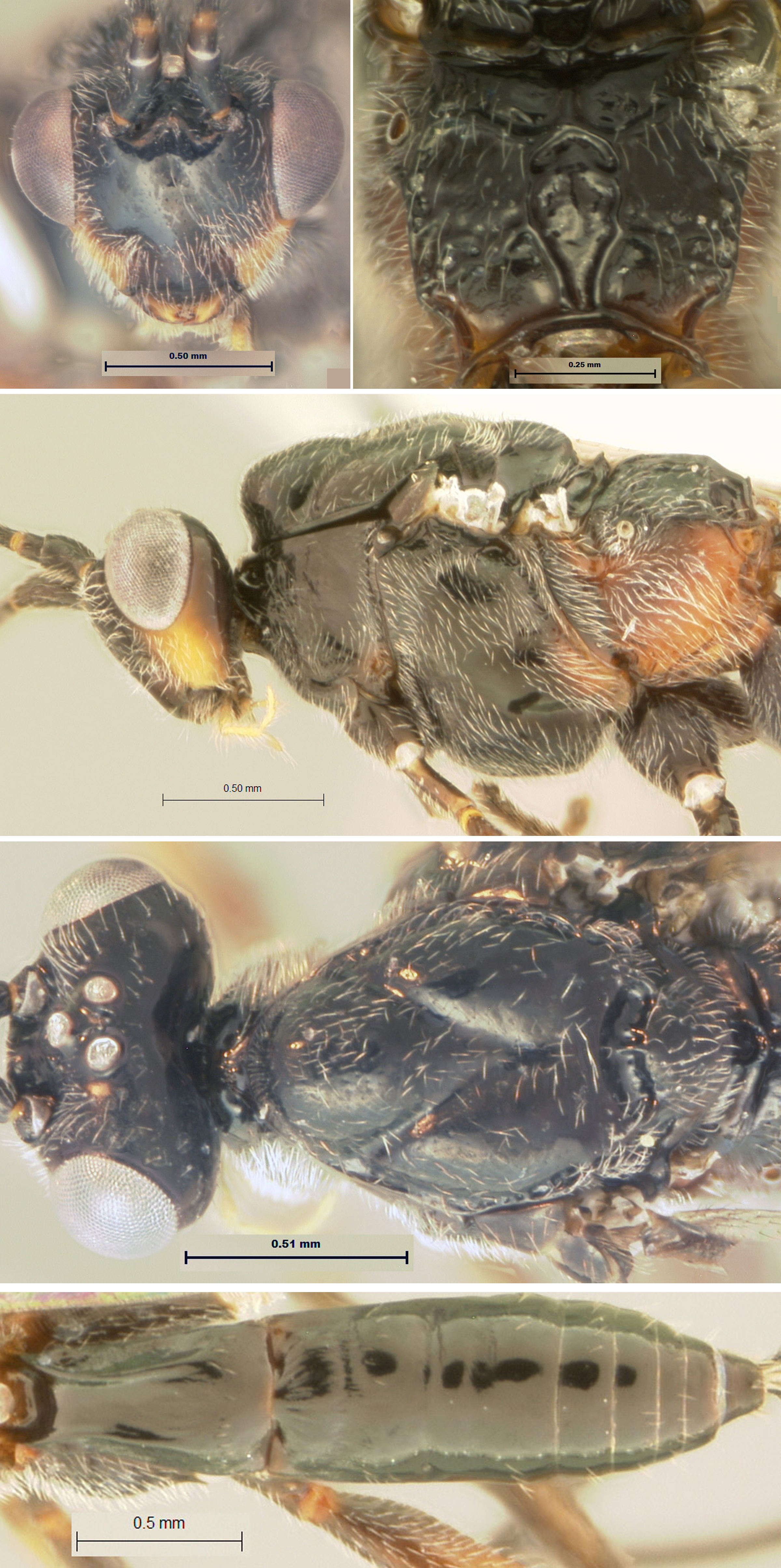

Supplement: Supplementary material 1 — DELTA data matrix, images, and other files [file ZooKeys-130-379-s001.zip › Lytopylus images/sp12_plate_2A.jpg]

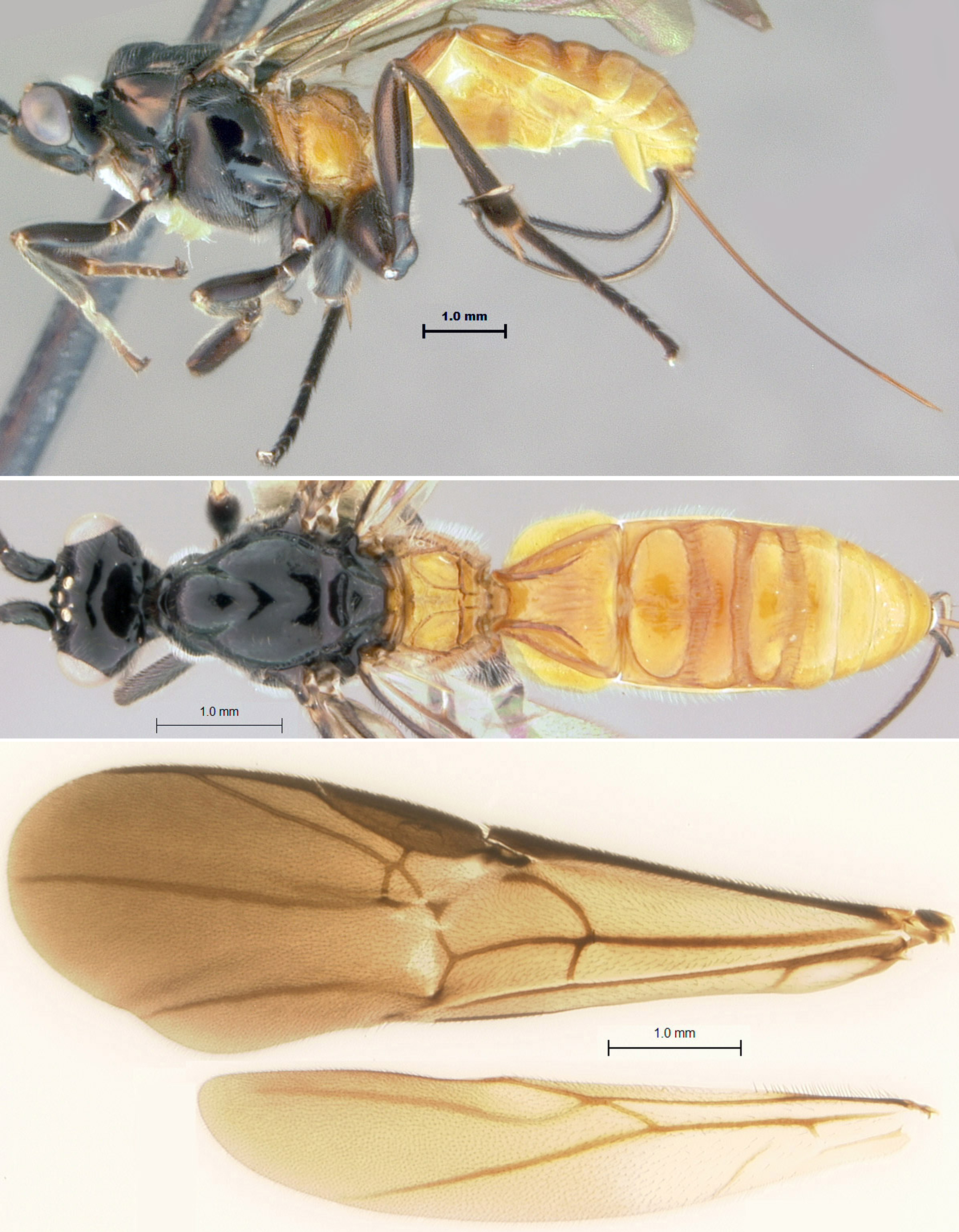

Supplement: Supplementary material 1 — DELTA data matrix, images, and other files [file ZooKeys-130-379-s001.zip › Lytopylus images/sp13_plate_1A.jpg]

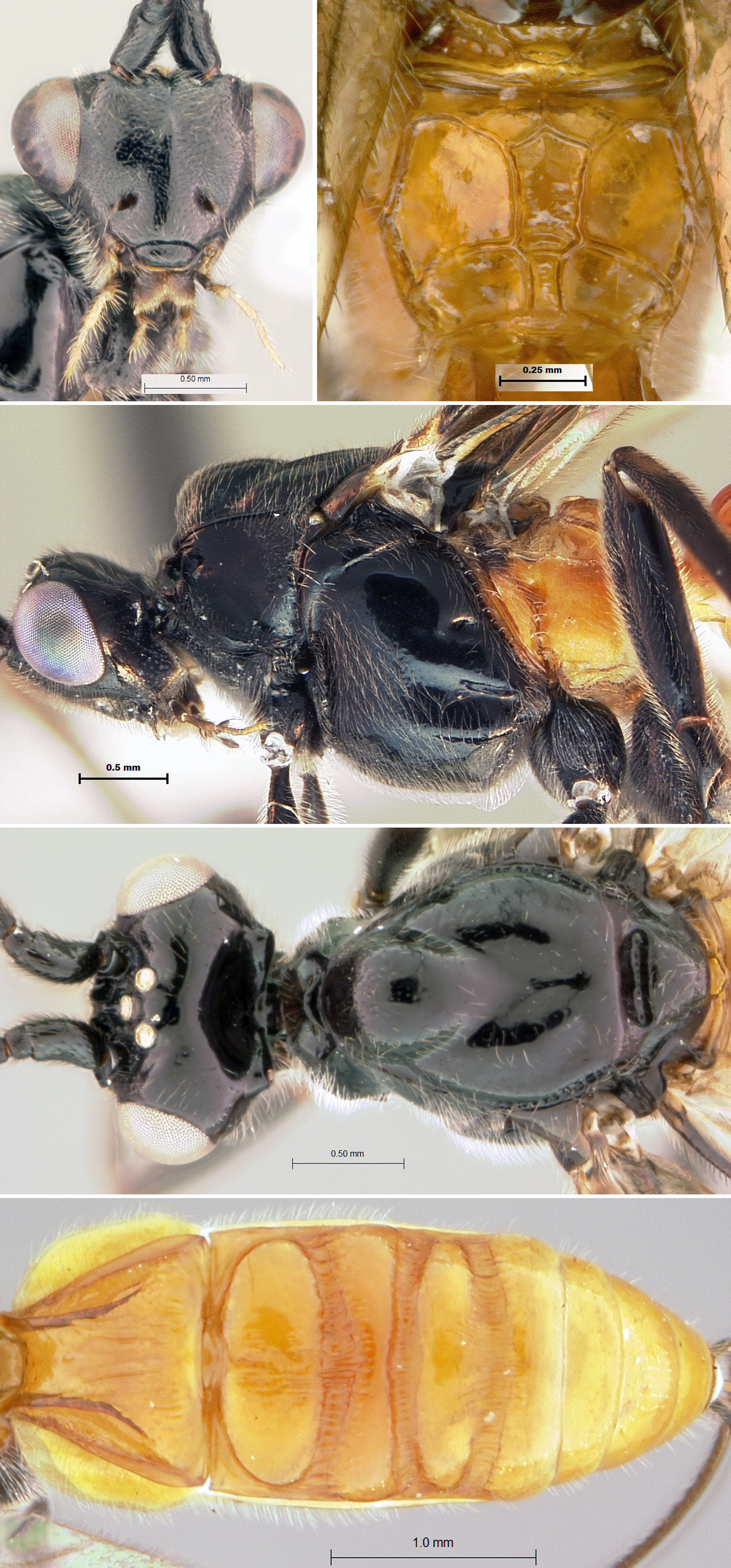

Supplement: Supplementary material 1 — DELTA data matrix, images, and other files [file ZooKeys-130-379-s001.zip › Lytopylus images/sp13_plate_2A.jpg]

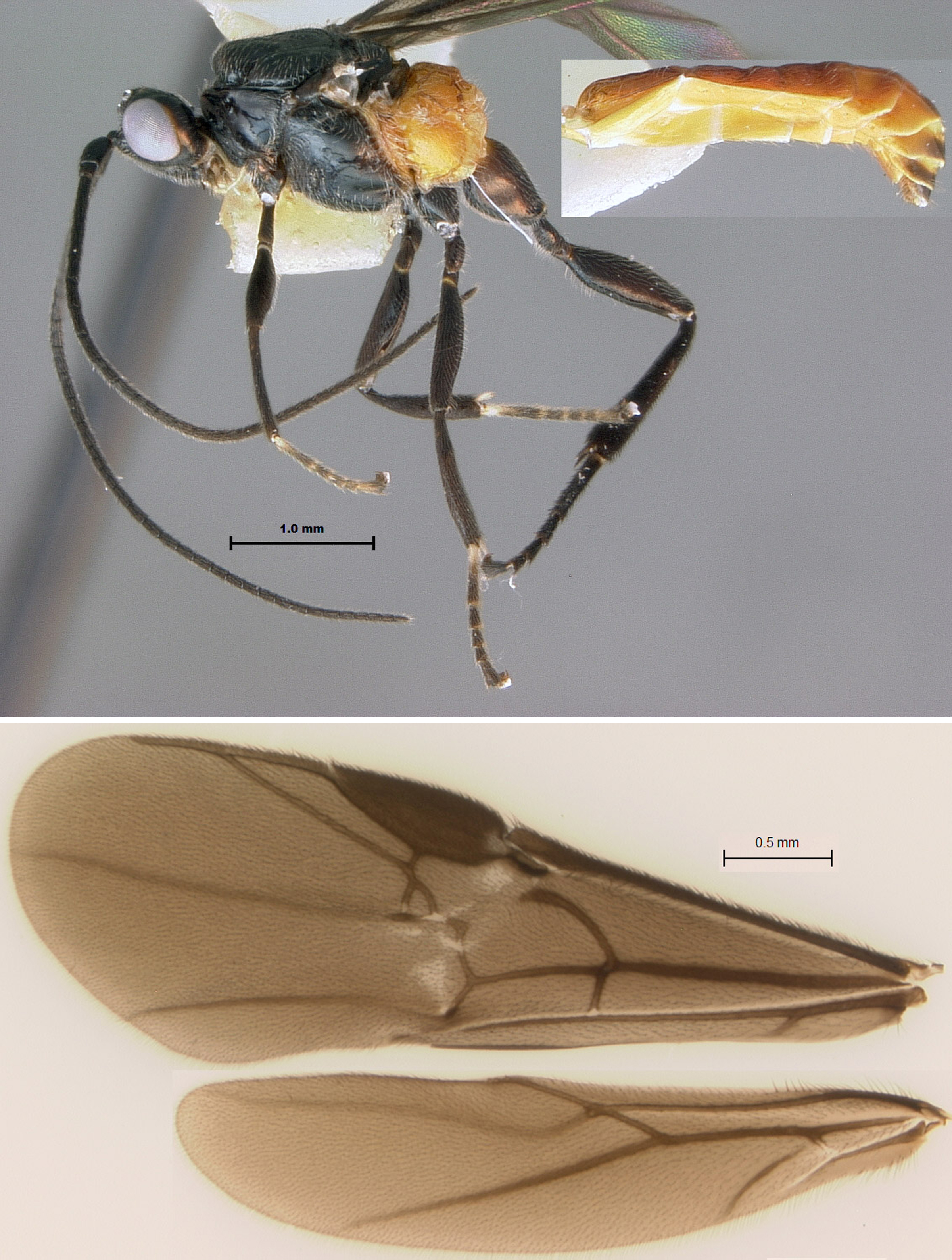

Supplement: Supplementary material 1 — DELTA data matrix, images, and other files [file ZooKeys-130-379-s001.zip › Lytopylus images/sp2_plate_1A.jpg]

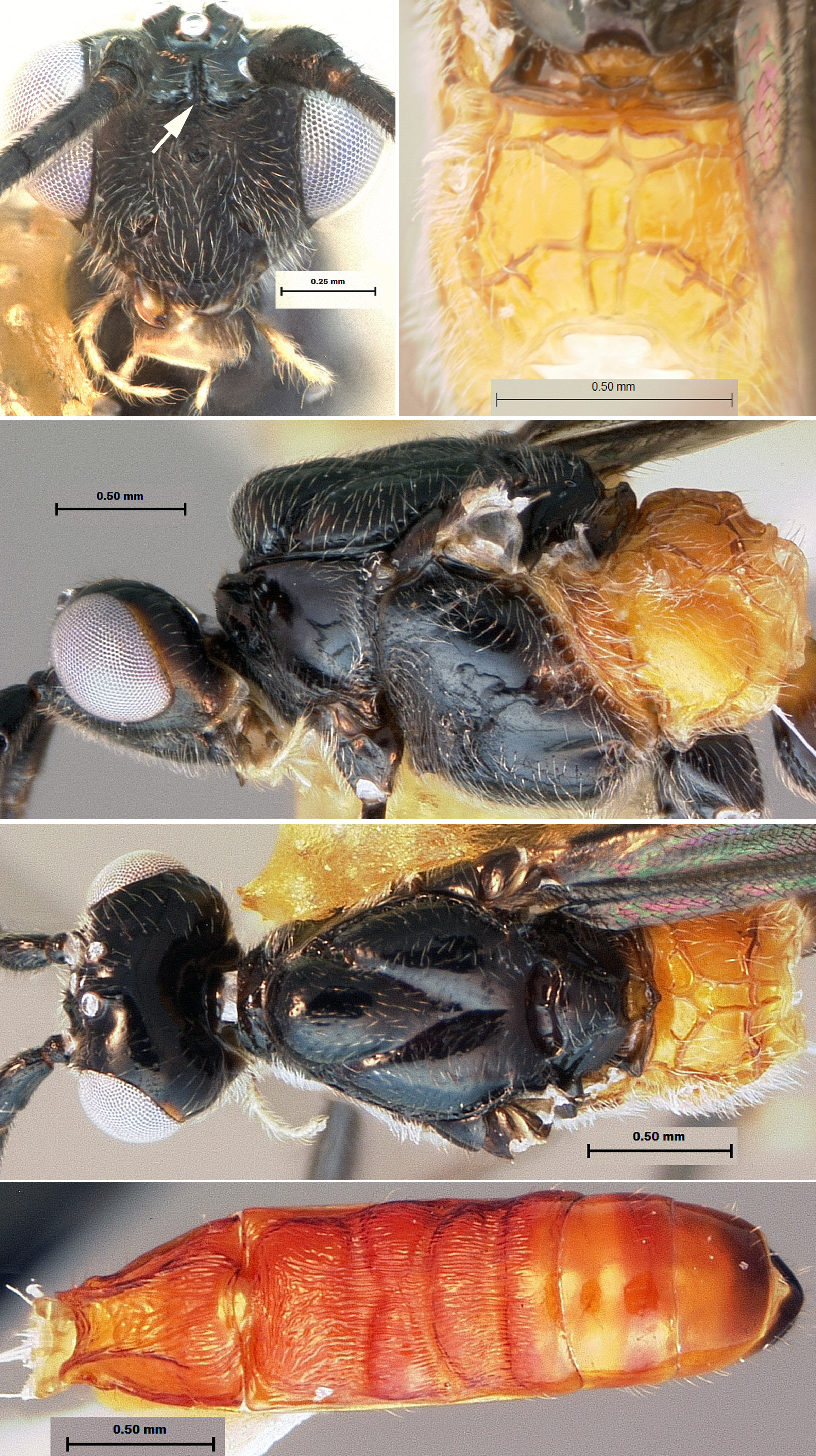

Supplement: Supplementary material 1 — DELTA data matrix, images, and other files [file ZooKeys-130-379-s001.zip › Lytopylus images/sp2_plate_2A.jpg]

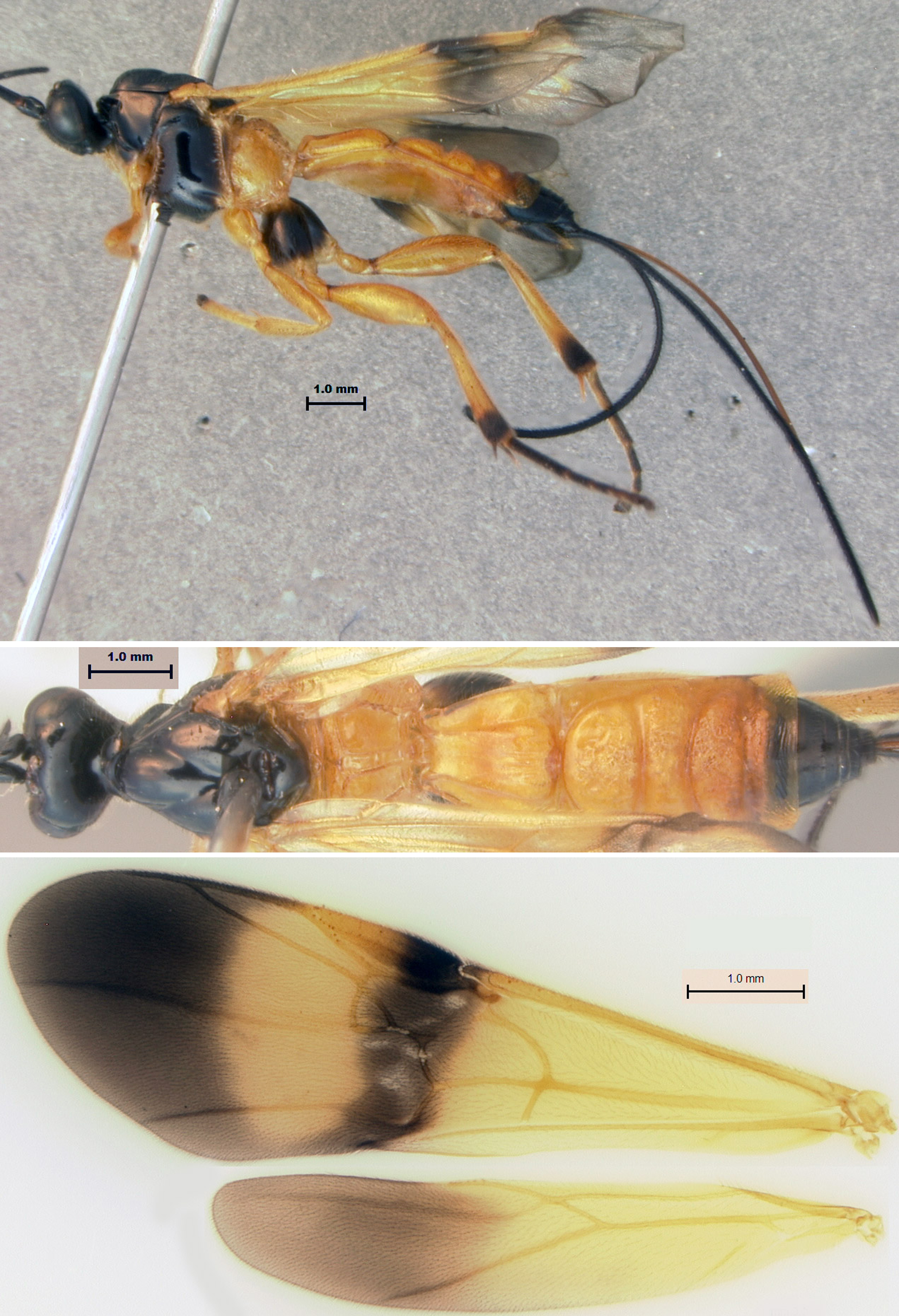

Supplement: Supplementary material 1 — DELTA data matrix, images, and other files [file ZooKeys-130-379-s001.zip › Lytopylus images/sp3_plate_1A.jpg]

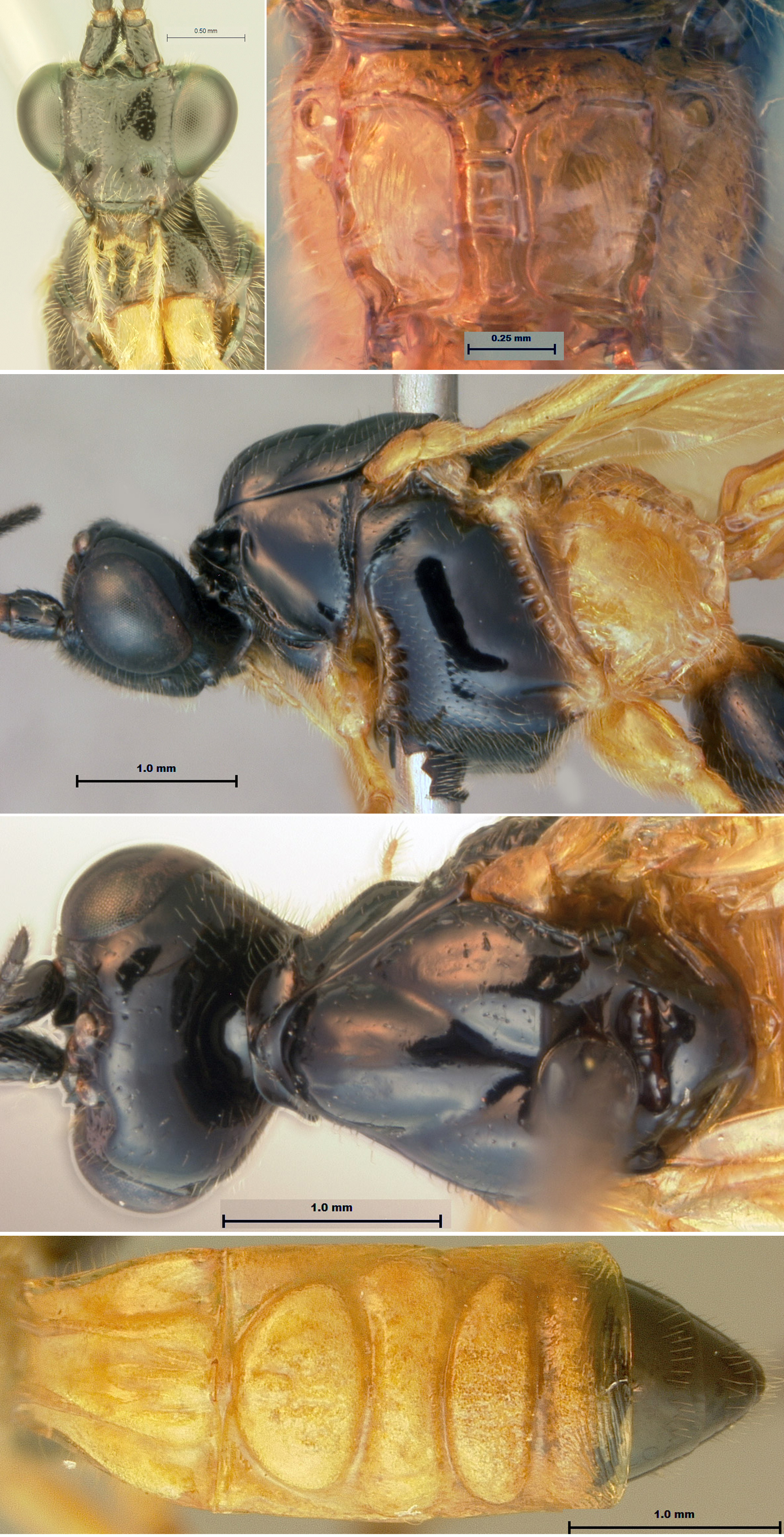

Supplement: Supplementary material 1 — DELTA data matrix, images, and other files [file ZooKeys-130-379-s001.zip › Lytopylus images/sp3_plate_2A.jpg]

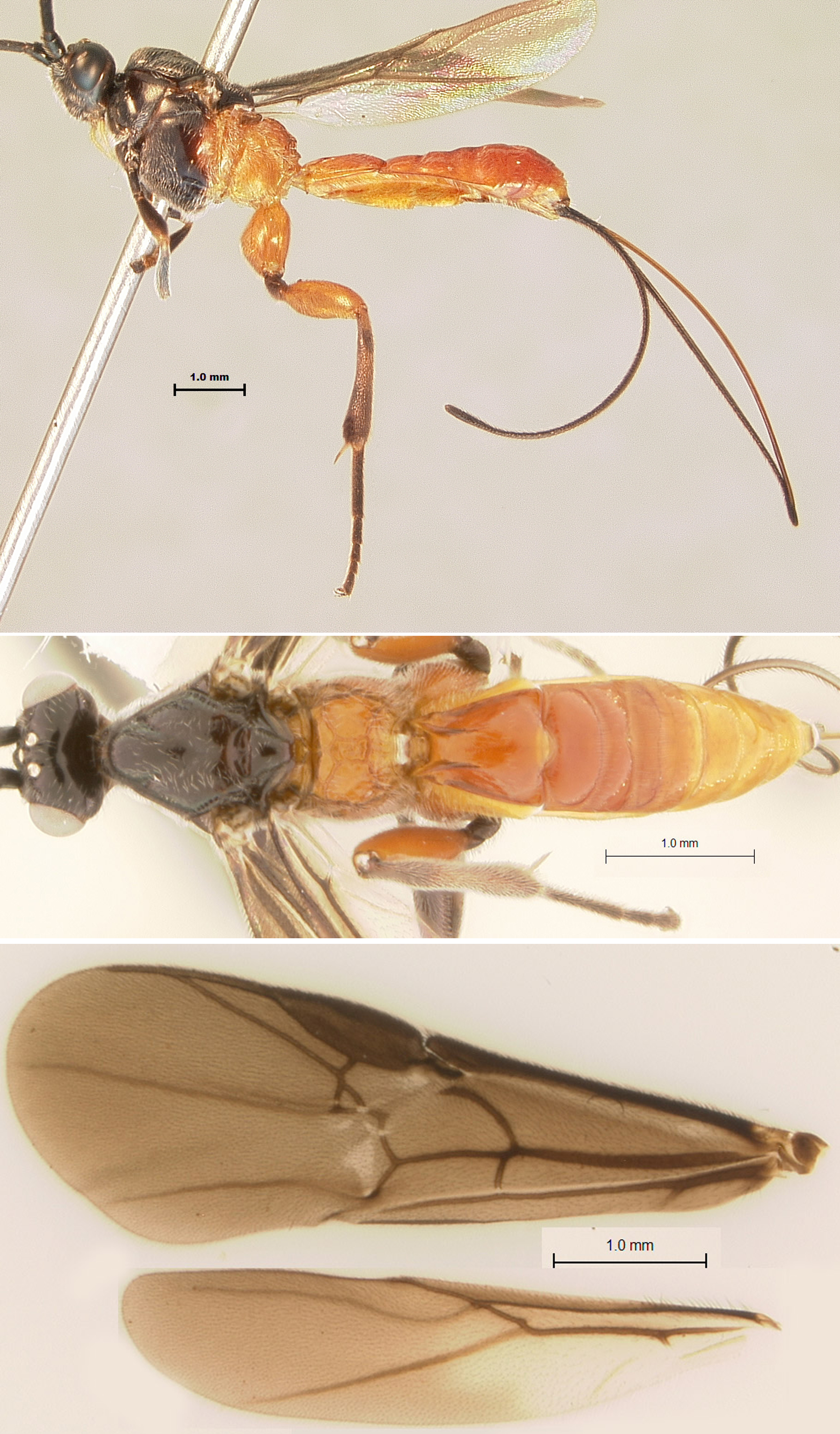

Supplement: Supplementary material 1 — DELTA data matrix, images, and other files [file ZooKeys-130-379-s001.zip › Lytopylus images/sp5_plate_1A.jpg]

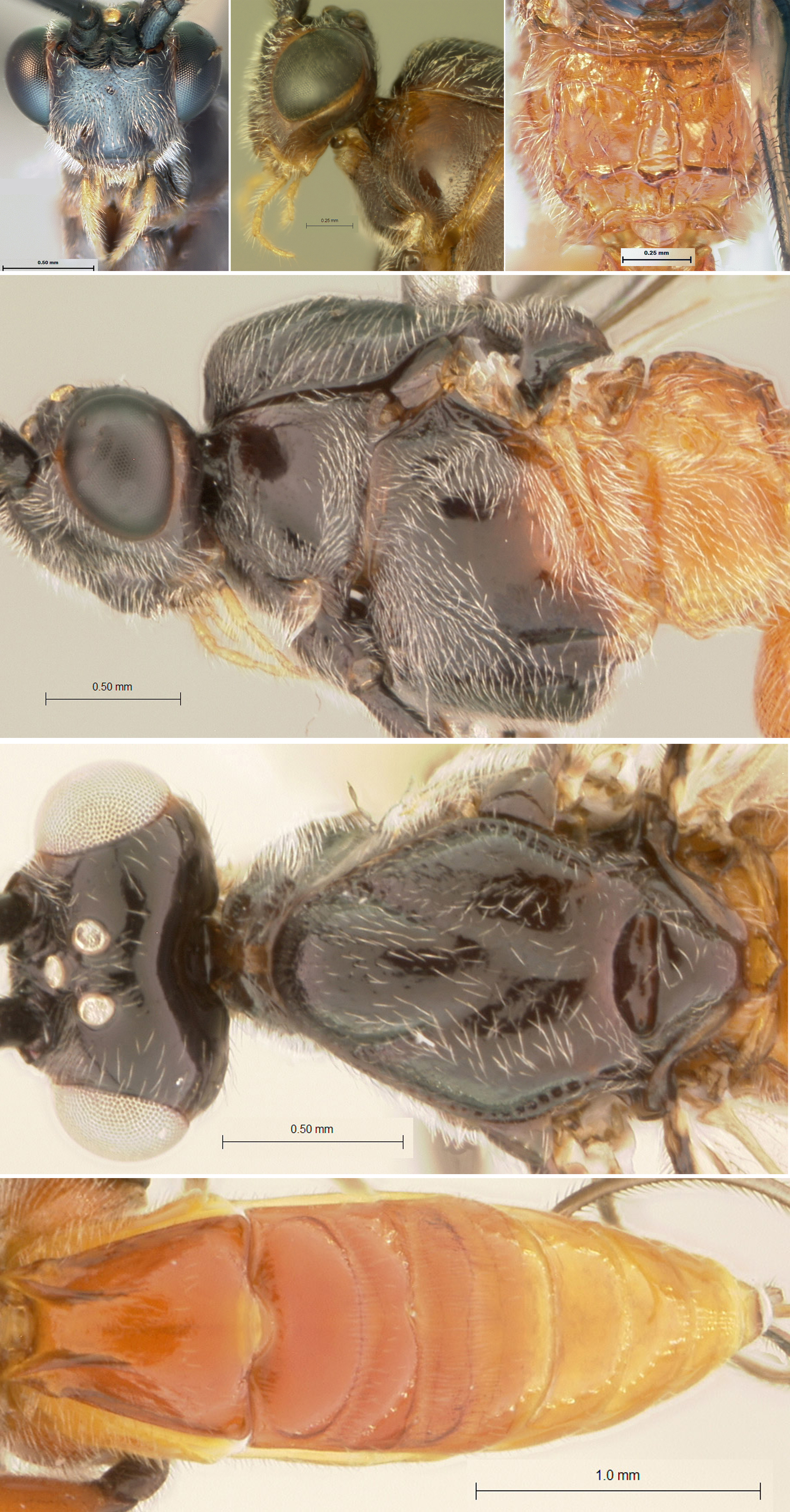

Supplement: Supplementary material 1 — DELTA data matrix, images, and other files [file ZooKeys-130-379-s001.zip › Lytopylus images/sp5_plate_2A.jpg]

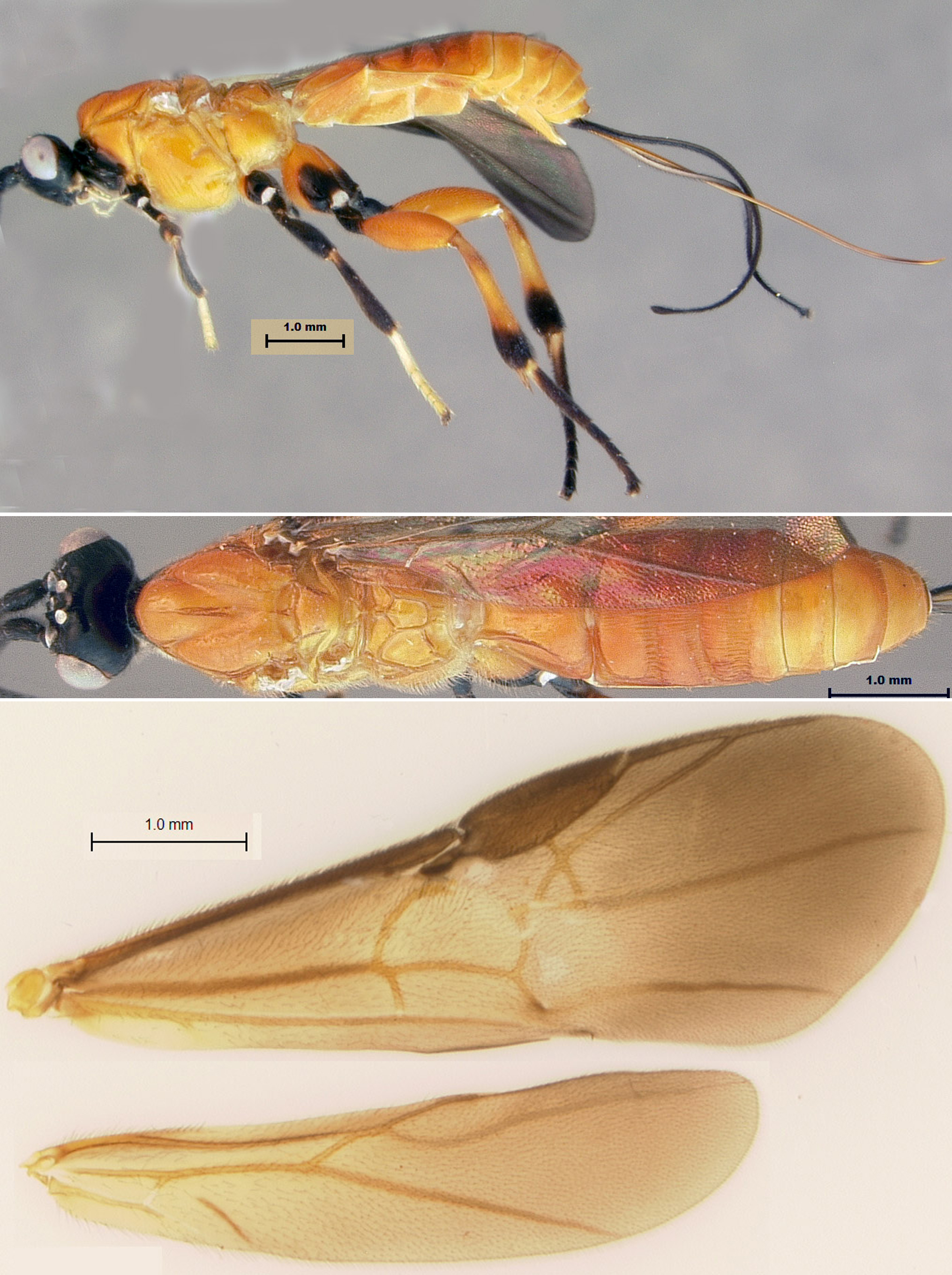

Supplement: Supplementary material 1 — DELTA data matrix, images, and other files [file ZooKeys-130-379-s001.zip › Lytopylus images/sp6_plate_1A.jpg]

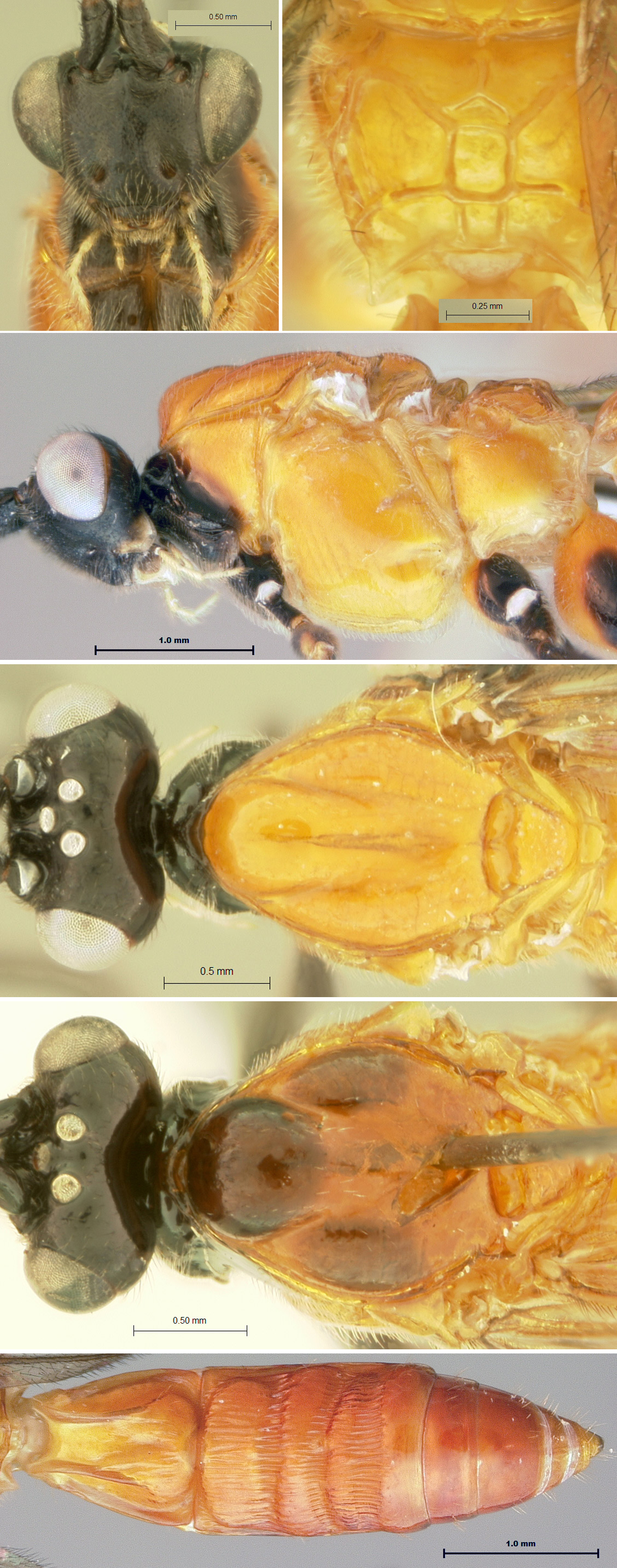

Supplement: Supplementary material 1 — DELTA data matrix, images, and other files [file ZooKeys-130-379-s001.zip › Lytopylus images/sp6_plate_2A.jpg]

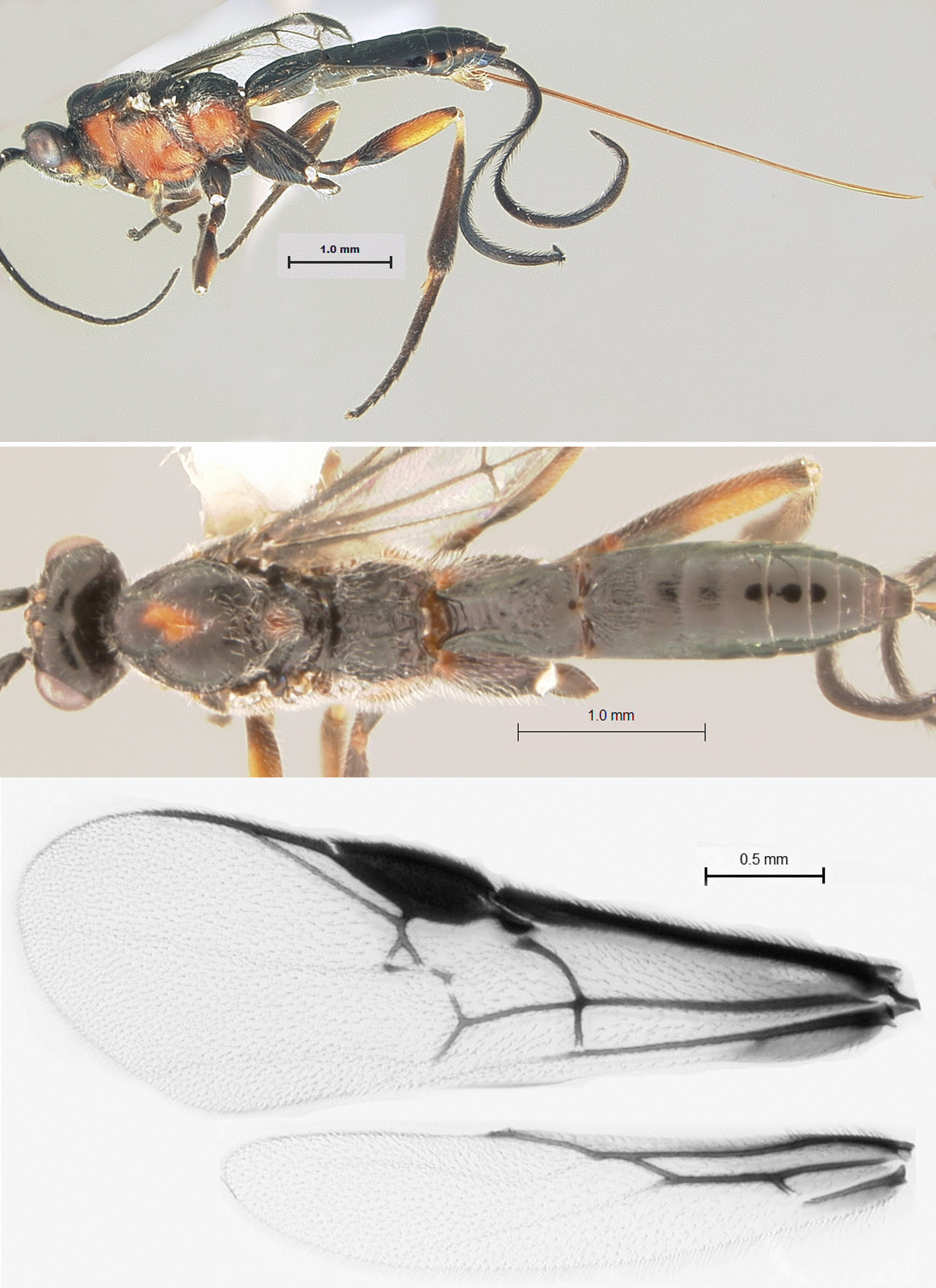

Supplement: Supplementary material 1 — DELTA data matrix, images, and other files [file ZooKeys-130-379-s001.zip › Lytopylus images/sp9_plate_1A.jpg]

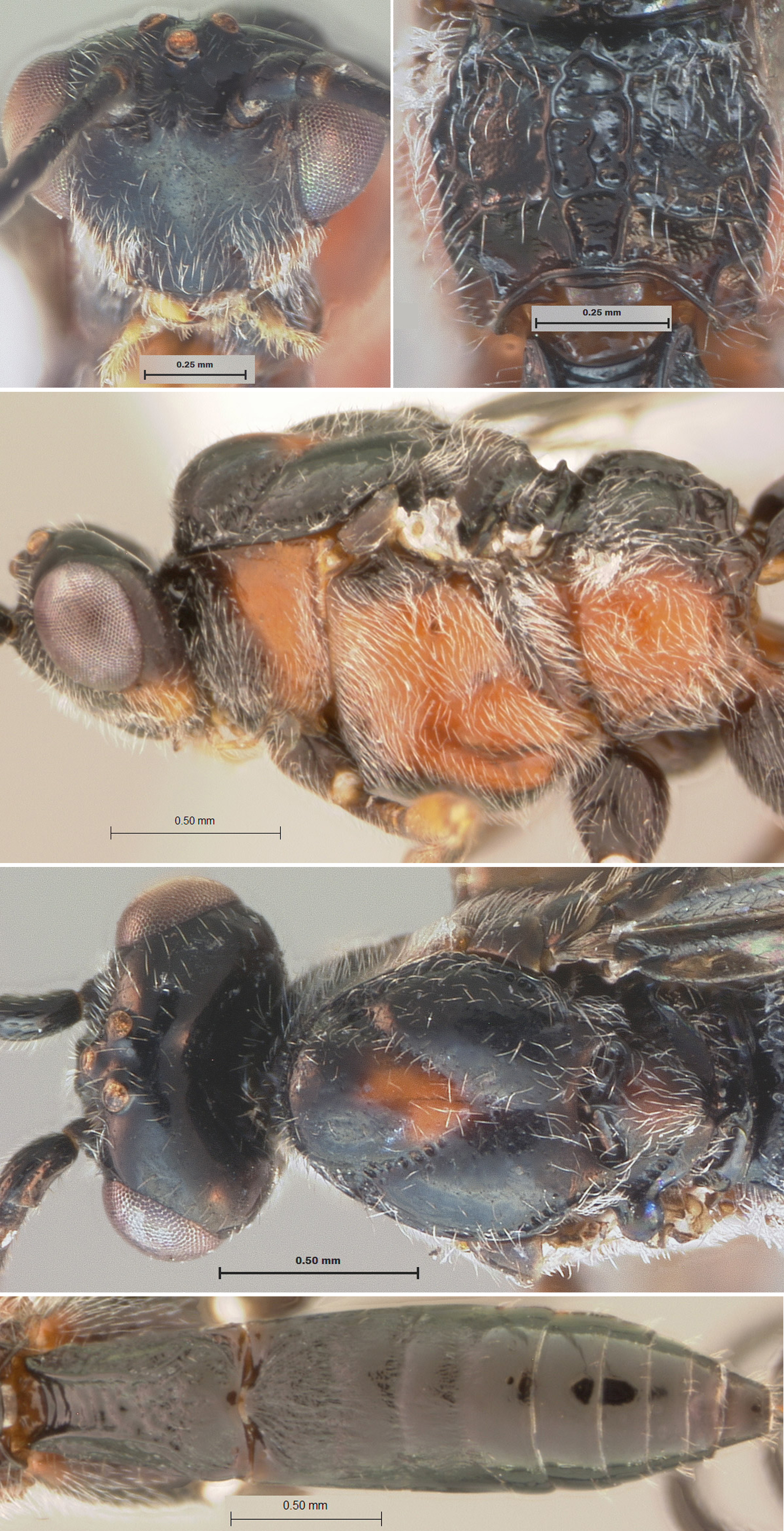

Supplement: Supplementary material 1 — DELTA data matrix, images, and other files [file ZooKeys-130-379-s001.zip › Lytopylus images/sp9_plate_2A.jpg]

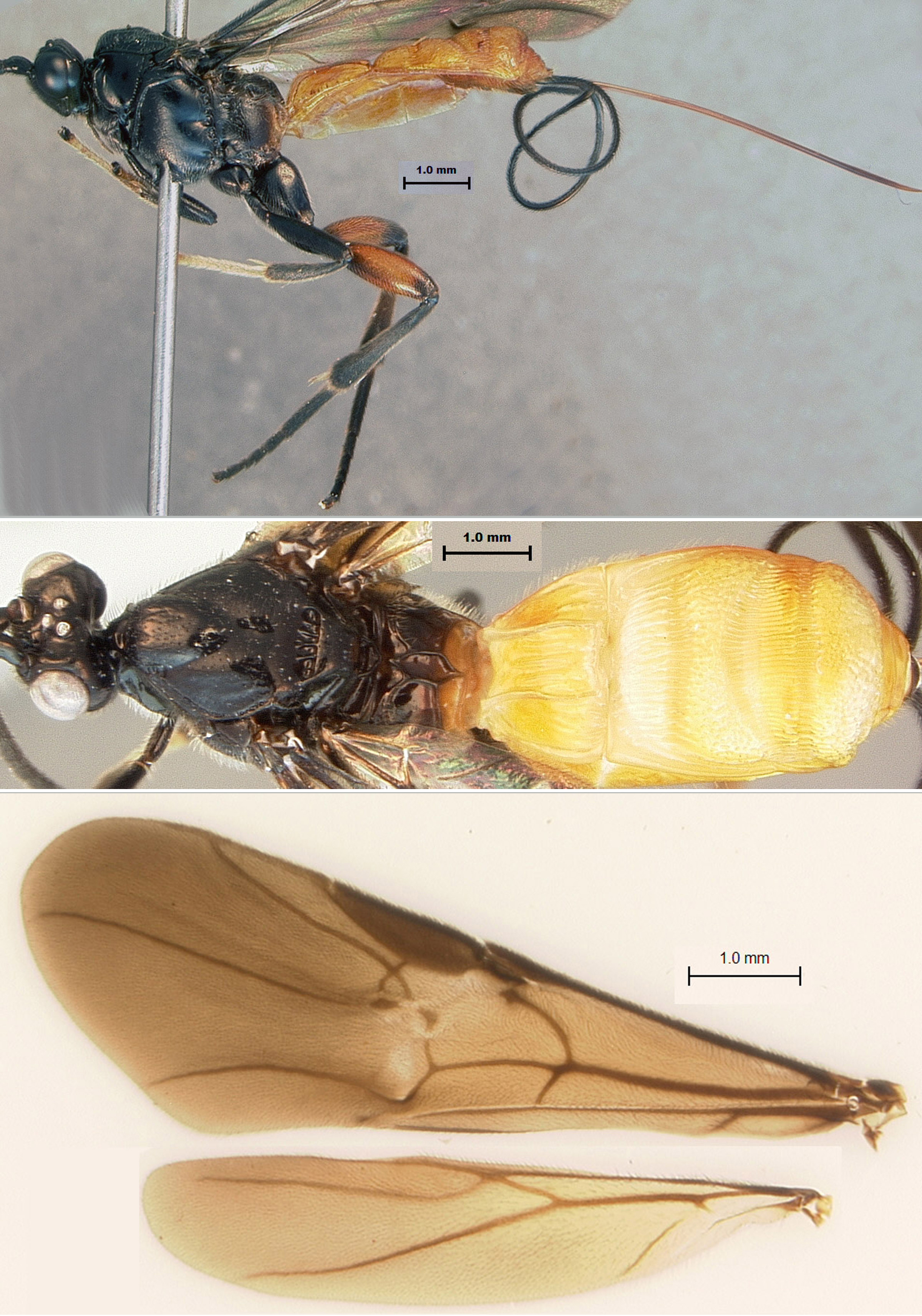

Supplement: Supplementary material 1 — DELTA data matrix, images, and other files [file ZooKeys-130-379-s001.zip › Lytopylus images/sp_1D_long_plate_1A.jpg]

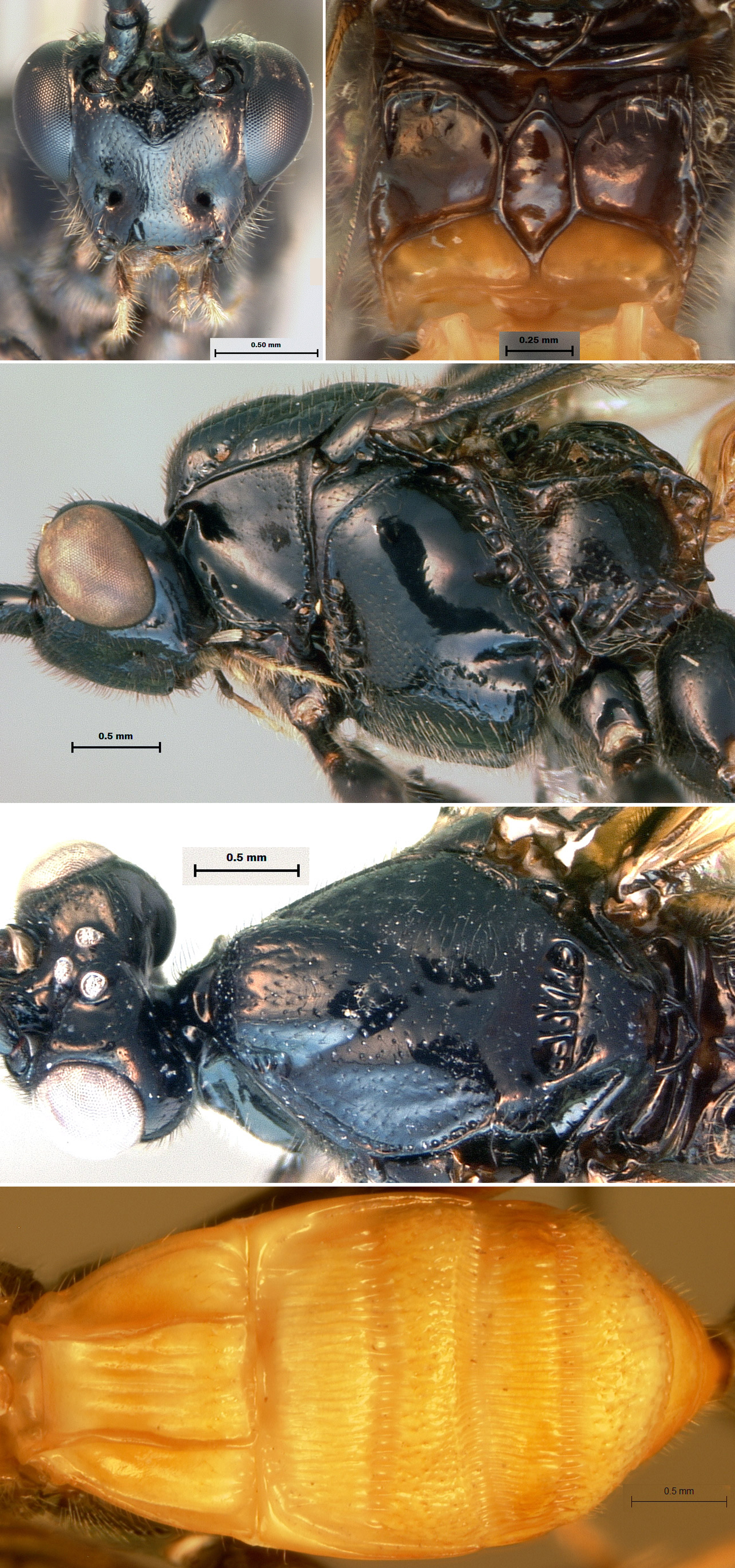

Supplement: Supplementary material 1 — DELTA data matrix, images, and other files [file ZooKeys-130-379-s001.zip › Lytopylus images/sp_1D_long_plate_2A.jpg]

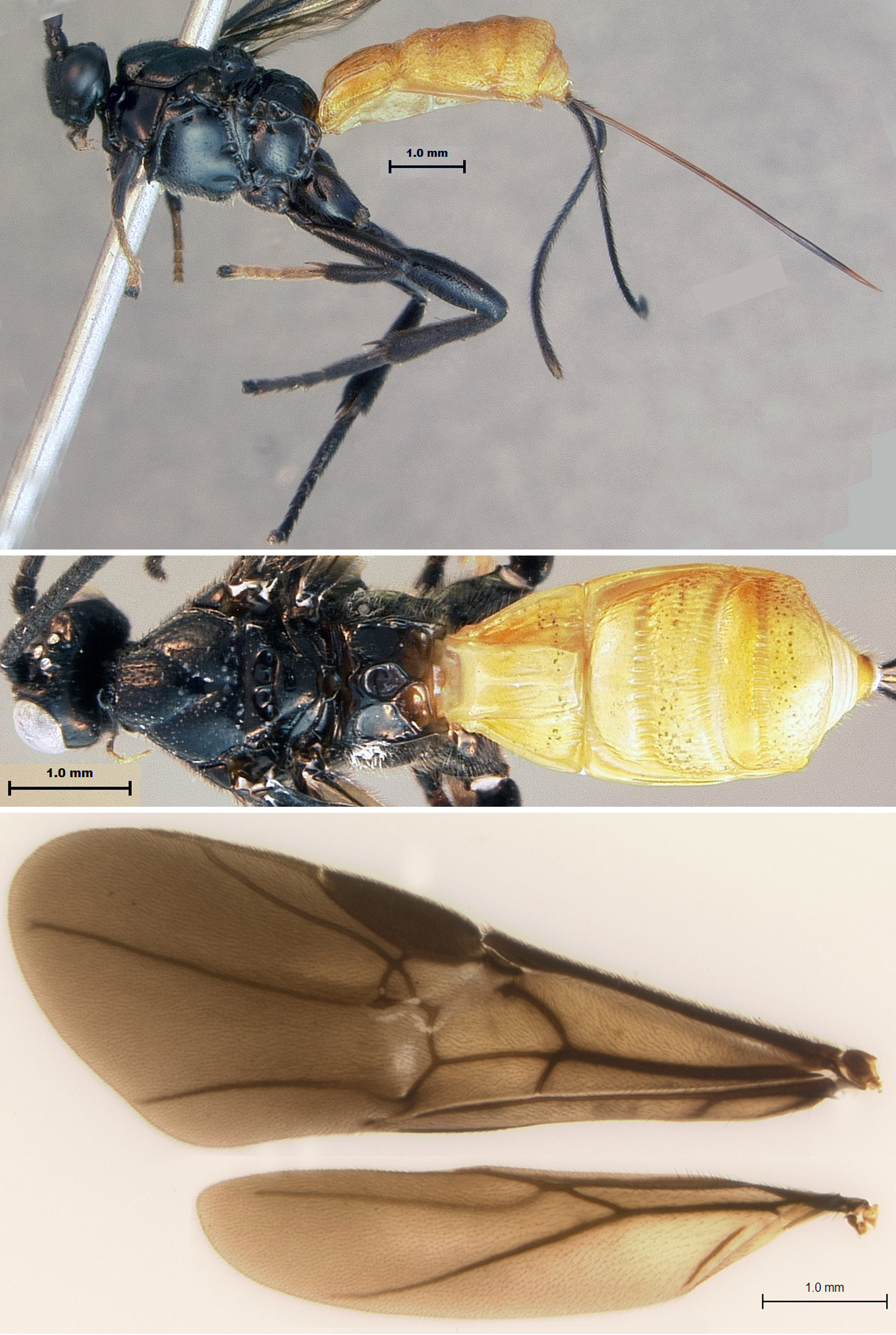

Supplement: Supplementary material 1 — DELTA data matrix, images, and other files [file ZooKeys-130-379-s001.zip › Lytopylus images/sp_1Z_short_plate_1A.jpg]

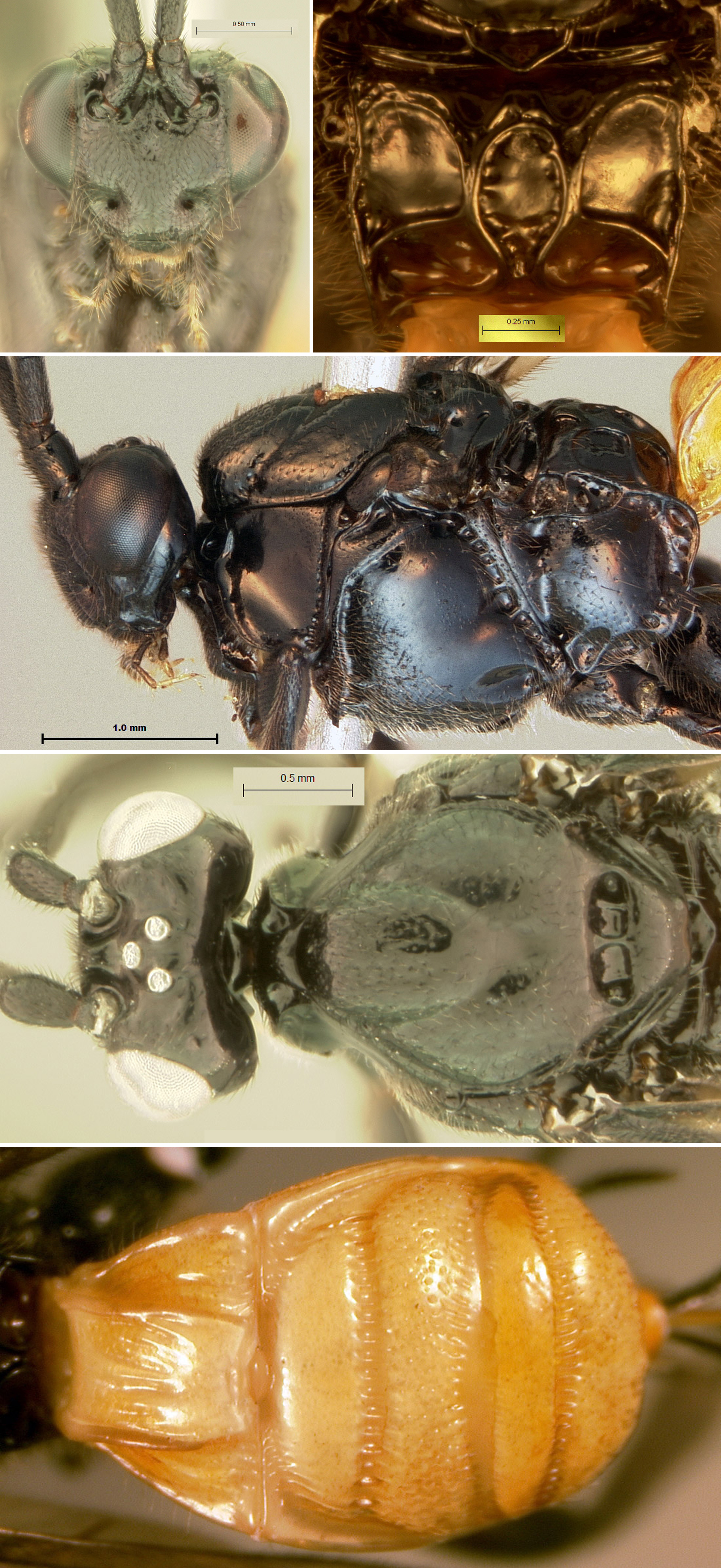

Supplement: Supplementary material 1 — DELTA data matrix, images, and other files [file ZooKeys-130-379-s001.zip › Lytopylus images/sp_1Z_short_plate_2A.jpg]
